# Estimating setback distances for a threatened, cryptic, data-sparse migratory shorebird

**DOI:** 10.1371/journal.pone.0317081

**Published:** 2025-04-23

**Authors:** Birgita D. Hansen, Jodie Honan, Don Stewart, Judi R. Walters, Michael A. Weston

**Affiliations:** 1 Centre for eResearch and Digital Innovation, Federation University, Ballarat, Victoria, Australia; 2 South Beach Wetlands and Landcare Group, Port Fairy, Victoria, Australia; 3 Deakin Marine Research and Innovation Centre, School of Life and Environmental Sciences, Faculty of Science, Engineering and the Built Environment, Deakin University, Burwood, Victoria, Australia; SKUMS: Shahrekord University of Medical Science, IRAN, ISLAMIC REPUBLIC OF

## Abstract

Cryptic fauna species using highly modified habitats face many conservation challenges, with disturbance from human use being an ongoing issue across many global settings. Setbacks or buffers are a key planning tool for protecting habitat, and are often specified under law. However, for many species using modified and urban habitats there are no published data on how wide setbacks should be. Latham’s Snipe (*Gallinago hardwickii*) is a case in point. It is a threatened, cryptic, migratory shorebird that breeds in Japan and spends its non-breeding season almost entirely in Australian wetlands and grasslands. Many sites used by snipe are within urban areas, potentially triggering protections under national law and there is an urgent need for information on setbacks to inform planning and conservation management. The aim of this project was to derive transparent, scientifically-derived buffer recommendations for mitigating disturbance to Latham’s Snipe, by estimating Alert Distances (AD; the first sign of behavioural disruption associated with human proximity) from measures of flush distances (FIDs; the distance at which flight occurs). ADs are almost impossible to observe in this cryptic species which uses dense habitat. We used 1529 FIDs to estimate AD from: (1) a within-species regression of FID against the few available ADs for this species (n =  8), and (2) cross-species associations between AD and FID of Scolopacidae from analysis of an unpublished dataset. FIDs varied between site and observers, so we resampled using bootstrapping to account for this variation and produce estimates of AD. Based on these estimates, we recommend minimum buffer widths between 75 - 110 m, which would prevent 80 - 95% of vigilance responses by Latham’s Snipe, respectively. The methods we employ may be useful in determining appropriate buffer widths for other cryptic fauna species. These buffers should be monitored for effectiveness and adapted as required.

## Introduction

Wetlands and grasslands around the world are under immense pressure from land use change which continues to reduce the extent, and increase fragmentation, of habitat [1,2]. The persistence of species that depend on these habitats relies on ecologically sustainable land use planning, which aims to ensure the ability of species to use these habitats. Species may be sensitive to direct (e.g., loss of habitat) and indirect (e.g., increased disturbance) impacts of land development. Disturbance occurs when a human presence or activity changes a bird’s behaviour or physiology, and therefore disrupts other life-critical activities [3]. Even where the physical attributes of habitat remain, birds might conceivably remain near humans but be disturbed, in which case humans have effectively reduced the quality of that habitat [3]. Birds might move away from humans in which case the effective habitat area is reduced [4]. Generally, the likelihood and intensity of avian responses increases with closer proximity to humans, and understanding the relationships between human proximity and bird response offers a defensible, transparent approach to managing disturbance.

Setbacks or buffers that separate birds from humans can reduce disturbance and enable habitats to be used by birds [5]. While buffer recommendations exist for many species, estimates for many other species are absent or underpinned by few data [5]. Many available buffers recommendations are effectively arbitrary. This is especially the case for highly cryptic species, for which planning decisions occur without adequate information, and which may not therefore adequately protect the species present.

Latham’s Snipe (*Gallinago hardwickii*) is a cryptic medium-sized (~150 g) wetland and grassland shorebird. It is one of 37 migratory shorebird species listed as matters of national environmental significance under Australian national legislation, the *Environment Protection and Biodiversity Conservation Act* (1999). The species was uplisted nationally to Vulnerable in January 2024 in recognition of threatening processes including wetland habitat loss to land development [6]. Latham’s Snipe has a core habitat range in eastern Australia, where it spends the non-breeding period [6]. The known distribution of Latham’s Snipe overlaps substantially with intensively modified landscapes, particularly urban and near urban areas [6]. Latham’s Snipe roost during the day in thickly vegetated wetlands and nearby grasslands. In urban areas these habitats are subject to intense and growing anthropogenic stressors. Snipe habitats within the urban matrix are often in elongated or small habitat patches that interface substantially with areas used extensively by people, by virtue of their edge-interior ratio. Urban development and associated increases in human presence and activity are considered key threats to the species [6].

Data on behavioural responses of birds to humans offer a scientific basis for developing minimum buffer width recommendations to minimise disturbance [3,6]. Avian responses to human approaches scale positively with increasing proximity. The initial response is ‘vigilance’ or ‘alert’; the distance between the bird and the human when the bird becomes vigilant is the alert distance (AD) [3]. If the human approaches more closely, the bird may take flight and ‘flush’ from its location. The distance between the human and the bird when flight occurs is the flight initiation distance (FID) [7]. AD is especially useful for setting buffer distances, as no behavioural disruption (disturbance) normally occurs beyond AD (but see [8]).

While buffer widths should ideally be based on actual AD data, this is not usually possible for cryptic species using thick vegetation, a common situation for many shorebird species [9]. Additionally, AD can be less reliably estimated by different observers [10], or is less repeatable than FID in some birds [11]. While no validated method exists to estimate ADs in such circumstances a variety of alternate approaches have been used, which are described below.

*Alarm Call Distance.* This only applies for species which alarm call reliably, for example when protecting nests or broods, and therefore does not apply to non-breeding Latham’s Snipe. Estimating distance to the point where calls are emitted is impossible for Latham’s Snipe, and such an approach has been suggested to be unreliable because calls could be emitted at any point during a bird’s response to human approach [9].

*Cross-species extrapolations from AD against body mass.* Many comparative analyses indicate that body mass is often associated with species FID/AD (e.g., [3]). Associations may change between geographical areas [12], and extrapolations of AD from body mass relationships proved unrealistic when estimating AD from FIDs of Wood Sandpipers *T. glareola* [9].

*A “fixed slope rule” between AD and FID.* The fixed slope rule suggests AD is about double FID across a range of taxa including birds [13]. The fixed slope rule was applied to estimate ADs from FIDs for Wood Sandpipers and was deemed to result in more realistic estimates of AD than the two abovementioned procedures [9].

Generalised buffer widths for managing disturbance to shorebirds are provided by the Australian Government [14], but while buffer width recommendations exist for some migratory shorebird species (e.g., [15]), there is no explicit guidance on suitable buffer widths to reduce disturbance to Latham’s Snipe. Latham’s Snipe occupy different habitats to most other migratory shorebirds and are rarely observed prior to flushing. Latham’s Snipe may exhibit distinct alert responses (e.g., changed posture, cessation of feeding, freezing) before flushing but these responses are difficult or impossible to detect amid the thick vegetation characterising their habitat [16]. Collection of ADs for the species has therefore proven to be extremely difficult. For cryptic shorebirds in dense habitats, estimating AD indirectly is required, and is best based on the more readily measurable FID, and drawing on known or inferred associations between FID and AD [9].

Decisions regarding buffers for Latham’s Snipe are constantly being made in the context of great uncertainty, which is barely recognised or managed [17]. This study aims to transparently estimate recommended buffer widths to minimise disturbance of Latham’s Snipe using an extensive FID dataset collected as part of a larger survey program. Resultant buffer width recommendations are intended to guide conservation and management actions aimed at protecting habitat for Latham’s Snipe. They offer a starting point for urban wetland habitat management (“something where there is nothing”), and therefore represent an hypothesis, for future researchers and planners to accept, test or advance [18].

## Methods

### Site descriptions and survey methods

This study was conducted across multiple wetland sites, mostly in south-eastern Australia. The sites were selected from a larger national monitoring program on Latham’s Snipe. This ongoing citizen science program commenced in 2014 with about twenty sites in south-west Victoria [19]. The program focuses on determining patterns in distribution and abundance across the species’ Australian range. New sites have been added to the program over time, usually informed by local knowledge. The sites vary greatly in size, extent, topography, elevation, permanence (e.g., permanent artificial, natural ephemeral), water salinity, vegetation type and structure, and landscape context.

Latham’s Snipe surveys were conducted at wetland sites, initially over five consecutive months (October 2014 – February 2015), then three times a season (in mid-September, mid-November and mid-January) from 2015 onwards. The timing of surveys targets key movement and stationary periods across the species’ southerly range. All sites were surveyed on the same day (between sunrise and sunset) to reduce the likelihood of double-counting birds moving between areas overnight. The survey method followed Naarding [20]; observers walked a line transect slowly (1 - 2 km/h) through the site, pausing periodically (for 5 - 30 seconds), and counting birds as they flush. The frequency of pausing depends on vegetation thickness, and may be every minute or so in more thickly vegetated habitats. This ‘walk-pause-walk’ method was adopted because snipe are more likely to flush when a potential predator (i.e., walker) stops, and is the only reliable way to detect snipe [21]. Indeed, frequent observations were made of birds flushing from behind a stationary observer ( < 5 m away).

Observers estimated the distance (m) between themselves and the location where each bird flushed. This “flush distance”, is analogous to flight initiation distance (FID), being the distance between an animal and approaching human when escape is initiated [7]. Observers also estimated vegetation heights (by eye) during their surveys.

The Latham’s Snipe Project is conducted under Federation University Animal Ethics Committee approvals 15-005, 18-004 and 23-005.

### Site selection for FID analyses

Latham’s Snipe surveys were conducted at over 350 national survey sites, however FID was not recorded during all surveys. Sites with reliable FID records were selected based on the following: (a) experience of the observer (i.e., observers that had been involved in the survey program from early in its inception or were otherwise suitably experienced); (b) continuity of the data (representativeness across the survey program, to avoid survey data clustered in a single time period), (c) landscape context (both urban and non-urban sites were included where sufficient data were available; see below); (d) FID records reported as single values (i.e., to remove reported ranges such as when a group of birds flushed at, for example, “10 - 20 m”); and (e) the total number of FID records (set at a threshold of >  20).

Sites were classified into either urban or non-urban adjacent land uses. Classification was done by spatial intersection of point coordinates (representing site centroids) with the Urban Centres and Localities 2021 (polygon) spatial dataset [22] in QGIS. Sites were categorised as non-urban when they fell within the classification “Rural Balance” and urban for all other classifications.

Observers were assigned unique codes either as individual surveyors or as a group. Group codes were associated with the most experienced observer in a group. If a new observer joined an existing individual or group, the existing observer code was used. This coding process recognised that the most experienced observer would influence the estimation of an FID. Where observers included pairings or groupings of experienced people who previously had their own observer code, this pair or grouping of observers was assigned a new code.

### Data analyses

Variation in raw FID values between wetland site and observer code was explored initially by plotting. Based on variation in plots, a negative binomial generalised linear mixed model was fitted with observer code and site as fixed effects, and land use as a random effect. Negative binomial models were calculated using the package *glmmTMB* [23] in R [24]. Model fit was assessed by measures of overdispersion and residual plots. On the basis of model results, raw FID values were resampled with replacement using 10,000 bootstrapped replicates, grouping values based on site and observer code. Resampling was used to generate confidence intervals, while grouping by the main sources of variance (i.e., site and observer; see Results).

### FIDs, estimating ADs and therefore buffer widths

Two metrics were initially calculated (1) 95^th^ percentile of raw FID, and (2) adjusted 95^th^ percentile (i.e., *95*^*th*^*_StDev* =  mean +  1.645 ×  standard deviation) of raw FID (after [5]). These have been used in previous studies to determine buffer widths, usually for non-cryptic species.

A variety of approaches can be used to infer AD from FID in cryptic shorebirds, and while none are unambiguously superior and validation is lacking, they offer an alternative to the metrics above that we consider more suitable for cryptic species. Here, we use two inferential approaches for estimating AD in Latham’s Snipe. First, a small sample of both AD and FID records for Latham’s Snipe (n =  8) obtained from a different study (D.T. Blumstein, *unpubl. data*) were analysed with a simple linear regression, and the regression results used to predict AD from our bootstrapped FID estimates. Second, we used linear regression to understand the relationship between AD and FID across eight scolopacid shorebird species during the non-breeding period in Australia (D.T. Blumstein and MAW *unpubl. data*): *Limosa lapponica* (n =  95), *Calidris ferruginea* (n =  18), *C. ruficollis* (n =  180), *C. acuminata* (n =  79), *Numenius madagascariensis* (n =  49), *N. phaeopus* (n =  13), *Tringa nebularia* (n =  21) and *T. brevipes* (n =  47). We choose these species because they are confamilial with snipe; we are not interested in the evolution associations of FIDs, thus we do not use phylogenetic controls. Two response variables were modelled: 1) the raw 95^th^ percentile (FID) and 2) the adjusted 95^th^ percentile (FID). Based on the linear model coefficients for each of the two FID metrics against AD, we then used the Latham’s Snipe bootstrapped FIDs from the present study to predict ADs.

These two inferential approaches produced three estimates of AD. *Estimate 1* was based on the observed relationship between AD and FID for Latham’s Snipe. *Estimate 2a* was based on the multi-species modelled 95^th^ percentile of AD against FID (excluding Latham’s Snipe), and predicted Latham’s Snipe AD from bootstrapped FID values, and *Estimate 2b* used the same approach but on adjusted 95^th^ percentile (bootstrapped FID values). These three estimates were derived separately for each wetland site and for all data combined. Bootstrapped FID values enabled confidence intervals of AD to be generated (5%, 80%, 90% and 95%). All reported distances are in metres.

## Results

Twenty wetland sites qualified for analysis, with most being in Victoria (six in western Victoria, and five each in Greater Melbourne and eastern Victoria: S1). All were within 30 km of the coast, with most adjacent to estuaries and bays. Only four sites were located outside urban areas; this reflects an emerging pattern in national surveys where Latham’s Snipe congregates in urban wetlands (B. Hansen, *pers.obs*). There were 1529 FID records from these 20 sites. Mean FID did not differ greatly between urban and non-urban wetland sites (urban: 22.2 m, SD =  15.9, 1310 records; non-urban: 26.2 m, SD =  17.0, 219 records). Table 1 lists the general characteristics of the 16 urban and four non-urban sites, the range in vegetation heights at each site, the number of different observers that survey the site, measure of flushing distances obtained during surveys and estimates of AD (see below). Further details on each site are provided in S1 File.

**Table 1:** Flight initiation distances (FID) and estimates of alert distances (AD) for Latham’s Snipe from selected sites in the national survey program. Estimates of AD were derived using three different analytical approaches: (*Est. 1*) Latham’s Snipe dataset regression of AD versus FID for eight snipe (D.T. Blumstein, unpubl. data), (*Est. 2a*) cross-species AD-FID regression using the raw 95^th^ percentile AD and FID, and (*Est. 2b*) cross-species AD-FID regression using the adjusted 95^th^ percentile FID, with *Est. 2a* and *2b* excluding the small sample of Latham’s Snipe data. Median AD estimates plus 5% and 95% confidence intervals are calculated from 10,000 bootstrapped replicates of raw FID, grouping by site and observer.

Site	General wetland characteristics	Vegetation heights (m)	No. Obs.codes	Mean raw FID (SD)	AD Est1 median (5-95% CI)	AD Est2a median (5-95% CI)	AD Est2b median (5-95% CI)
***Urban***							
** Allansford**	Tussocky/ grassy/ weedy freshwater railway easement ponds	0.4–0.7	5	19.0 (13.5)	40.2 (36.6–49.3)	50.4 (45.4–62.1)	57.0 (51.8–62.3)
** Burton’s Reserve**	Tidal, estuarine (tussocks & saltmarsh) marsh	0.1–0.6	1	7.0 (3.0)	11.4 (10.9–16.4)	13.1 (12.5–19.5)	20.3 (17.7–23.0)
** Byron wetlands**	Sewage ponds with mowed edges, thick grasses and fringing trees/ shrubs	0.3–1.0	2	21.1 (18.6)	53.6 (42.9–53.9)	67.7 (53.9–68.1)	61.0 (54.3–67.7)
** Cheltenham Rd Retarding Basin**	Weedy freshwater roadside retention basin	0.3–0.5	1	24.7 (17.3)	54.7 (51.4–87.5)	69.1 (64.9–111.6)	77.5 (64.1–90.8)
** Fox and Pub Lakes**	Tidal, estuarine (saltmarsh) marsh	0.15–0.5	2	31.5 (24.2)	84.7 (55.6–105.5)	108.0 (70.3–134.9)	101.3 (80.1–121.0)
** Melton Botanic Gardens**	Tussocky riparian restoration corridor	0.2–1.5	3	14.6 (7.6)	24.8 (19.8–26.7)	30.5 (24.0–33.0)	37.3 (30.6–42.4)
** Moyne estuary**	Supra-tidal, estuarine (saltmarsh) marsh	0.2–0.5	7	22.6 (17.8)	41.6 (31.9–69.3)	52.2 (39.6–88.1)	68.0 (48.7–86.3)
** Peterborough wetlands**	Tussocky/grassy seasonal freshwater and sinkhole wetlands	0.2–0.5	5	21.5 (13.5)	48.1 (44.8–55.9)	60.6 (56.4–70.7)	61.9 (58.4–65.4)
** Powling Street wetlands**	Kikuyu/reed seasonal freshwater modified wetlands	0.5–0.6	4	28.2 (21.3)	68.2 (57.4–91.0)	86.6 (72.6–116.2)	87.9 (78.7–97.2)
** Railway Place wetland**	Tussocky freshwater retention pond	0.3–0.6	7	16.5 (11.6)	29.2 (23.3–34.3)	36.2 (28.3–42.8)	45.7 (37.7–52.9)
** Retarding Basins Dandenong Valley Hwy**	Freshwater roadside retention basins	0.1–0.5	1	17.2 (12.7)	45.9 (30.6–65.6)	57.8 (38.0–83.3)	56.6 (42.4–69.0)
** Saltwater Creek**	Tussocky/grassy freshwater riparian floodplain marsh	0.1–0.8	5	16.4 12.9)	37.0 (26.2–47.8)	46.2 (32.3–60.3)	51.3 (38.7–61.8)
** Sandy Cove**	Estuarine (saltmarsh) wetland	0.3–0.5	5	24.2 (10.9)	44.7 (39.5–48.3)	56.2 (49.5–60.9)	60.9 (55.9–65.6)
** Silverleaves**	Freshwater-brackish tussocky/kikuyu/ saltmarsh/reed drainage swamp	0.1–0.5	3	24.3 (18.6)	49.6 (33.2–58.7)	62.6 (41.3–74.4)	72.1 (50.7–88.1)
** Tirhatuan Wetlands Conservation Reserve**	Tussocky/grassy freshwater modified wetland	0.2–0.8	1	22.9 (17.5)	56.1 (32.8–109.4)	70.9 (40.8–140.0)	73.8 (51.1–98.6)
** Waterford Wetlands**	Tussocky/grassy freshwater retention basin	0.1–0.5	1	20.2 (9.8)	43.7 (32.8–43.7)	55.0 (40.8–55.0)	54.1 (48.8–59.6)
***Non-urban***							
** Butcher Gap Conservation Park**	Tidal, estuarine (saltmarsh) marsh	0.15–0.3	1	27.2 (18.6)	65.6 (58.2–87.5)	83.3 (73.7–111.6)	84.3 (75.7–93.4)
** Cape Paterson Ecovillage**	Tussocky freshwater wetlands and seasonal marsh (natural & modified/ rehabilitated)	0.2–0.6	1	22.7 (11.5)	43.7 (37.2–60.2)	55.0 (46.5–76.2)	60.8 (49.9–72.2)
** Clifton Creek swamp**	Tussocks and pasture (grazed) seasonal freshwater wetlands	0.1–1.0	1	25.2 (17.4)	54.7 (43.7–101.2)	69.1 (55.0–129.4)	77.7 (61.4–96.7)
** Heart Morass**	Tussocky seasonal freshwater wetlands	0.2–0.5	1	26.7 (9.9)	43.7 (38.3–43.7)	55.0 (47.9–55.0)	63.4 (57.4–68.4)

**Fig 1:**
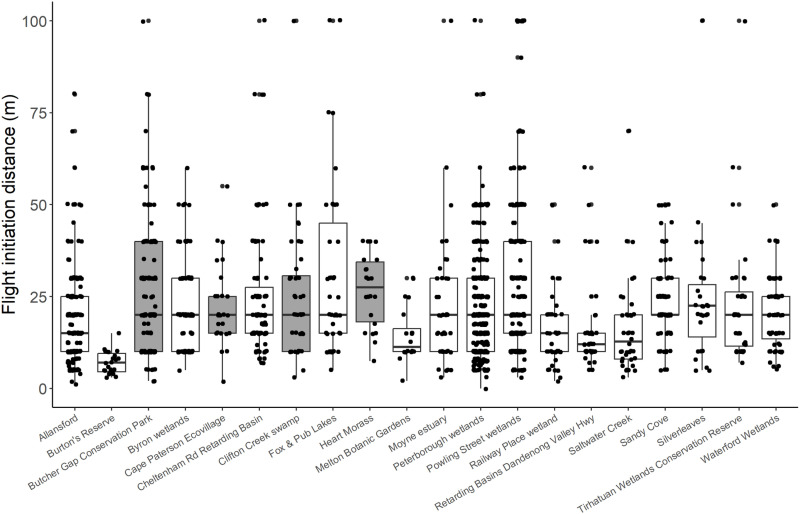
Flight initiation distance records for Latham’s Snipe grouped by wetland site, pooled over observers. Different shading represents wetlands in urban areas (no shading) versus non-urban (grey shading). Points are individual FIDs.

**Fig 2:**
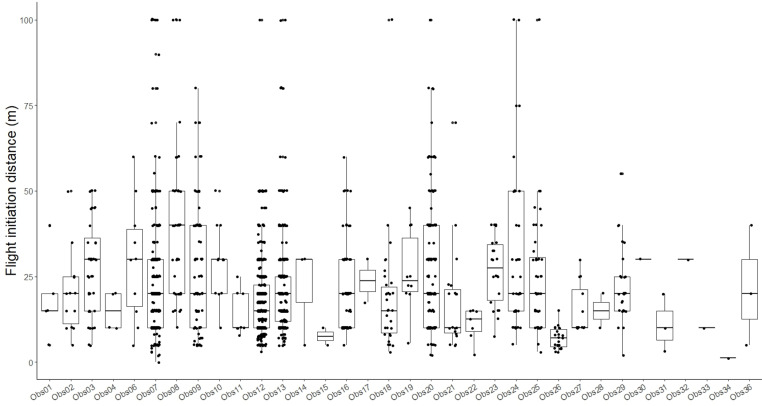
Flight initiation distance records for Latham’s Snipe grouped by observer code, pooled over sites. Points are individual FIDs.

There was a large variation in FID between sites (Fig 1) and, as might be expected when using data from multiple observers, there was considerable variation between observer FID estimates (Fig 2). The negative binomial GLMM revealed significant effects of site (χ^2^ =  56.41, df =  9, P <  0.0001) and observer (χ^2^ =  254.53, df =  33, P <  0.0001), with very little variation attributable to land use (variance =  3.17e^-10^ SD =  1.78e^-05^).

Using these raw FID values, the 95^th^ percentile across all sites and observers was 50.0 m and the adjusted 95^th^ percentile was 49.3 m (S1 File). This was approximately twice the raw 95^th^ percentile (25.9 m, n =  8 observations) from the previous reference study (D.T. Blumstein, *unpubl. data*).

### Estimating AD

AD was highly correlated with FID in the reference study despite the small sample size (R^2^ =  0.98, Adj. R^2^ =  0.97, SE =  1.4, unstandardised slope 1.1 ±  0.1, mean ±  SE; S1). Using the reference study data, a median estimated AD of 44.6 m (5% CI =  16.4 m and 95% CI =  84.7 m) was calculated from the bootstrapped FID in the present study (*Estimate 1* in Table 2).

**Table 2:** Overall estimates of AD, coefficient of variation (COV) and range (maximum minus minimum) across all estimates of AD (and 5, 80, 90 and 95% confidence intervals) based on 10,000 bootstrapped replicates of raw FID. Estimates in bold indicate values deemed suitable for determining disturbance buffer widths, depending on the level of confidence of the decision-maker.

Buffer metric	AD *Est1*	AD *Est2*	AD *Est2b*	COV (%)	Range
**Median**	44.6	56.1	61.6	16.0	17.0
**5% Conf. Int.**	16.4	19.5	24.7	20.8	8.3
**80% Conf. Int.**	59.8	**75.7**	**80.6**	15.1	20.8
**90% Conf. Int.**	72.8	**92.5**	**88.6**	12.3	19.7
**95% Conf. Int.**	84.7	**108.0**	**94.9**	12.2	23.3

**Fig 3:**
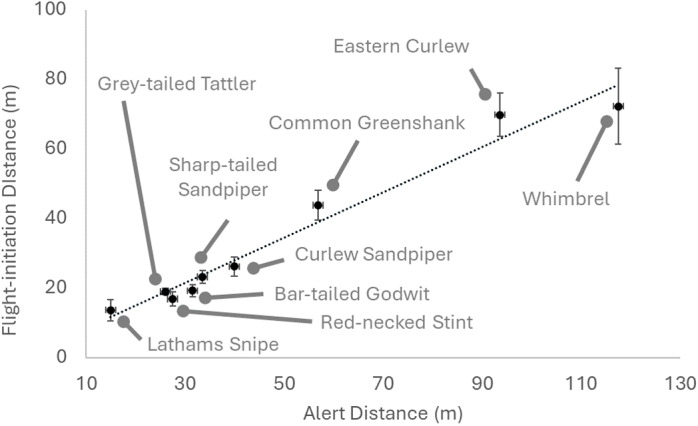
Regression line of mean ( ± SE) flight initiation distance in metres (FID) versus mean ( ± SE) alert distances in metres (AD) for nine shorebird species (unpubl. data). This illustrates the strength of these relationships, and the consistency between this relationship and the small sample of Latham’s Snipe available. Separate regressions to estimate AD from FID excluded the small sample of Latham’s Snipe, and used raw or adjusted 95^th^ percentiles.

There was a very strong relationship between FID and AD across Scolopacid species (see Fig 3). Fig 3 shows means, and includes the small sample of Latham’s Snipe which clearly falls close to the linear predictions of the other taxa (R^2^ =  0.97, Adj R^2^ =  0.96, SE =  6.7, slope 1.5 ±  0.1). Two linear regression models for all species excluding Latham’s Snipe were: AD versus 95^th^ percentile FID (R^2^ =  0.98, Adj R^2^ =  0.98, SE =  8.6, slope 1.4 ±  0.1), and AD versus adjusted 95^th^ percentile FID (R^2^ =  0.96, Adj.R^2^ =  0.95, SE =  11.8, slope 1.4 ±  0.1). These models were used to generate median AD estimates of 56.1 m (*Estimate 2a*) and 61.6 m (*Estimate 2b*), together with the coefficient of variation of those estimates (Table 2). Bootstrapped AD estimates plus the 5/ 95% confidence intervals for individual sites are given in Table 1, and those estimates at a larger range of confidence intervals are provided in S1 File. *Estimates 2a* and *2b* were the largest of the three, ranging from 75.7 m (*Est.2a*) and 80.6 m (*Est.2b*) at a confidence of 80%, to 108.0 m (*Est.2a*) and 94.9 m (*Est.2b*) at a confidence of 95%. As the sample size underpinning *Estimate 1* was very small, and its use would involve extrapolating well beyond the bounds of the data, we recommend using *Estimates 2a and 2b* as the basis for informing appropriate buffer widths to avoid disturbance of snipe.

## Discussion

Buffers around important habitat help reduce disturbance by human activity associated with urban development and are a commonly-used management and planning tool for species protection [3]. In this study, Latham’s Snipe was detected most often in urban wetlands. It is therefore likely that a substantial proportion of the Australian population of snipe will experience human disturbance. To protect snipe from potential impacts, land use planners and managers require guidance on buffer design to reduce disturbance effects on this species. We propose minimum buffer width recommendations of between ~ 75 - 110 m, which can be applied at local scales to help protect snipe using wetland habitats and especially at sites subject to human intrusions, such as those under threat from land use development.

### Use of buffer width recommendations

In this study, we use estimates of AD to provide a range of buffer width recommendations we consider suitable for reducing disturbance impacts on Latham’s Snipe. Our estimates of AD are derived from an extensive dataset of Latham’s Snipe FID collected from a range of sites across south-eastern Australia, suggesting at least some generalisation is reasonable. We also suggest that the estimates we present converge to a reasonable extent; the largest difference between our three estimates was 23 m (lowest COVs were only 12%). We recommend using the largest two of the three estimates (*Estimates 2a* and *2b*), which were 75.7 m (*Est.2a*) and 80.6 m (*Est.2b*) at a confidence of 80%, and 108.0 m (*Est.2a*) and 94.9 m (*Est.2b*) at a confidence of 95%. This conservative approach reflects other recommendations about developing buffer zones (e.g., [7]) and produced buffer widths consistent with other studies. For example, a study on shorebirds using staging sites in North America determined buffer widths for nine species ranging from 61 m for smaller species like Least Sandpiper *Calidris minutilla* (approximately 30 g) to 186 m for larger species like Grey Plover *Pluvialus squatarola* (~160 - 275 g) [15].

The resampling approach used here allowed the generation of AD estimates that account for variation in raw FIDs attributable to site and observer effects, and the production of confidence intervals. These confidence intervals allow decision-makers to decide what risk they wish to take when making buffer width decisions to address potential impacts of human proximity associated with land development on snipe. A buffer width based on the 95% confidence interval will reduce the risk that snipe are negatively affected by disturbance, but will require larger areas to be protected as habitat. Therefore, buffer designations represent a trade-off and managers must decide what level of risk they consider acceptable when implementing these widths.

Under the *Environment Protection and Biodiversity Conservation (EPBC) Act,* a precautionary principle to land development is advised in instances where there is uncertainty about the potential for significant impacts on matters of national environmental significance such as snipe. Consistent with this principle, we use estimates of AD to provide a more conservative measure than FID. This is common practice [9] and allows for the possibility that the disturbance effects on Latham’s Snipe occur at greater distances than FID [7]. From a management perspective, however, widths recommended here should be considered a minimum when designing buffers to mitigate disturbance [5]. If buffers are altered in some way (e.g., intersected with walkways, creating “buffer creep”), then widths may need to be increased, and human activity may require additional restrictions [25,26]. Larger vegetated buffers are also more effective for other management purposes such as pollution and nutrient retention, and provision of habitat for other species [27,28].

Uplisting of Latham’s Snipe to Vulnerable under the EPBC Act was underpinned by improved knowledge about contemporary threats to the species, the most notable being habitat loss caused by land development, which brings with it disturbance [6]. The application of appropriate setbacks will be a mitigation tool for reducing significant impacts caused by land development. At present, setbacks for species like Latham’s Snipe are determined arbitrarily, if at all. Here we present quantitative data to inform the mitigation of significant impacts that may result from proposed land use developments referred under the EPBC Act. This approach could equally be applied to other cryptic species in the EPBC Act Significant Impact Guidelines [14], and information exists for other species [12] that could support the development of a database for recommended buffer widths.

### Caveats, potential improvements and opportunities for refinement

There are a number of caveats with the approaches used here. First, FIDs were estimated by eye, and there was variation in FID estimates among observers. Ideally, FID should be measured using laser range-finders to improve the accuracy (given that pacing is impractical for snipe; [3]). Many birds including shorebirds may exhibit non-flying escape responses such as walking or running which are adjusted in relation to the nature of the approach threat [29]. Non-flight responses were not detectable in the thick vegetation used by Latham’s Snipe, and may have occurred at distances which exceeded our measurement of FID. The nature of snipe responses to an approaching threat would ideally be investigated, perhaps using cameras on focal birds [8] or radio-tagged individuals [30].

Site-based context may influence response distances of snipe. The FID-AD calculations assume that detectability of snipe was uniform within and across sites. This is unlikely, as flushing distances varied over sites, which in themselves varied in vegetation structure and height (Table 1). We speculate that this variation between sites reflects major structural differences in vegetation at each the affects snipe alert responses. For example, sites that experienced high levels of spring rainfall often had exceptional vegetation growth, resulting in tall vegetation with thick cover – where this occurred, flushing distances appeared to be shorter (B. Hansen, *pers. obs*). Conversely, in sites that have shorter vegetation like Fox and Pub Lakes (estuarine sites with generally low saltmarsh: Table 1; Supplementary material S1 File), snipe may flush at longer distances, presumably due to their perception of cover and visibility of oncoming threats. However, this pattern does not appear to be consistent and determining the relationship between vegetation characteristics and flushing distance would require collection of systematic detailed vegetation data at the exact locations where birds flushed. Applying this approach at a large scale to multiple volunteer observers was not possible. Few volunteers had the skill, time, equipment or inclination to collect laser range-finder FID and structured vegetation data when conducting surveys (B. Hansen, *pers. obs.*). Further study could investigate detection probability across a range of habitat types and survey conditions to determine relationships between snipe flushing observations and presence, and whether this is affected by environmental factors [31]. Nevertheless, we chose a conservative approach through recommending use of the longest AD estimates (at 80 - 95% confidence intervals), which have been generated across multiple sites of varying size, vegetation height, and structure, to allow for the possibility of uncertainty caused by imperfect detection.

Any contexts which alter response distances may alter suitable buffer recommendations of snipe. We evoked snipe flushes by walking through habitat, but many other bird species may exhibit longer FIDs to other stimuli, such as dogs [32,33]. Pre-migratory restlessness and other temporal events may also influence FID [34], but our estimates deliberately span the non-breeding period (so exclude pre-migration periods). We also note that in general, buffer designations are required for implementation year round.

## Conclusion

We have presented transparent, data-driven estimates of candidate buffer width designations to prevent disturbance of Latham’s Snipe by humans. We are unaware of any other buffer designations for this species, and note only a handful of buffer estimates exist for ground birds using thick cover [9]. Where implemented, these buffers should be validated to establish their effectiveness; they should be regarded as working hypotheses which can be further refined using site-based field assessments. Our approach might usefully be applied to other cryptic species.

## Supporting information

S1 File S1 Hansenetal snipe FID SupportingInformation 05122024. (PDF)

## References

[1] Ballut-Dajud GA, Sandoval Herazo LC, Fernández-Lambert G, Marín-Muñiz JL, López Méndez MC, Betanzo-Torres EA. Factors affecting wetland loss: A review. Land. 2022;11:434.

[2] Bardgett RD, Bullock JM, Lavorel S, Manning P, Schaffner U, Ostle N, et al. Combatting global grassland degradation. Nat Rev Earth Environ. 2021;2:720–35. doi: 10.1038/s43017-021-00112-3

[3] Weston MA, McLeod EM, Blumstein DT, Guay PJ. A review of flight-initiation distances and their application to managing disturbance to Australian birds. Emu-Austral Ornithology. 2012;112:269–86.

[4] Antos MJ, Ehmke GC, Tzaros CL, Weston MA. Unauthorised human use of an urban coastal wetland sanctuary: Current and future patterns. Landsc Urban Plan. 2007;80(1–2):173–83.

[5] Guay P-J, van Dongen WF, Robinson RW, Blumstein DT, Weston MA. AvianBuffer: An interactive tool for characterising and managing wildlife fear responses. Ambio. 2016;45(7):841–51. doi: 10.1007/s13280-016-0779-4 27055852 PMC5055477

[6] Hansen B, Ura T, Tajiri H, Dutson G. Latham’s snipe gallinago hardwickii. Act Plan Aust Birds 2020. 2020;297–300.

[7] Blumstein DT. Flight-initiation distance in birds is dependent on intruder starting distance. J Wildl Manag. 2003;67:852–7.

[8] Charuvi A, Lees D, Glover HK, Rendall AR, Dann P, Weston MA. A physiological cost to behavioural tolerance. Behav Processes. 2020;181:104250. doi: 10.1016/j.beproc.2020.104250 32971223

[9] Whitfield D, Rae R. Human disturbance of breeding wood sandpipers tringa glareola: Implications for alert distances in prescribing protective buffer zones. Ornis Fennica. 2014;91:57–66.

[10] Guay PJ, McLeod EM, Cross R, Formby AJ, Maldonado SP, Stafford-Bell RE, et al. Observer effects occur when estimating alert but not flight-initiation distances. Wildl Res. 2013;40:289–93.

[11] Hammer TL, Bize P, Saraux C, Gineste B, Robin JP, Groscolas R, Viblanc VA. Repeatability of alert and flight initiation distances in King Penguins: Effects of colony, approach speed, and weather. Ethology. 2022;128:303–316.

[12] Weston MA, Radkovic MA, Kirao L, Guay P, Van Dongen W, Malaki P, et al. Differences in flight initiation distances between African and Australian birds. Animal Behaviour. 2021;179:235–45.

[13] Gulbransen D, Segrist T, Del Castillo P, Blumstein DT. The fixed slope rule: An inter‐specific study. Ethology. 2006;112(12):1056–61.

[14] Commonwealth of Australia. EPBC Act Policy Statement 3.21—Industry guidelines for avoiding, assessing and mitigating impacts on EPBC Act listed migratory shorebird species. Department of the Environment. Canberra, ACT. 2015. Available from: http://www.environment.gov.au/epbc/publications/migratory-shorebirds.html

[15] Koch SL, Paton PWC. Assessing anthropogenic disturbances to develop buffer zones for shorebirds using a stopover site. J Wildl Manag. 2014;78:58–67.

[16] Higgins PJ, Davies SJJF. (Editors) Handbook of Australian, New Zealand and Antarctic Birds. Volume Three - Snipe to Pigeons. Oxford University Press: Melbourne, Victoria; 1996.

[17] Henderson J. Managing uncertainty for preventive conservation. Stud Conserv. 2018;63(sup1):108–12. doi: 10.1080/00393630.2018.1479936

[18] Elliott-Graves A. The value of imprecise prediction. Philos Theory Pract Biol. 2020.

[19] Hansen B, Ura T, Tajiri H. Insights into migration and distribution of Latham’s Snipe. Tattler. 2022;51:22–4.

[20] Naarding JA. Latham’s snipe, gallinago hardwickii, in Australia and Japan. RAOU Rep Ser. 1986;24:1–74.

[21] Naarding JA. Latham’s snipe (Gallinago hardwickii) in southern Australia. 1983.

[22] Australian Bureau of Statistics. Australian statistical geography standard (ASGS) edition 3. 2021. [cited 5 December 2024]. Available from: https://www.abs.gov.au/statistics/standards/australian-statistical-geography-standard-asgs-edition-3/jul2021-jun2026

[23] Brooks ME, Kristensen K, van Benthem KJ, Magnusson A, Berg CW, Nielsen A, et al. glmmTMB balances speed and flexibility among packages for zero-inflated generalized linear mixed modeling. The R Journal. 2017;9(2):378. doi: 10.32614/rj-2017-066

[24] R Core Team. R: A Language and Environment for Statistical Computing. R version 4.3.3. (R Foundation for Statistical Computing, Vienna, Austria). 2024. Available from: https://www.R-project.org/RCoreTeam

[25] Miller JR, Wiens JA, Hobbs NT, Theobald DM. Effects of human settlement on bird communities in lowland riparian areas of Colorado (USA). Ecol Appl. 2003;13(4):1041–59. doi: 10.1890/1051-0761(2003)013[1041:EOHSOB]2.0.CO;2

[26] Weston MA, Antos MJ, Glover HK. Birds, buffers and bicycles: A review and case study of wetland buffers. Vic Nat. 2009;126:79–86.

[27] Castelle AJ, Johnson AW, Conolly C. Wetland and stream buffer size requirements - A review. J Environ Qual. 1994;23(5):878–82. doi: 10.2134/jeq1994.00472425002300050004x 34872206

[28] Hansen BD, Reich P, Lake PS, Cavagnaro T. Challenges in applying scientific evidence to width recommendations for riparian management in agricultural Australia. Ecol Manag Resto. 2015;16:50–7.

[29] Lethlean H, Van Dongen WF, Kostoglou K, Guay P-J, Weston MA. Joggers cause greater avian disturbance than walkers. Landsc Urban Plan. 2017;159:42–7. doi: 10.1016/j.landurbplan.2016.08.020

[30] Karlsson J, Eriksson M, Liberg O. At what distance do Wolves move away from an approaching human? Can J Zool. 2007;85:1193–97.

[31] Conway CJ. Standardized North American marsh bird monitoring protocol. Waterbirds. 2011;34:319–46.

[32] Glover HK, Weston MA, Maguire GS, Miller KK, Christie BA. Towards ecologically meaningful and socially acceptable buffers: Response distances of shorebirds in Victoria, Australia, to human disturbance. Landsc Urban Plann. 2011;103(3–4):326–34.

[33] McLeod EM, Guay P-J, Taysom AJ, Robinson RW, Weston MA. Buses, cars, bicycles and walkers: The influence of the type of human transport on the flight responses of waterbirds. PLoS One. 2013;8(12):e82008. doi: 10.1371/journal.pone.0082008 24367498 PMC3867343

[34] Mikula P, Díaz M, Albrecht T, Jokimäki J, Kaisanlahti-Jokimäki M-L, Kroitero G, et al. Adjusting risk-taking to the annual cycle of long-distance migratory birds. Sci Rep. 2018;8(1):13989. doi: 10.1038/s41598-018-32252-1 30228370 PMC6143617

