# Supplementary material for: Estimating setback distances for a threatened, cryptic, data-sparse migratory shorebird
Source: PLoS One. 2025 Apr 23;20(4):e0317081. doi: 10.1371/journal.pone.0317081 (PMC12017478; doi:10.1371/journal.pone.0317081)
Supplement: S1 File — (PDF) [file pone.0317081.s001.pdf]

Supplementary Material: Estimating setback distances for a threatened, cryptic, data-sparse migratory shorebird

Birgita D. Hansen [1*], Jodie Honan [2], Don Stewart[2], Judi Walters[1], Michael A. Weston[3]

[1] *Centre for eResearch and Digital Innovation, Federation University, Ballarat, Victoria 3353*

[2] *South Beach Wetlands and Landcare Group, Port Fairy, Victoria*

[3] *Deakin Marine Research and innovation Centre, School of Life and Environmental Sciences, Faculty of Science, Engineering and the Built Environment, Deakin University, Burwood, Victoria 3125*

* b.hansen@federation.edu.au

B. Hansen https://orcid.org/0000-0003-3608-7622

M. Weston https://orcid.org/0000-0002-8717-0410

Keywords: Alert distance, Environment Protection and Biodiversity Conservation Act, flight initiation distance, Latham's Snipe Project, vegetated buffer

S1. Site descriptions and photographs

Multiple sets of coordinates are provided for some sites, corresponding to distinct water features that are used interchangeably by Latham's Snipe (i.e. the area used regularly by snipe on a daily basis, effectively representing an ecological site). For example, Peterborough wetlands contains 11 distinct water bodies all occurring within the township boundary. Individual waterbodies are aggregated into a "reporting area" for that site.

Allansford, Victoria (-38.3899511, 142.5969533)

Railway easement containing three adjacent waterbodies. Characterised by tussocks, weeds, *Triglochin* sp. 0.4-0.7 m in height; deeper areas containing reeds.

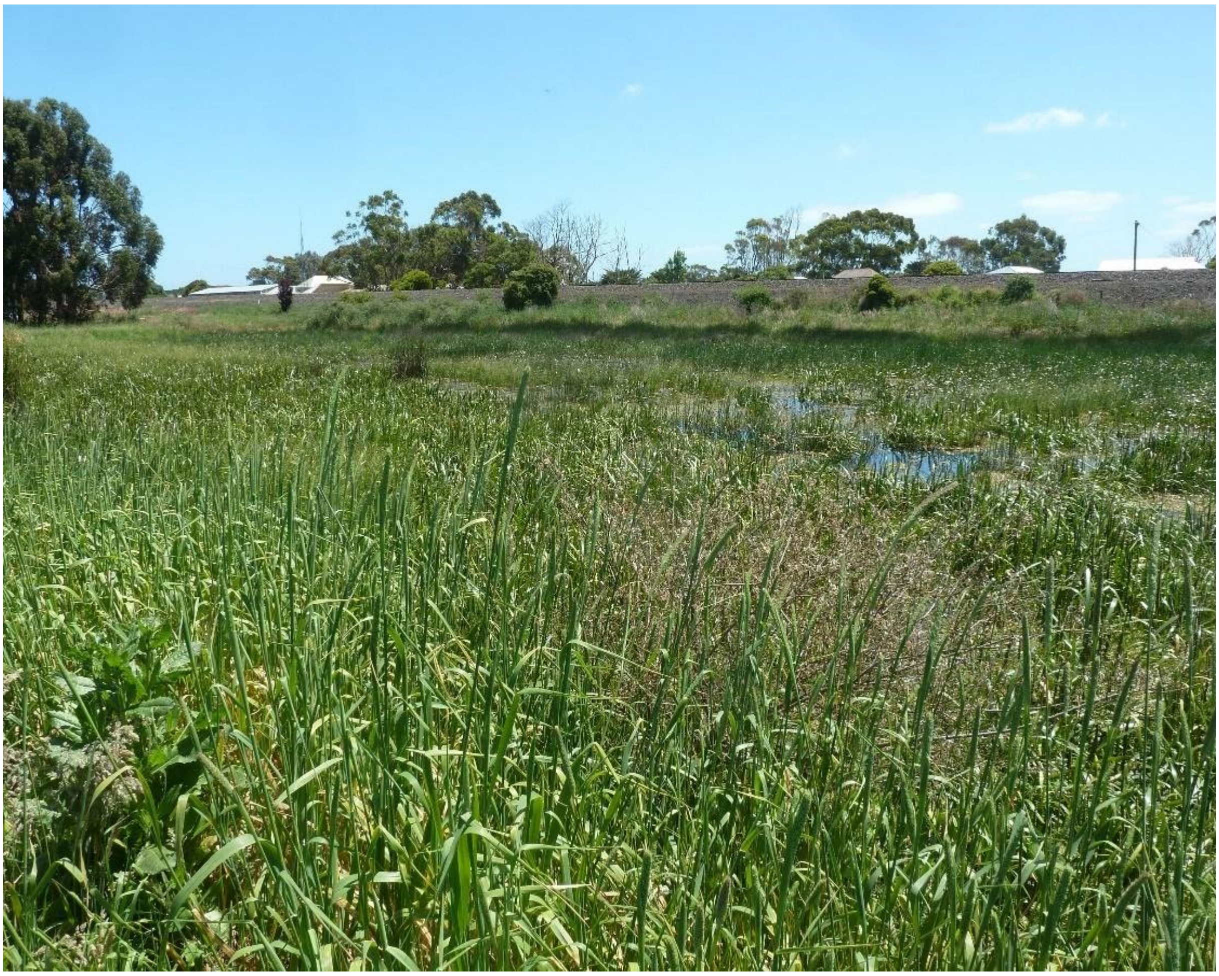

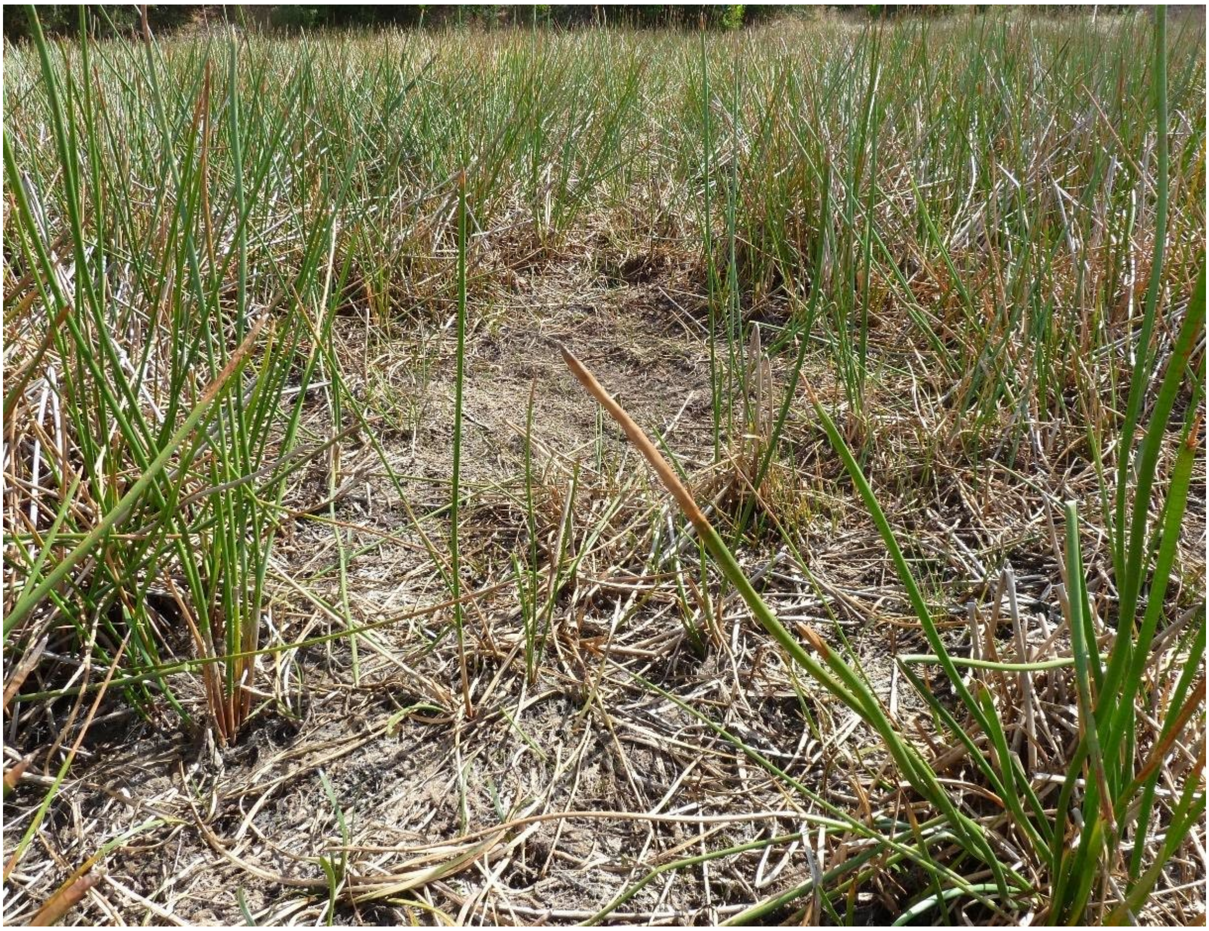

Burton's Reserve, Tasmania (-43.164456, 147.081052)

Site characterised by native tussock grasses, rushes/sedges, samphire 10 – 60 cm; Tidal pools 20 – 50 cm; fringing vegetation is Melaleuca, Acacia, some Eucalypts and parkland;. Photos courtesy of Philip Emery.

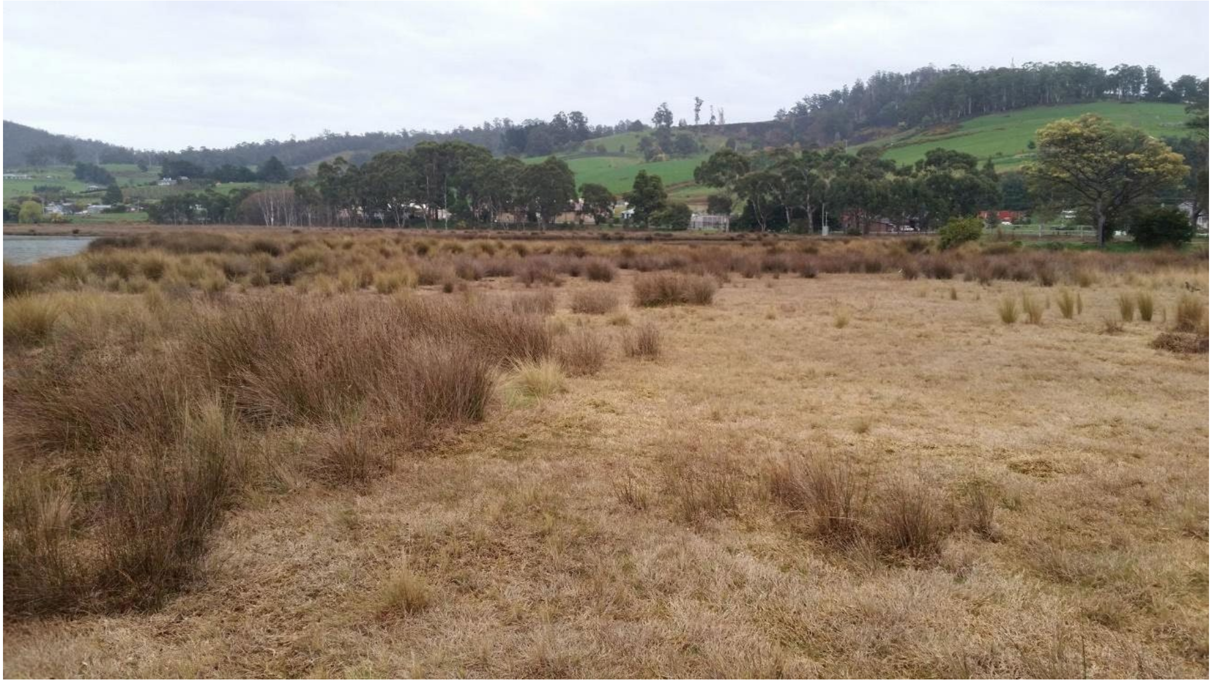

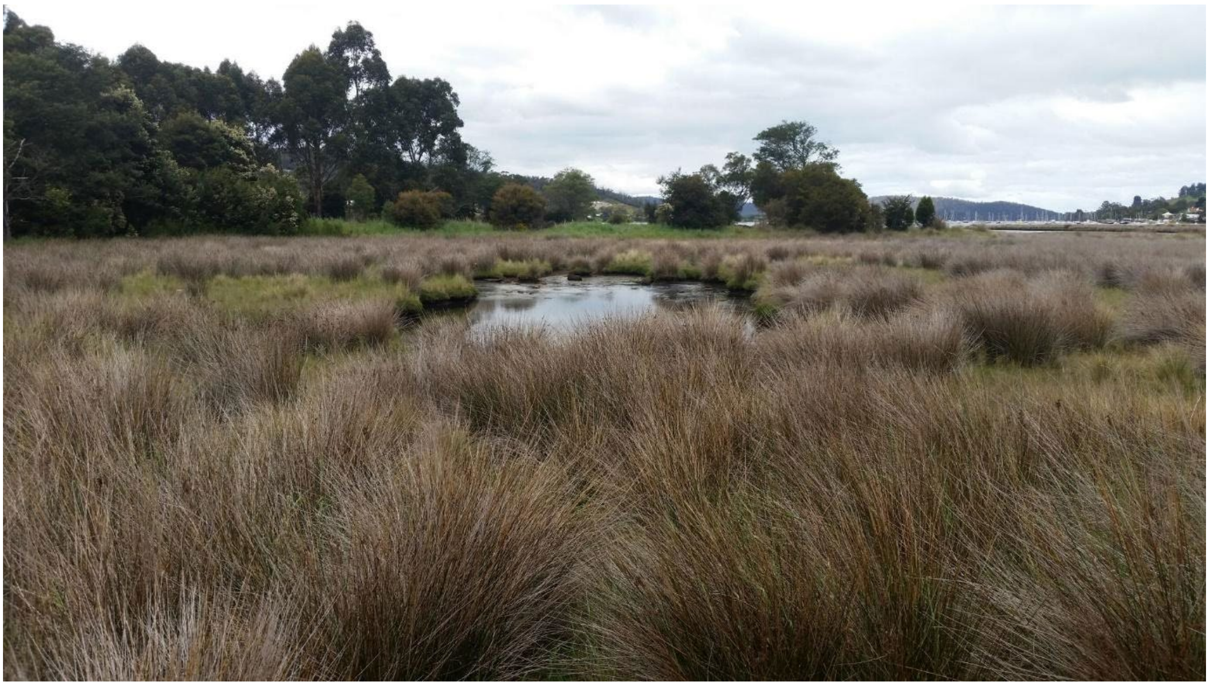

Butcher Gap Conservation Park, South Australia
(-36.8835467, 139.8021874; -36.8784, 139.8173)

Estuarine wetland containing saltmarsh and rushes, samphire ~0.3 m, *Sarcocornia quinqueflora*, *Selliera radicans*, *Sauda australis*, *Distichlis disticophylla*, *Ghania trifidia*, *Juncus kraussii*, *Stipa* spp. Up to 1 m; fringing vegetation *Melaleuca halmaturorum*, *Leucopogon parvifloris*, *Olearia* sp., *Acacia longifolia*. Photos courtesy of Vicki Natt.

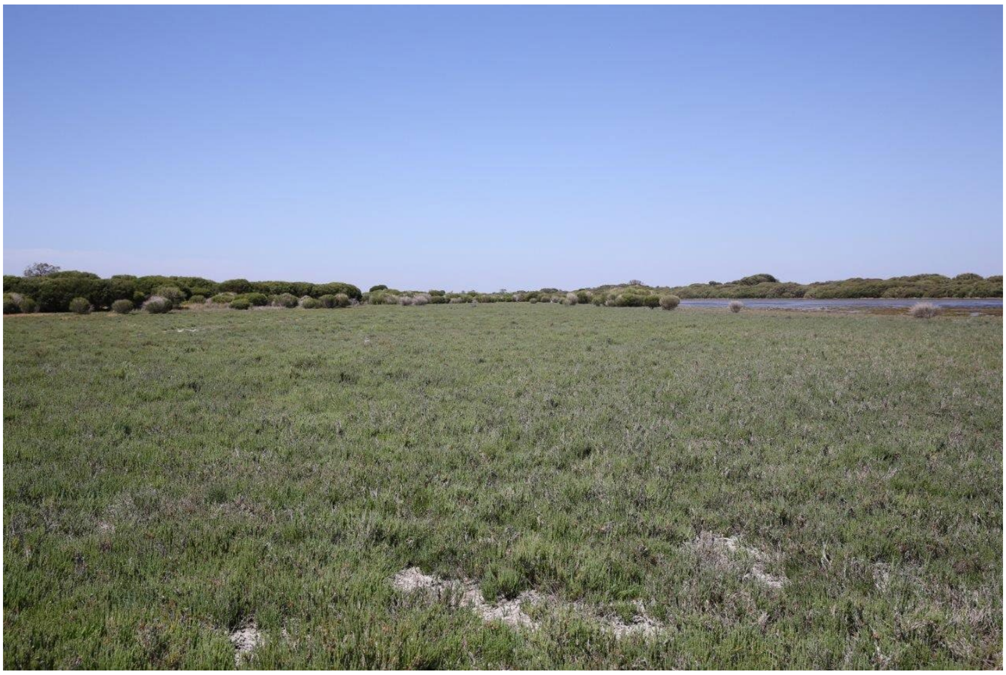

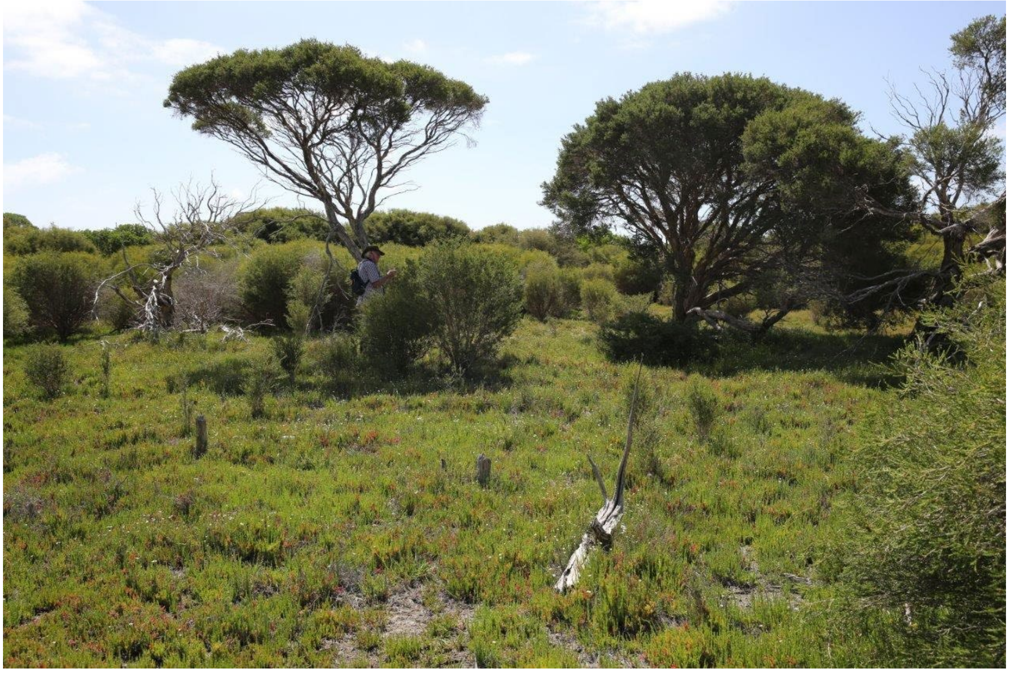

Byron Wetlands, New South Wales (-28.6271978, 153.5781117)

Site is a modified wetland characterised by a series of constructed cells that form the Byron Shire Sewage Treatment Plant. Between cells is mown (<0.1 m) and longer grass, and cell edges consist of small areas of grass and weeds (para grass and setaria), native sedges, rushes, ferns, grasses & swamp hibiscus. Typical vegetation height is 0.3-1.0 m. Paperbark trees and open woodlands are dispersed throughout the sites. Photos courtesy of Jan Olley.

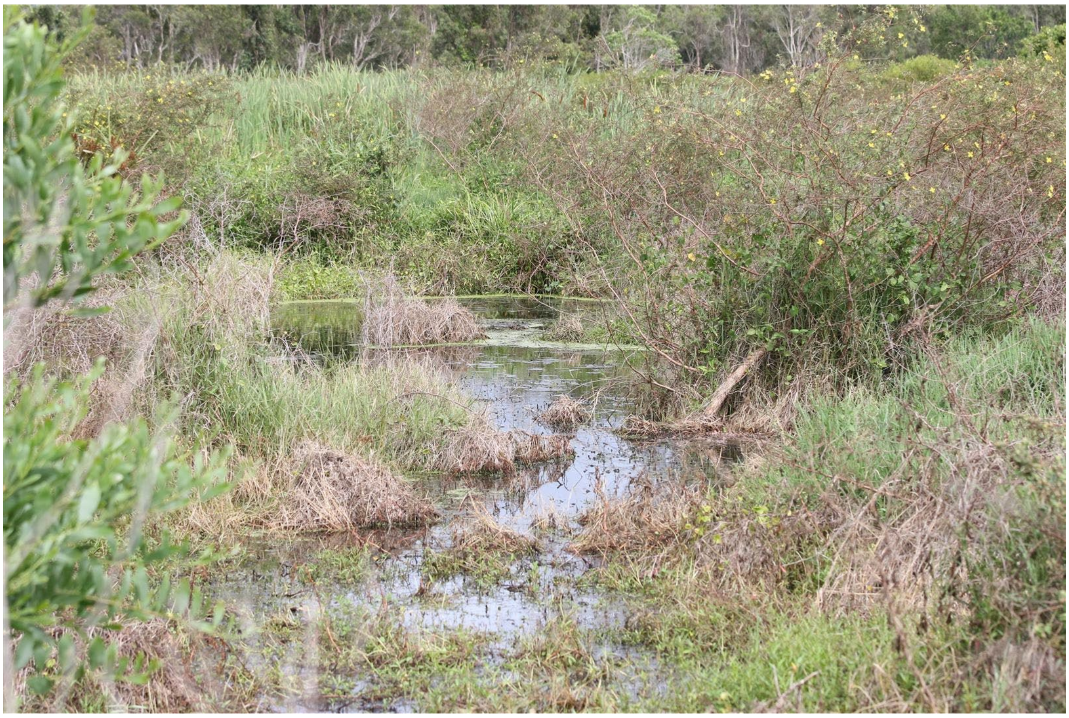

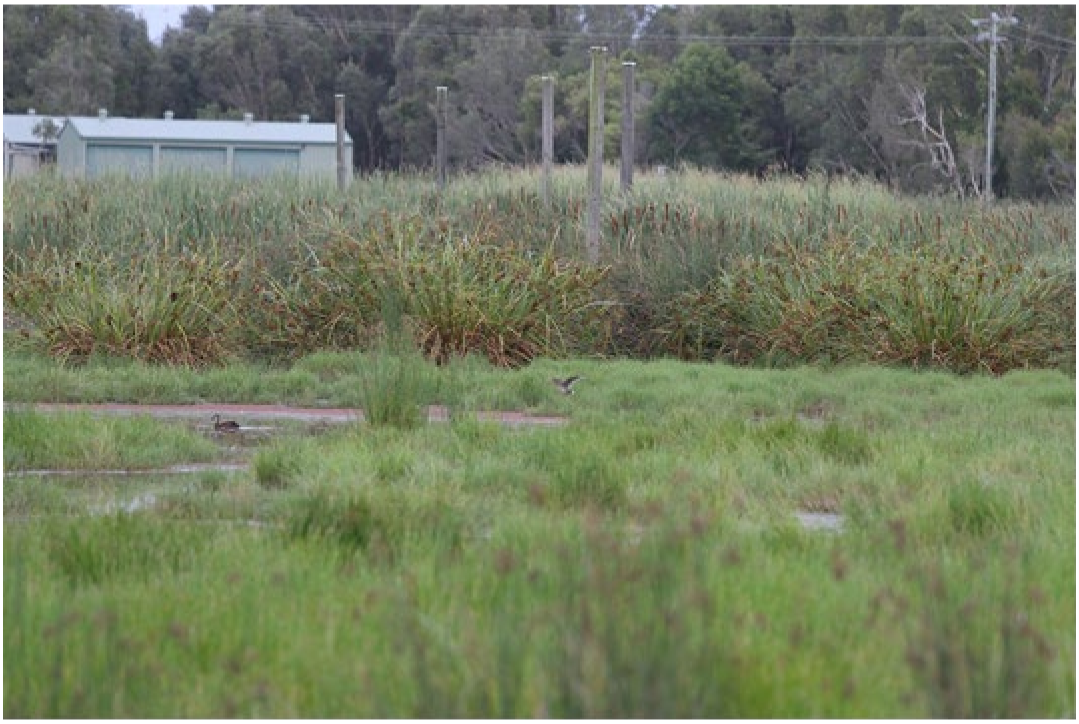

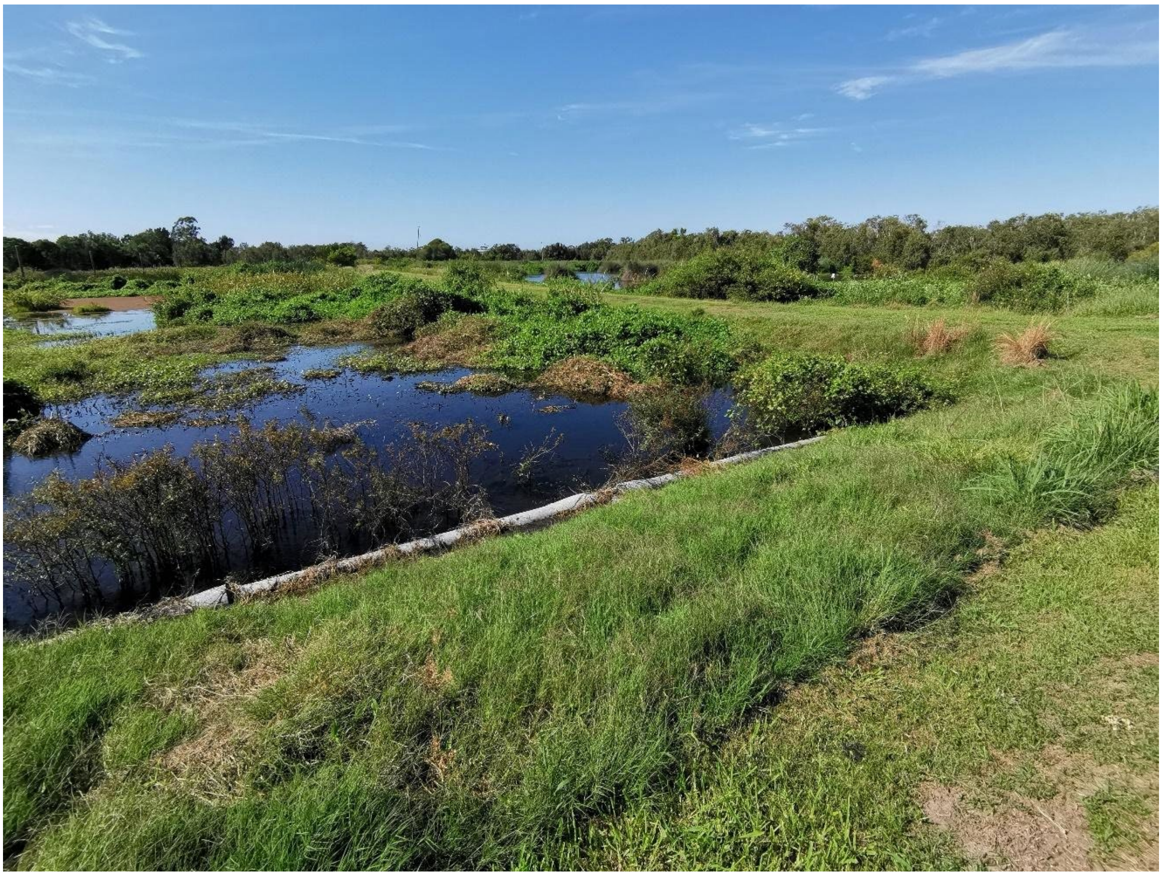

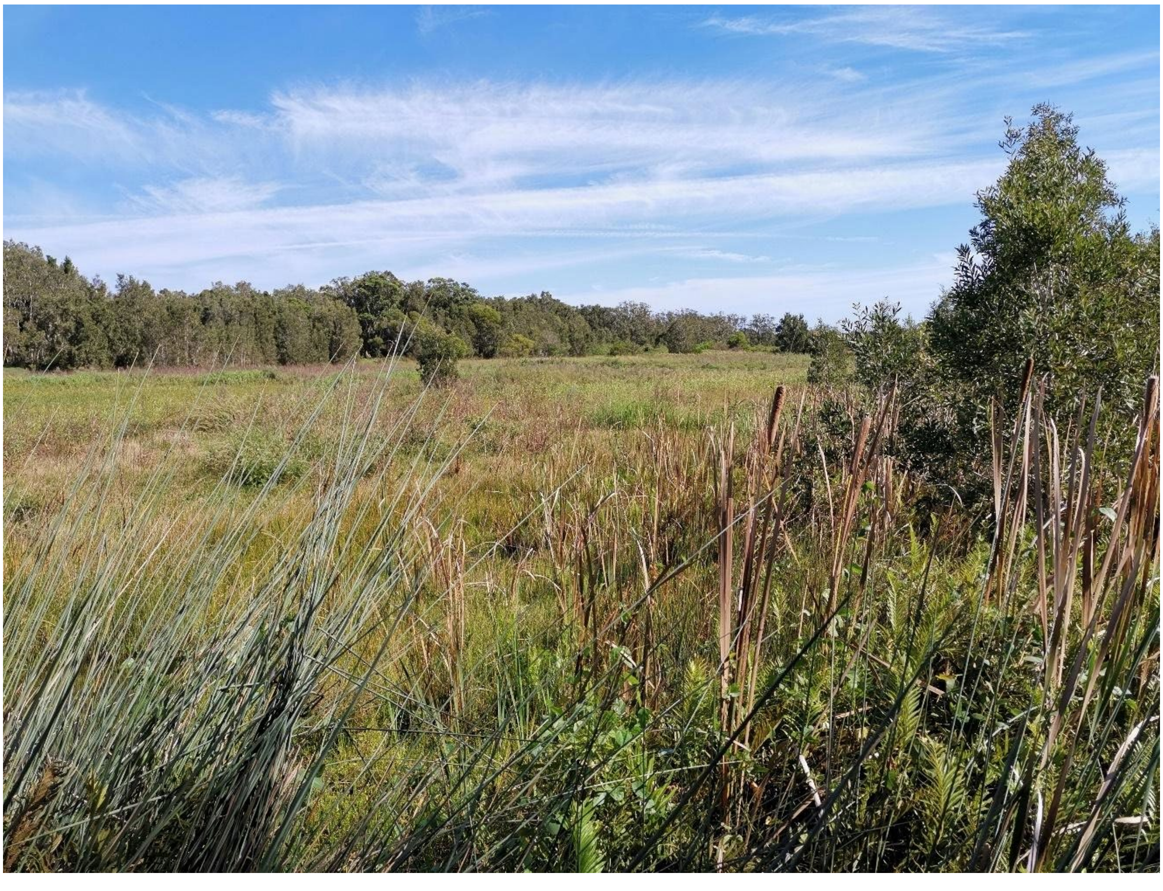

Cape Paterson Ecovillage, Victoria
(-38.6708489, 145.6061176; -38.670027, 145.6037006; -38.6687887, 145.602585;
-38.6682824, 145.6011133; -38.6691245, 145.6000851; -38.6695, 145.6031;
-38.673327, 145.605701)

Site contains several constructed, rehabilitated and natural wetlands and seasonally-inundated marsh. Modified wetlands contains sedges and rushes surrounded by introduced grasses, vegetation height typically 0.2-0.8 m. Public walkways occur adjacent to some wetlands (used by walkers, cyclists, dogs on leads). Seasonal marshes are predominantly grasses typically 0.2-0.6 m, either surrounded by thick coastal bush and heath, or having adjacent coastal heathland. Photos courtesy of David Hartney.

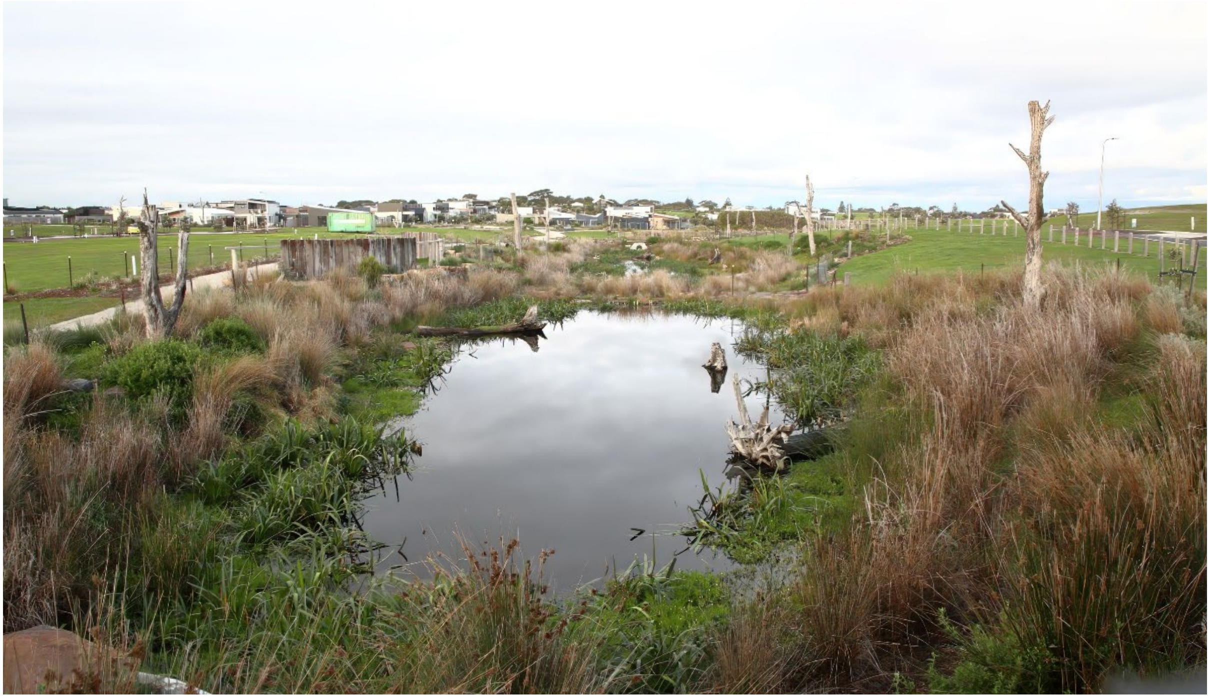

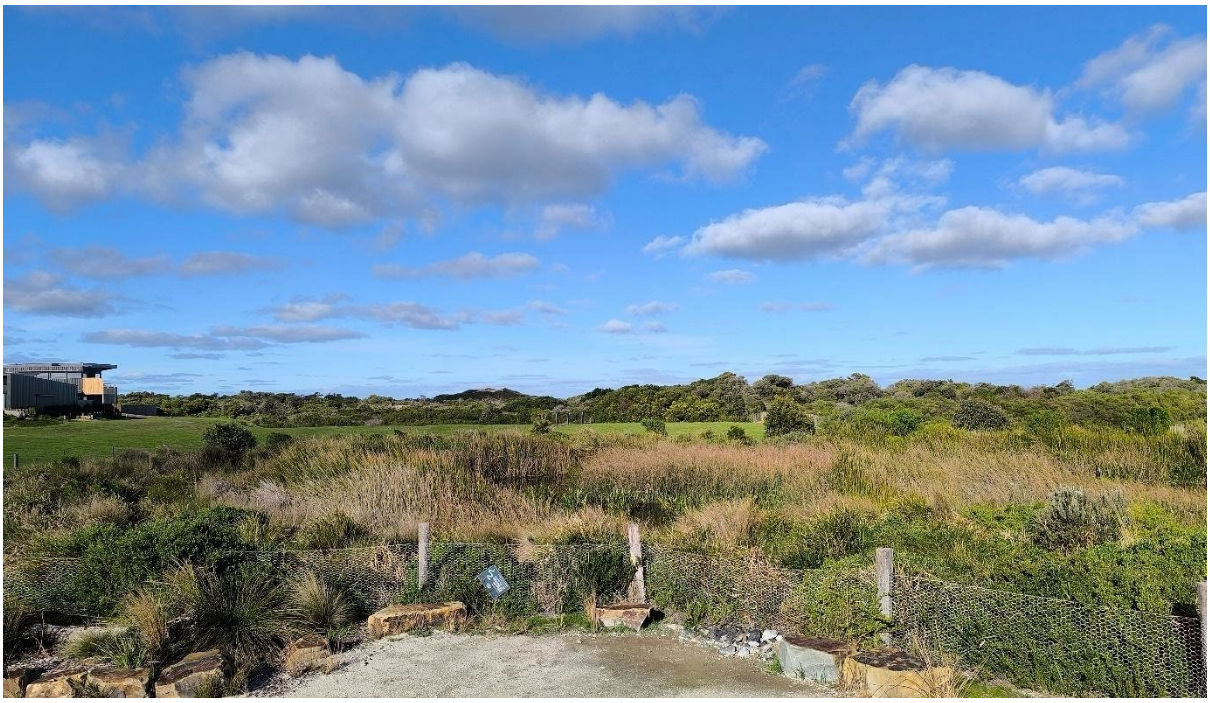

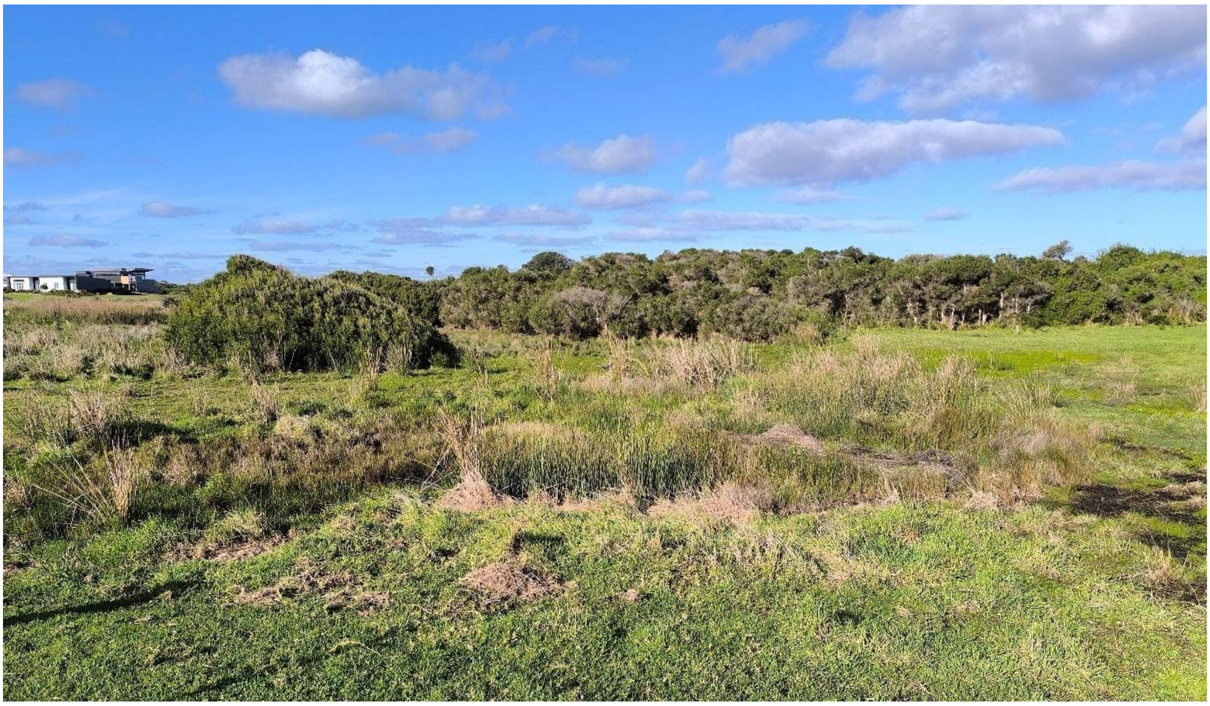

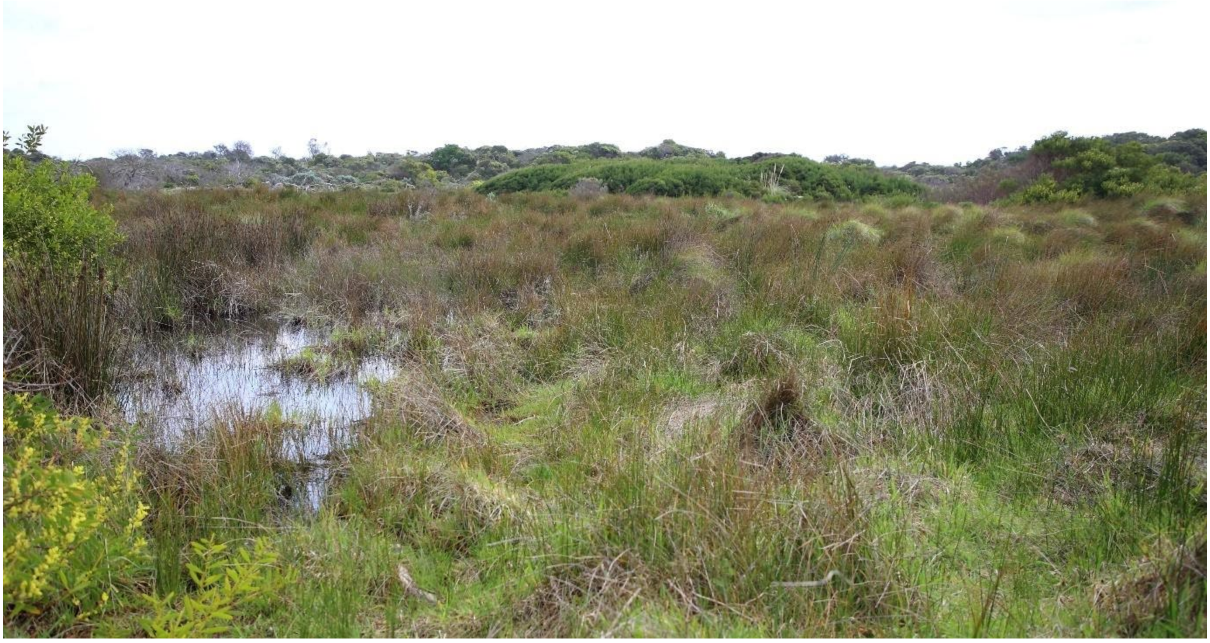

Cheltenham Road retarding basin, Victoria (-37.9907776, 145.1537213)

Artificial wetland containing unmown ryegrass, *Persicaria* sp., assorted weeds varying in height 0.3-0.65 m but often around 0.3-0.5 m in areas where snipe flush.

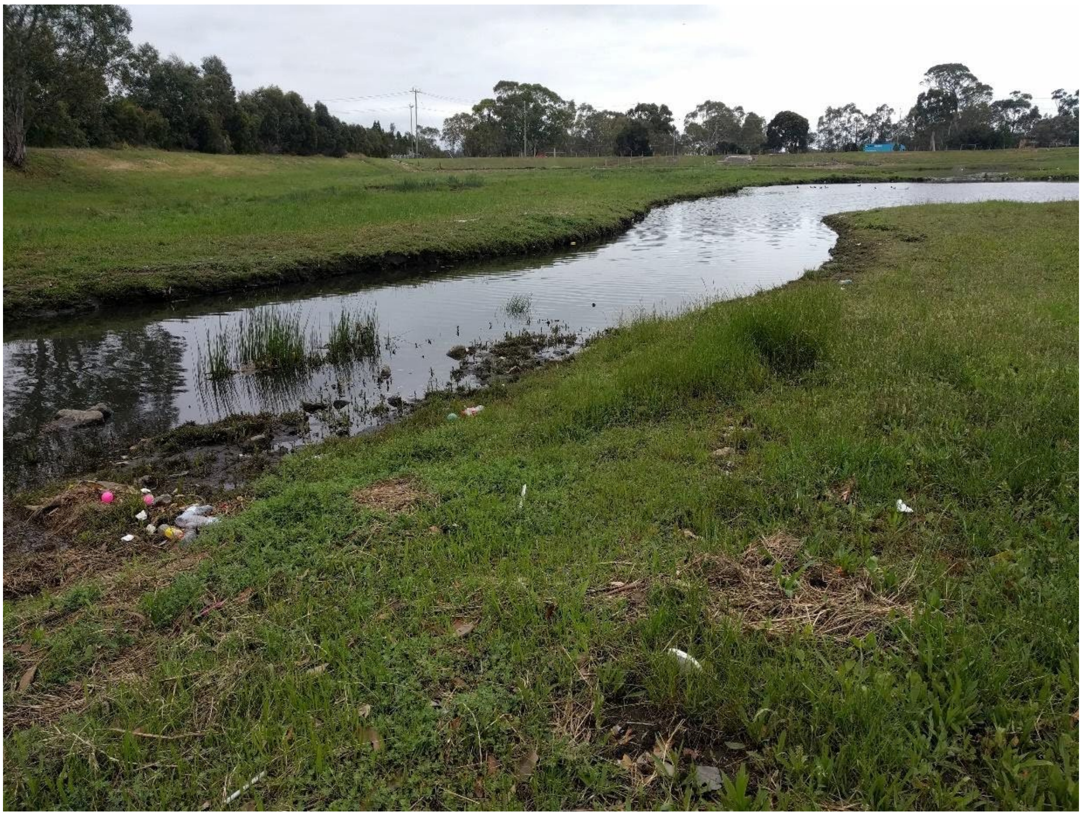

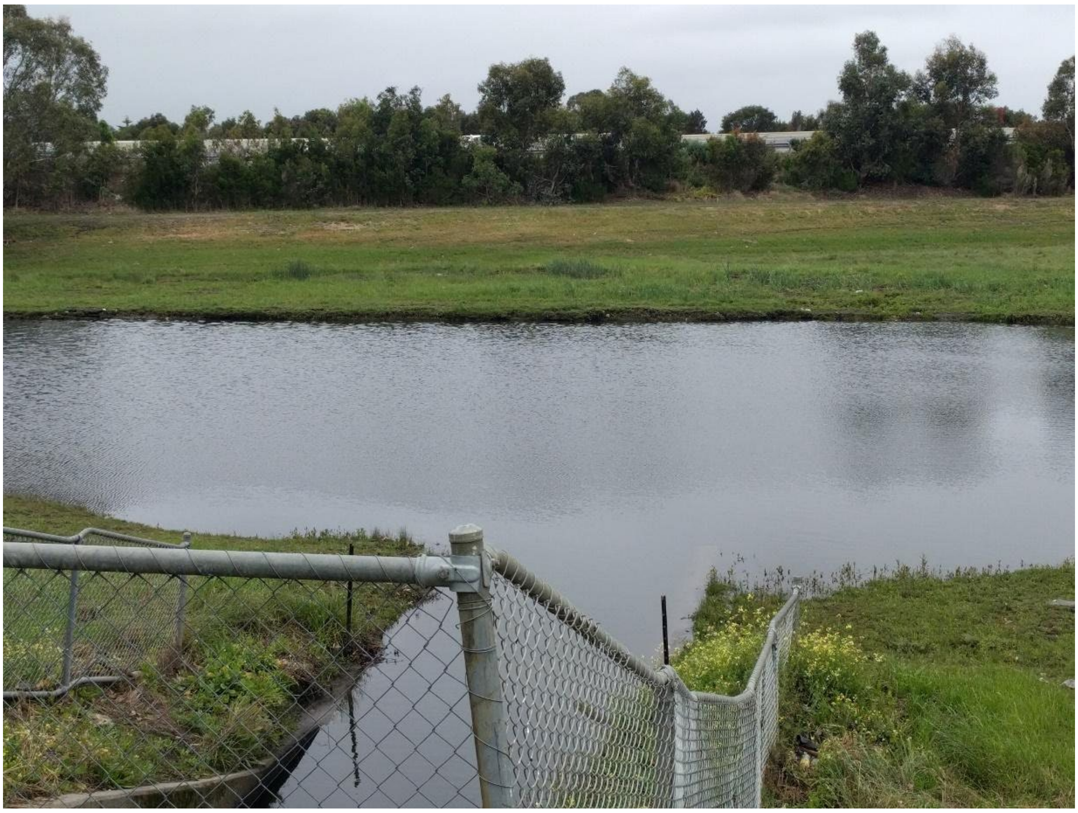

Clifton Creek swamp, Victoria (-37.7936755,147.6215332)

Tussocky, lightly cattle-grazed freshwater marshes containing *Juncus* sp., Triglochin, water couch, kikuyu, phragmites and *Poa* sp., typically around 0.1-1.0 m in height and up to 3 m in reedy areas. Photos courtesy of Louise Crisp.

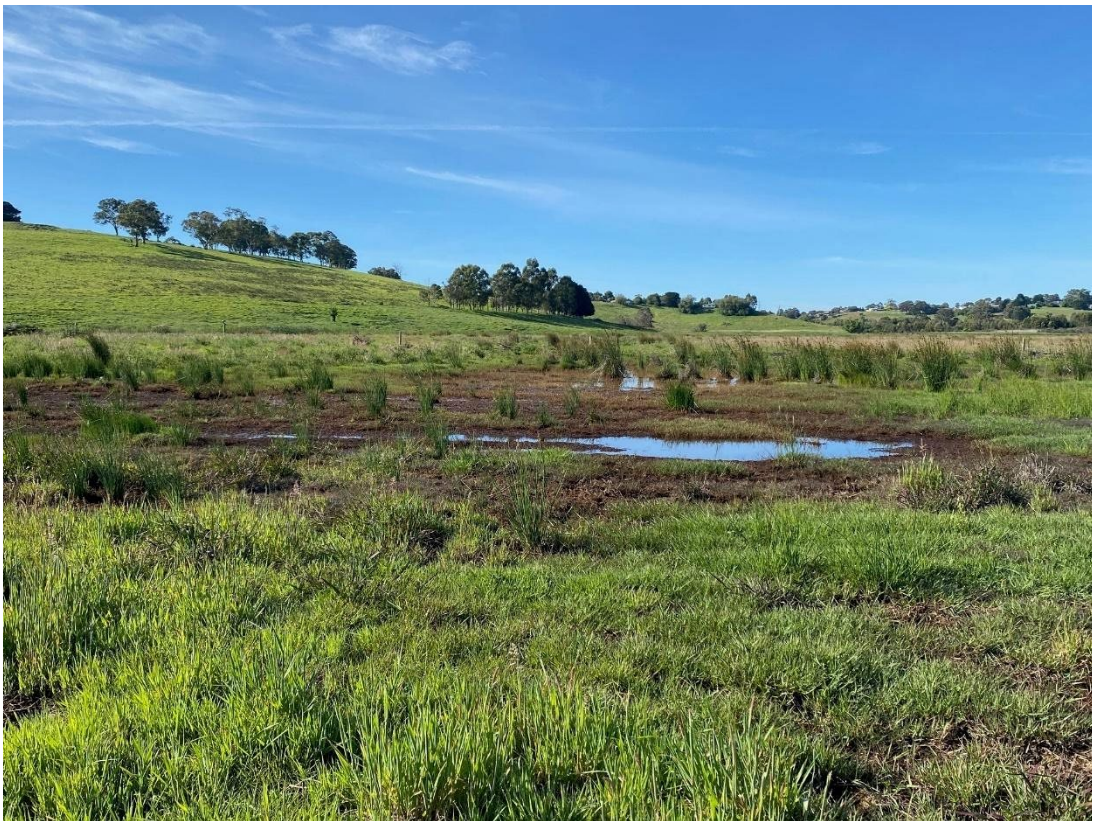

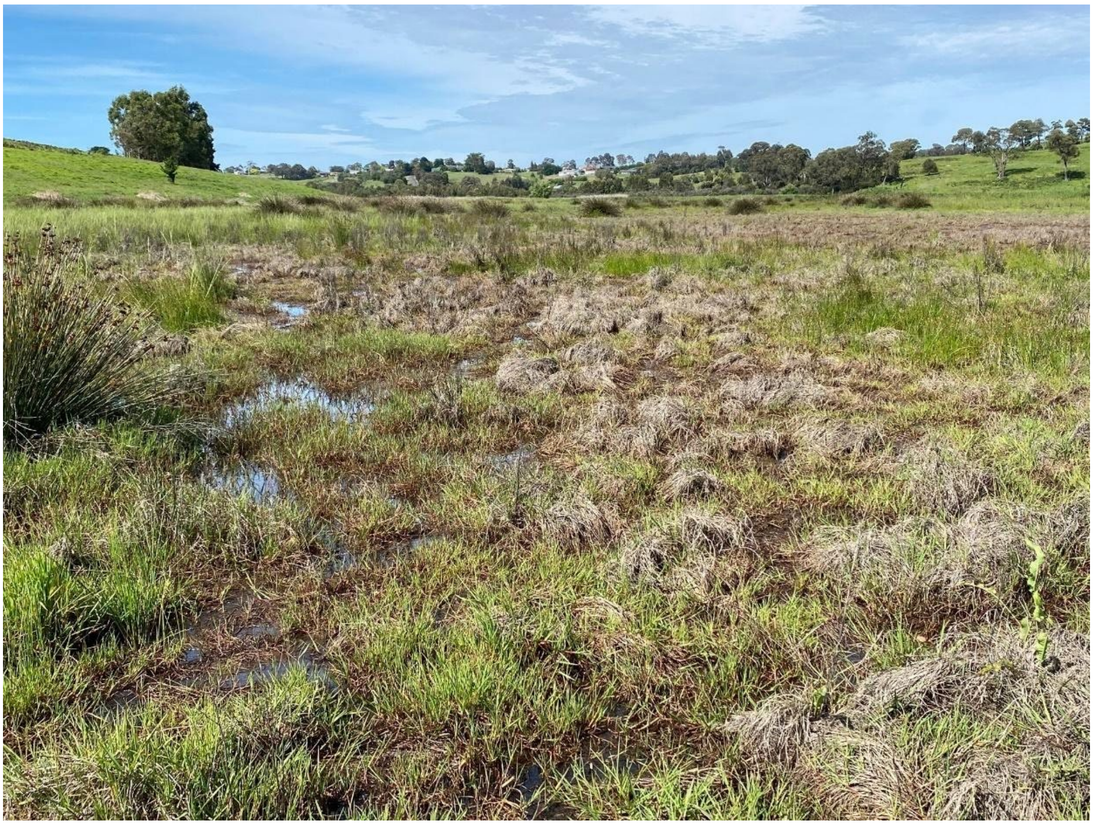

Fox and Pub Lakes, South Australia
(-37.1663072, 139.7777341; -37.1679026, 139.780412)

Estuarine wetland with saltmarsh typically 0.15 and up to 0.5 m high; fringing vegetation is *Melaleuca halmaturorum*. Photos courtesy of Sarah and Jeff Campbell, and Holly Prest.

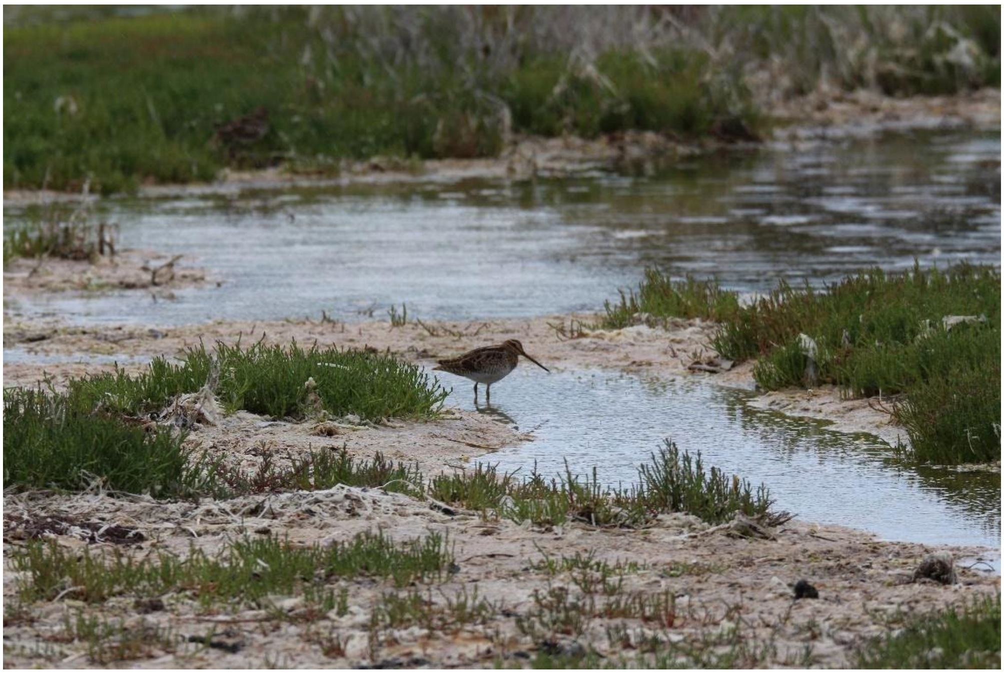

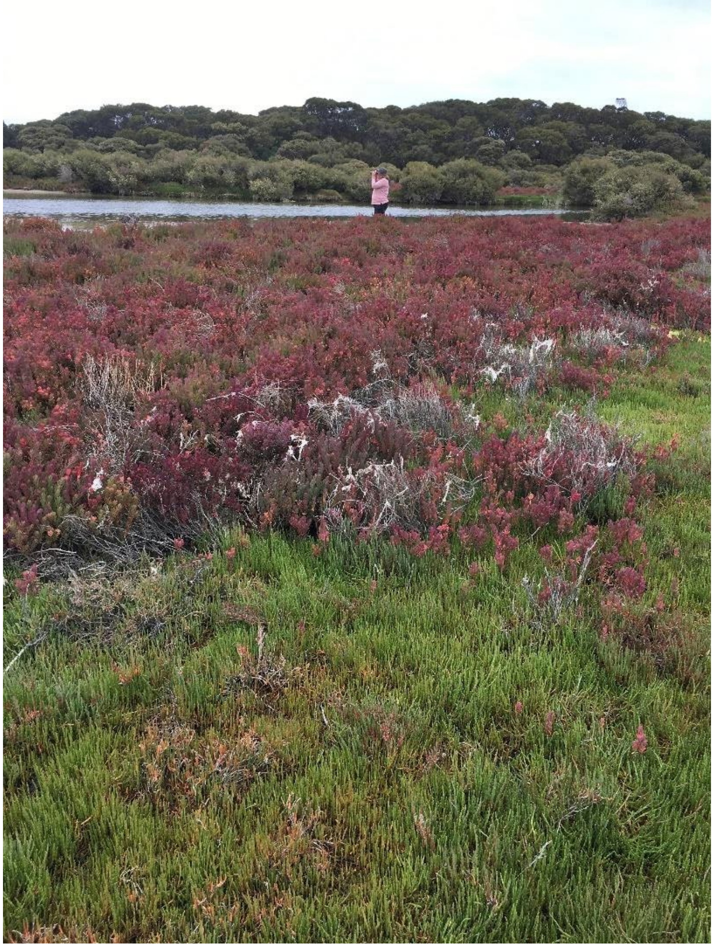

Heart Morass, Victoria

(-38.1388021, 147.162136; -38.1457894, 147.1233681; -38.1408233, 147.1020439)

Tussocky, freshwater marsh with vegetation height typically 0.2-0.5 m, connected to a larger floodplain system. Part of wetland used for duck hunting. Broader wetland characterised by inundated melaleuca and reed swamp periodically affected by saltwater inflows from Lake Wellington and Latrobe River estuary. Photos courtesy of Jack Winterbottom.

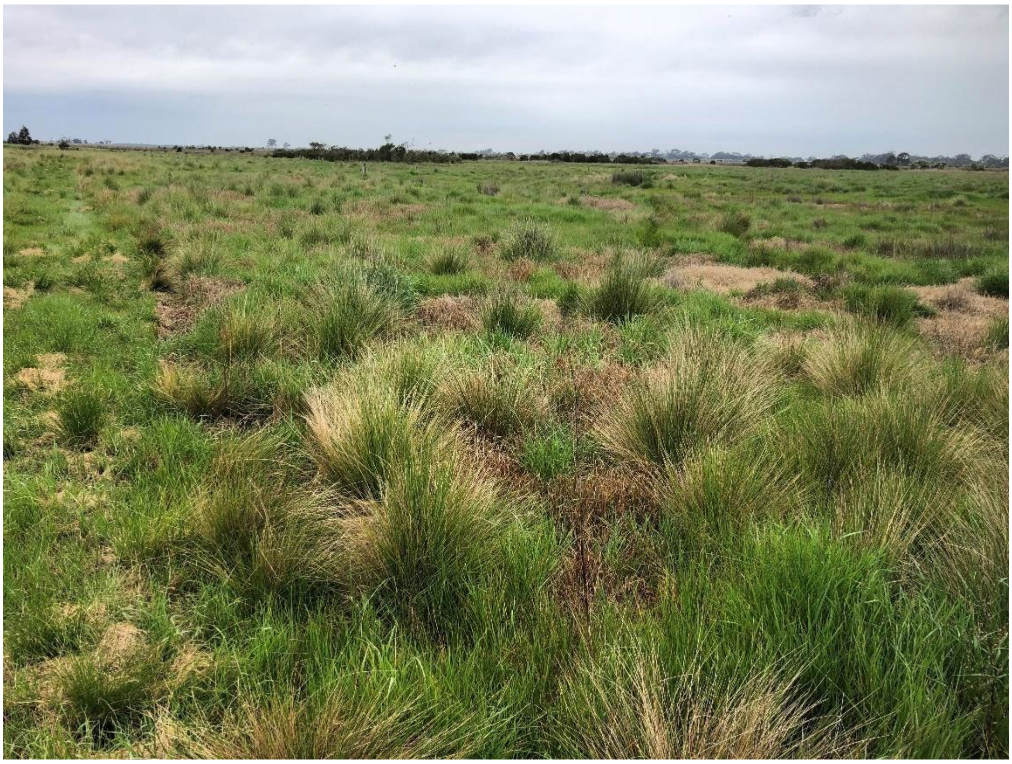

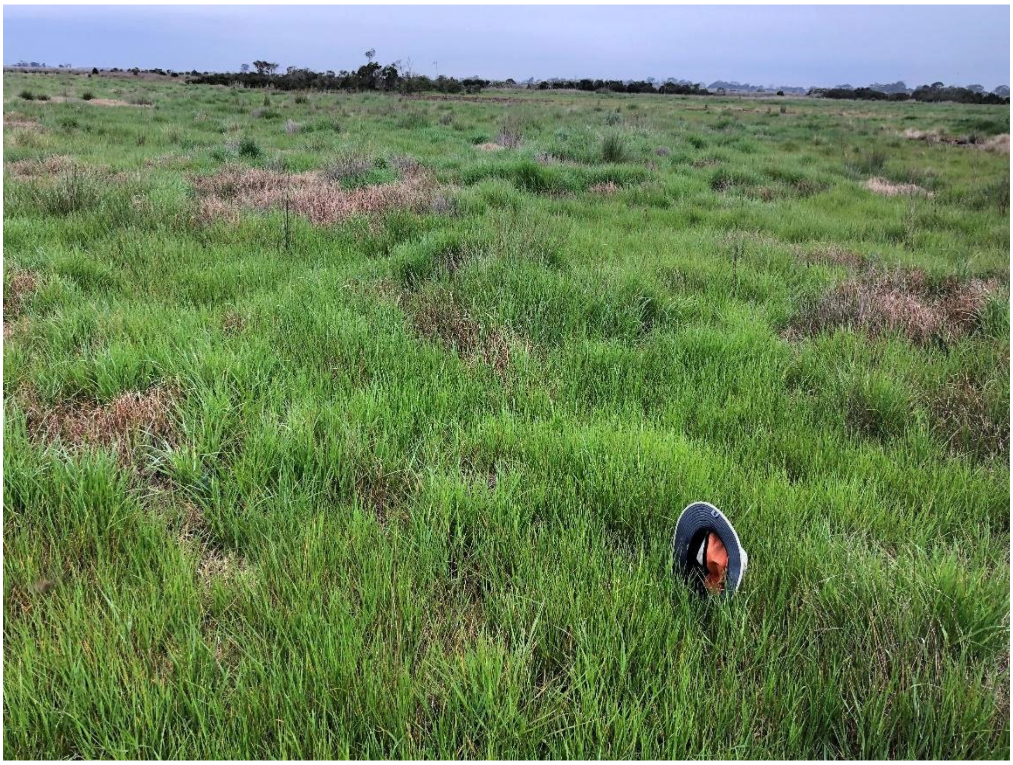

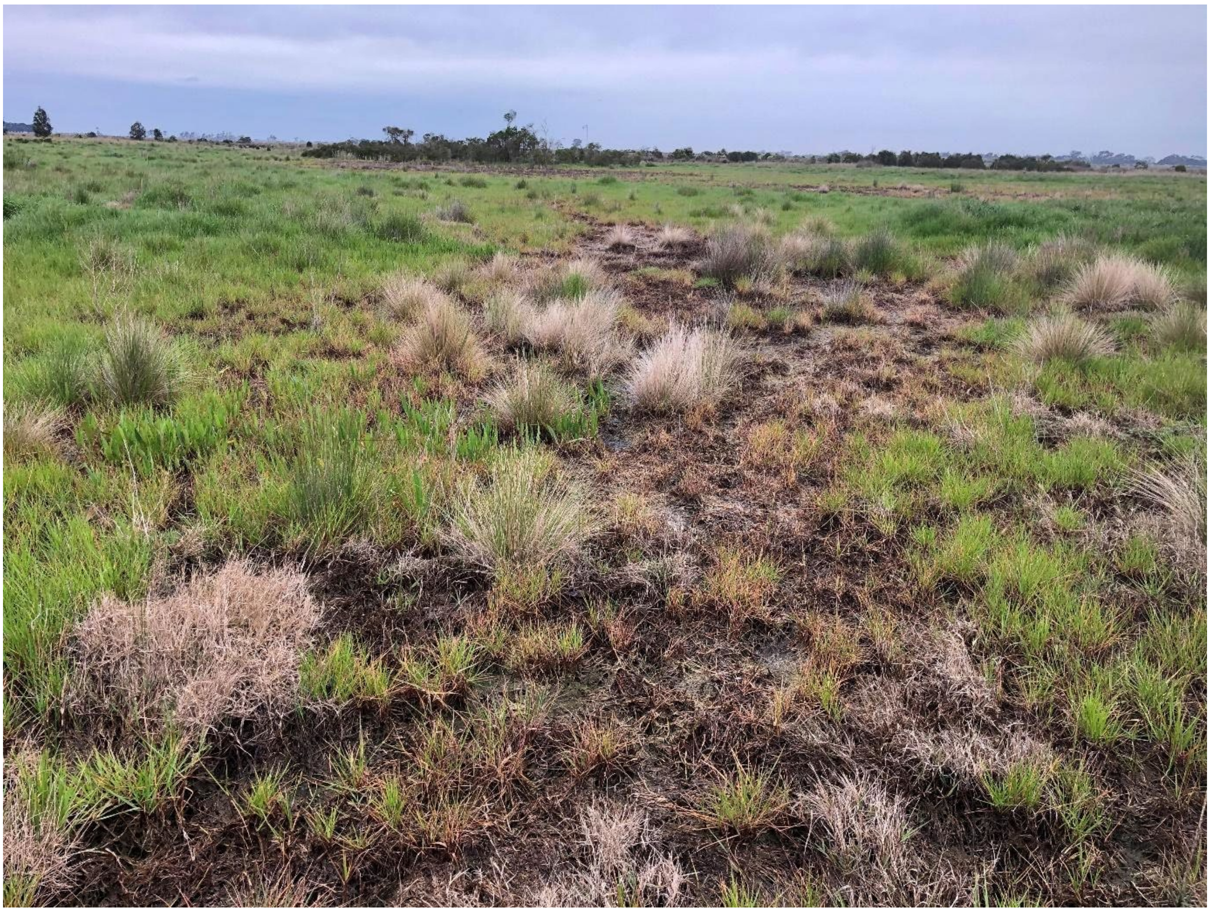

Melton Botanic Garden, Victoria (-37.6871645, 144.5955816)

Floodplain riparian wetland along a rehabilitated waterway (Ryan's creek) containing largely carex, poa, juncus tussocks and weeds (e.g. dock), between 0.2 to 1.5 m tall; Fringing vegetation Callistemon, Woolly Teatree, Eucalypts. Photos courtesy of John and Jill Bentley.

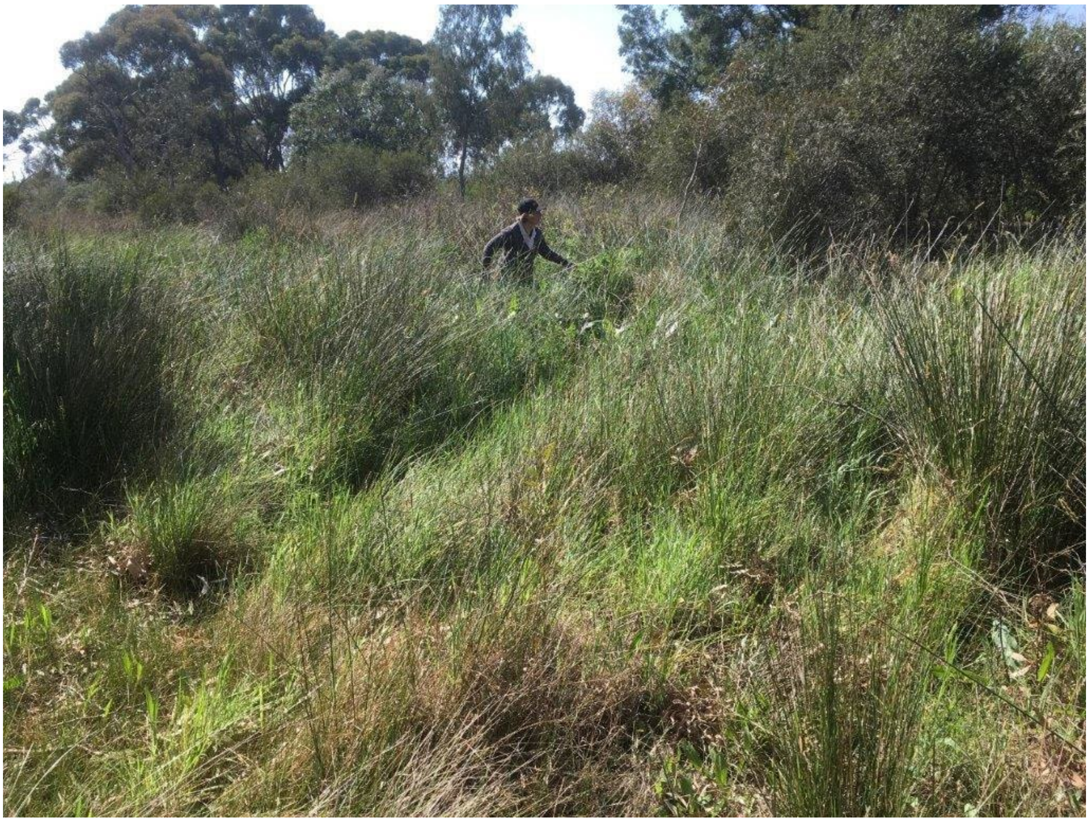

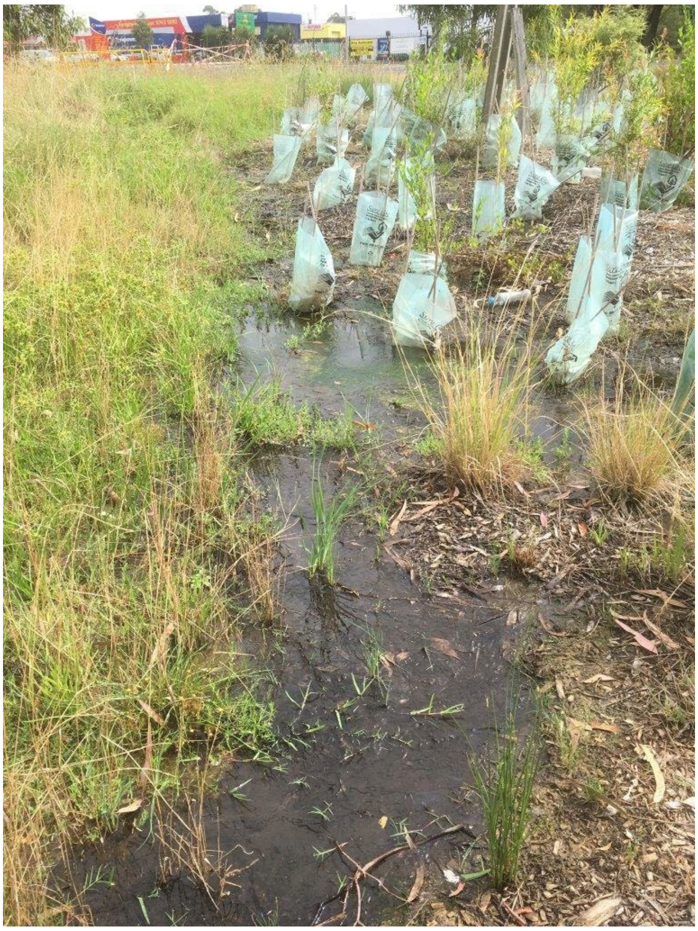

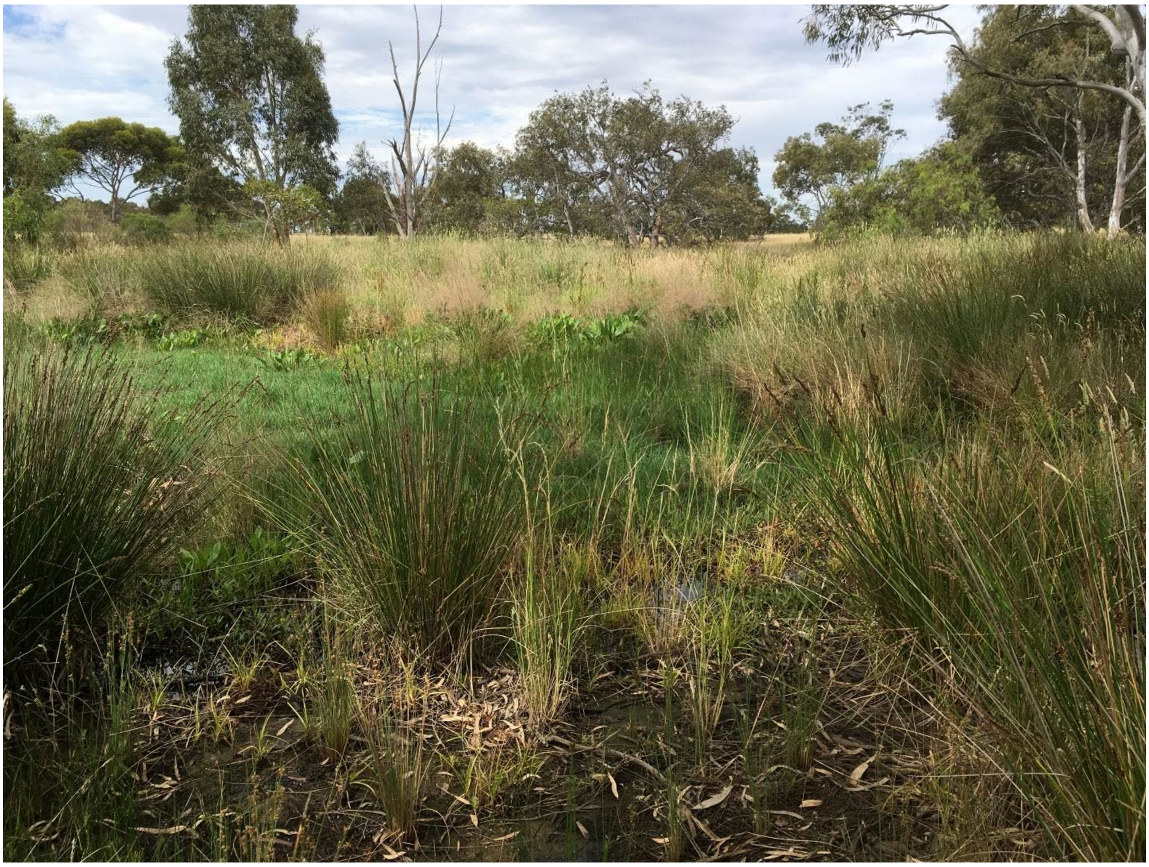

Moyne estuary, Victoria (-38.3786029, 142.2382533)

Partly modified, supra-tidal saltmarsh, typically 0.2-0.5 m, with scattered low shrubs. Intersected with lateral drains. Periodically inundated from riverine flooding. West bank vegetation typically lower in height than east bank, and largest abundance of snipe occur on the west bank.

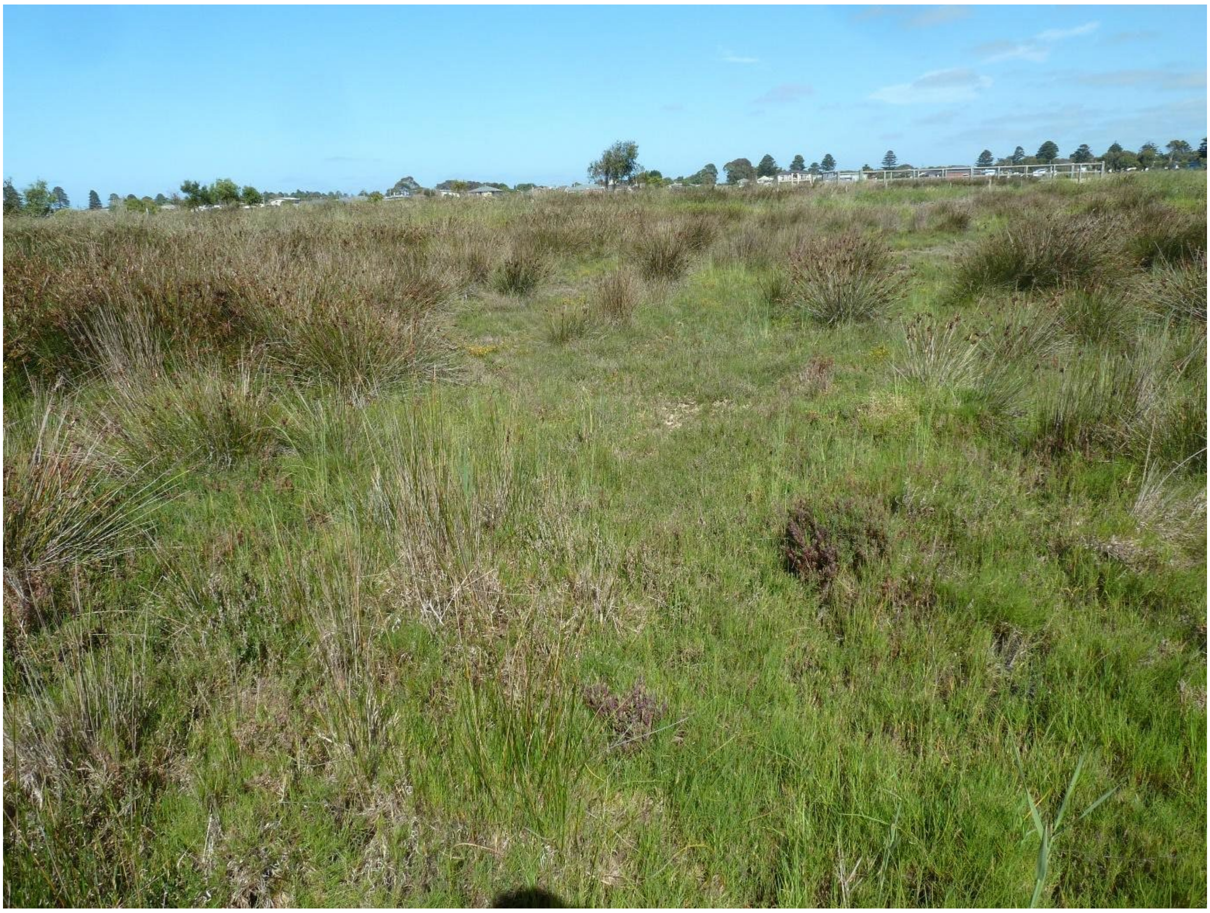

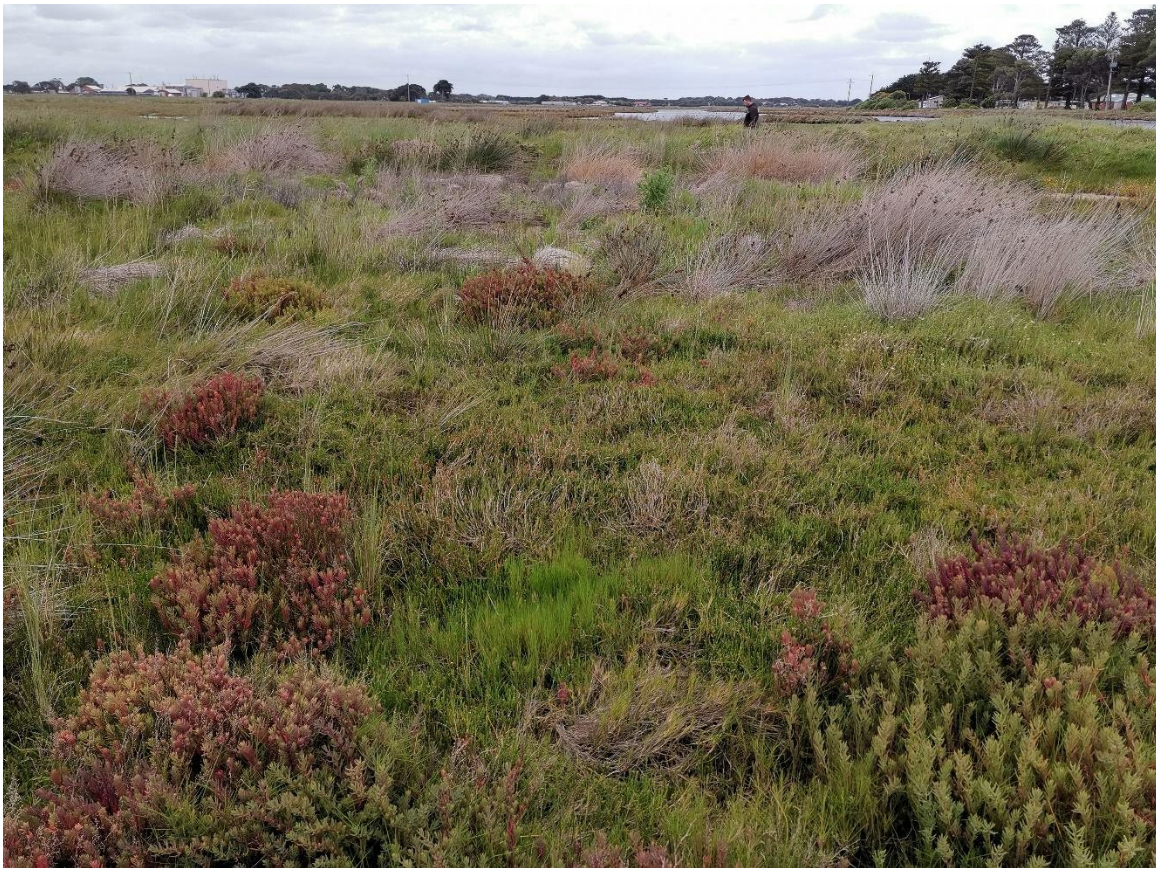

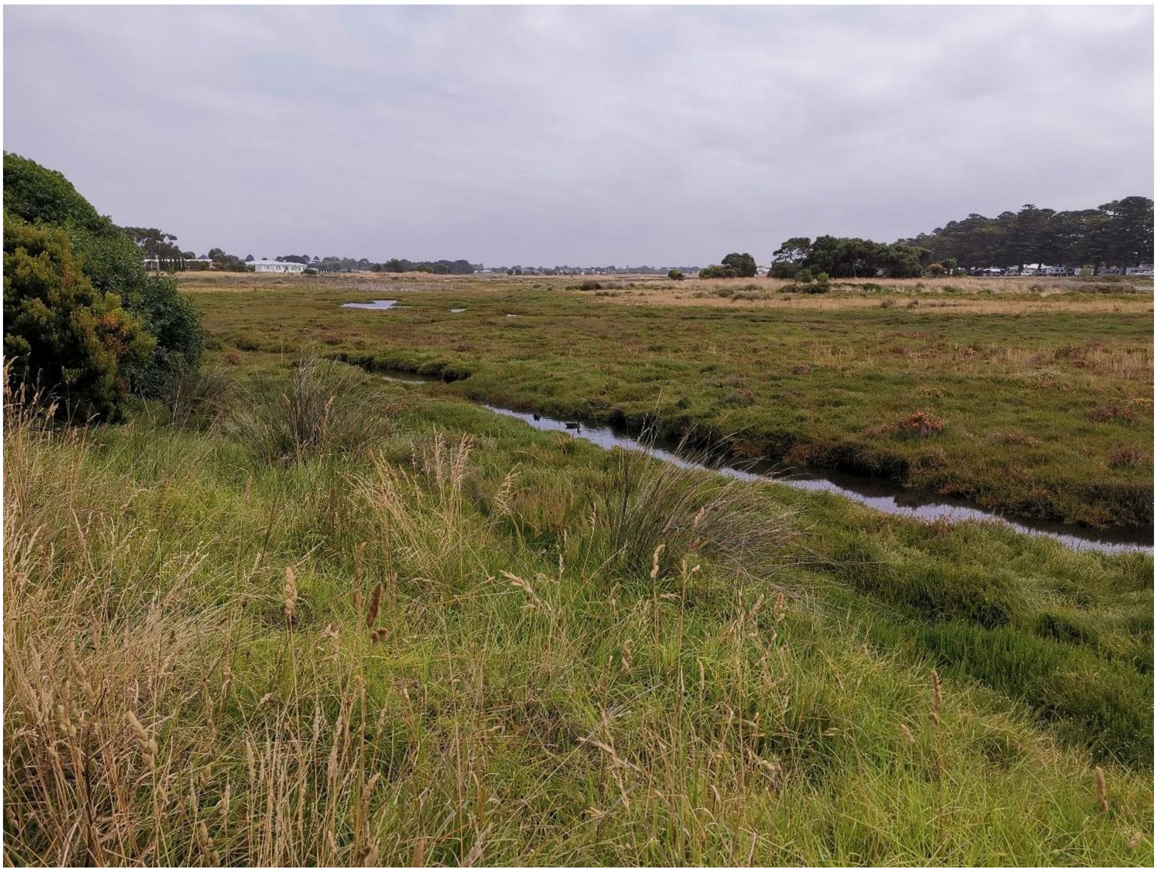

Peterborough wetlands, Victoria
(-38.6033558, 142.8731663; -38.6039427, 142.8740618; -38.60387, 142.8759124; -38.6033182, 142.8775476; -38.6026033, 142.8757854; -38.6025431, 142.87411; -38.606283, 142.8774802; -38.6061287, 142.8741677; -38.6053687, 142.8736911; -38.6044394, 142.8724634; -38.6011036, 142.876116)

Tussocky shallow freshwater swamps, fringing areas contain grassy / weedy pasture 0.2-0.5 m high. Area contains 11 waterbodies with five being regularly used by snipe, some abutting residential land and regularly mowed.

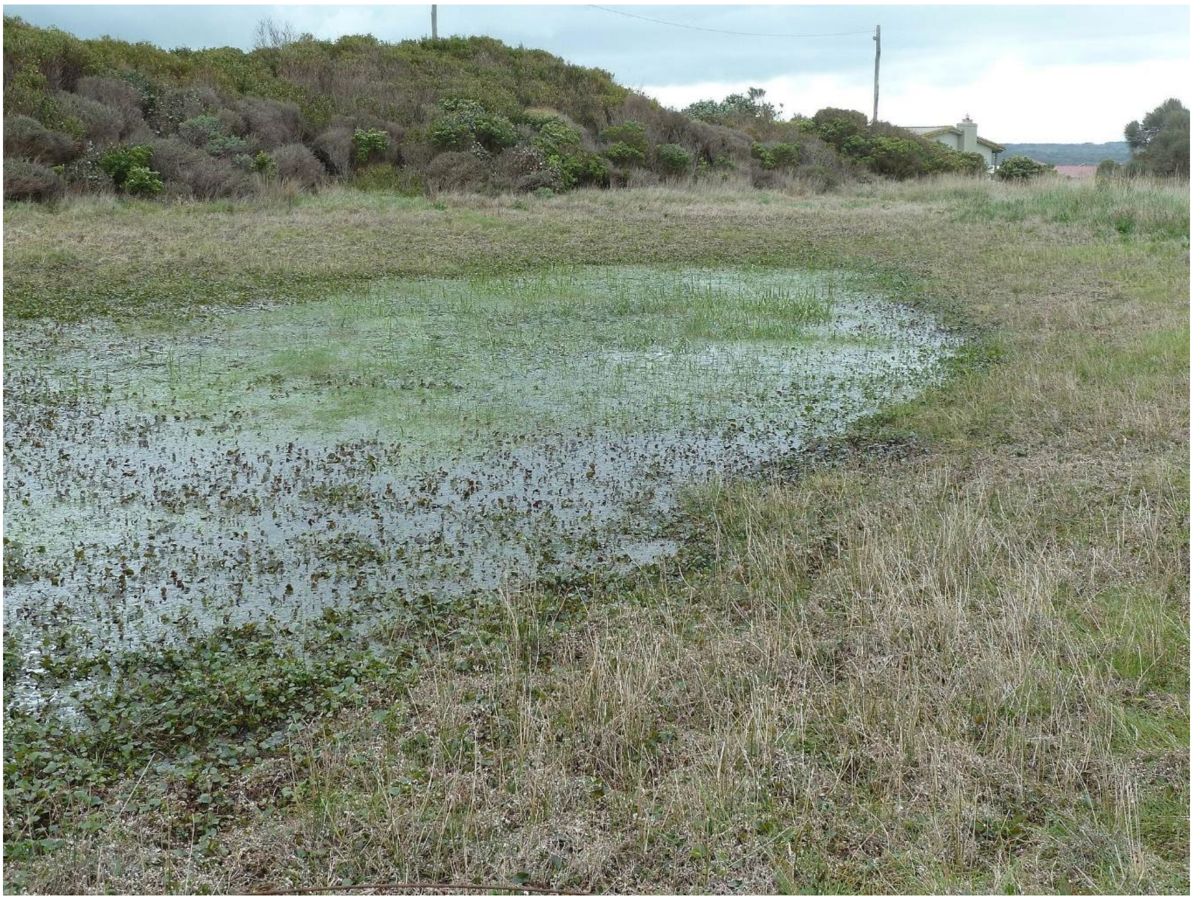

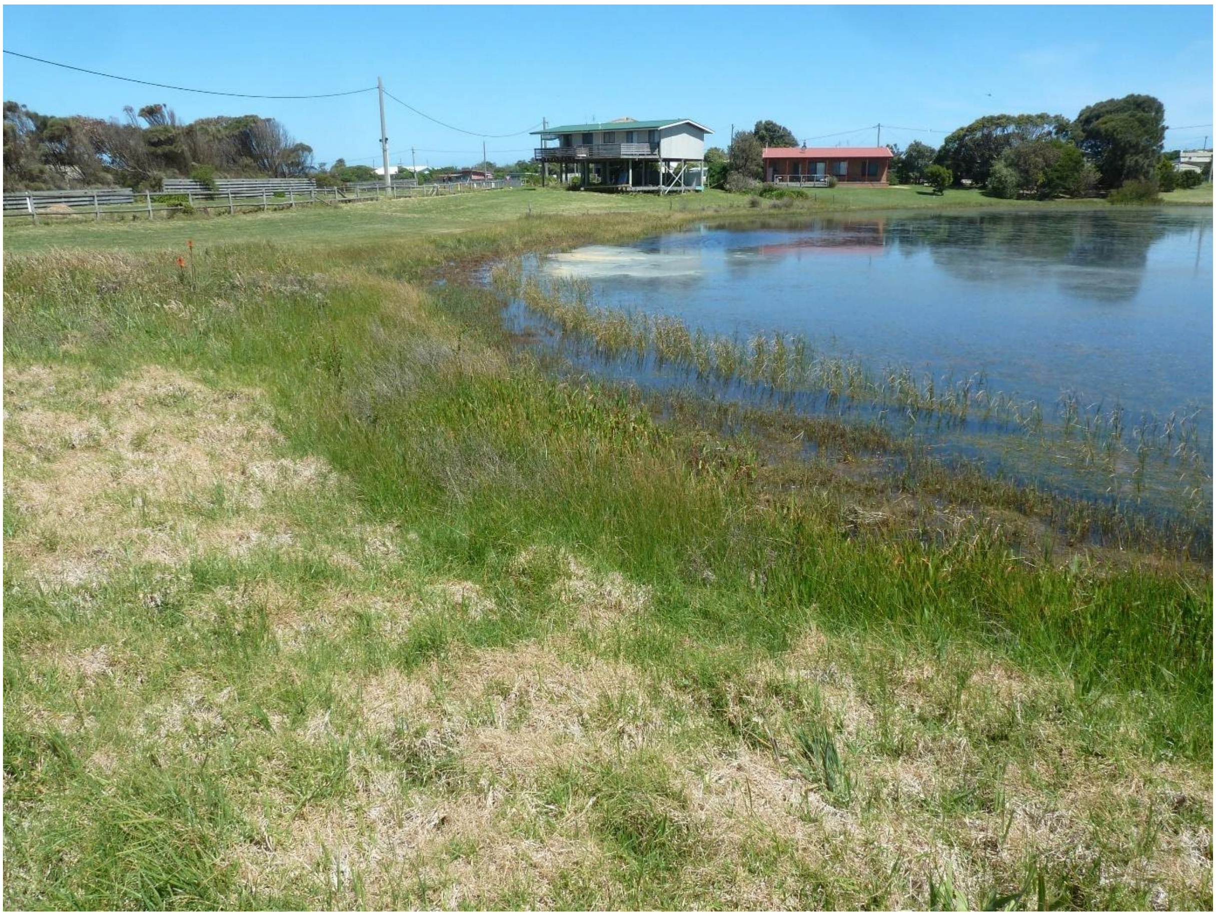

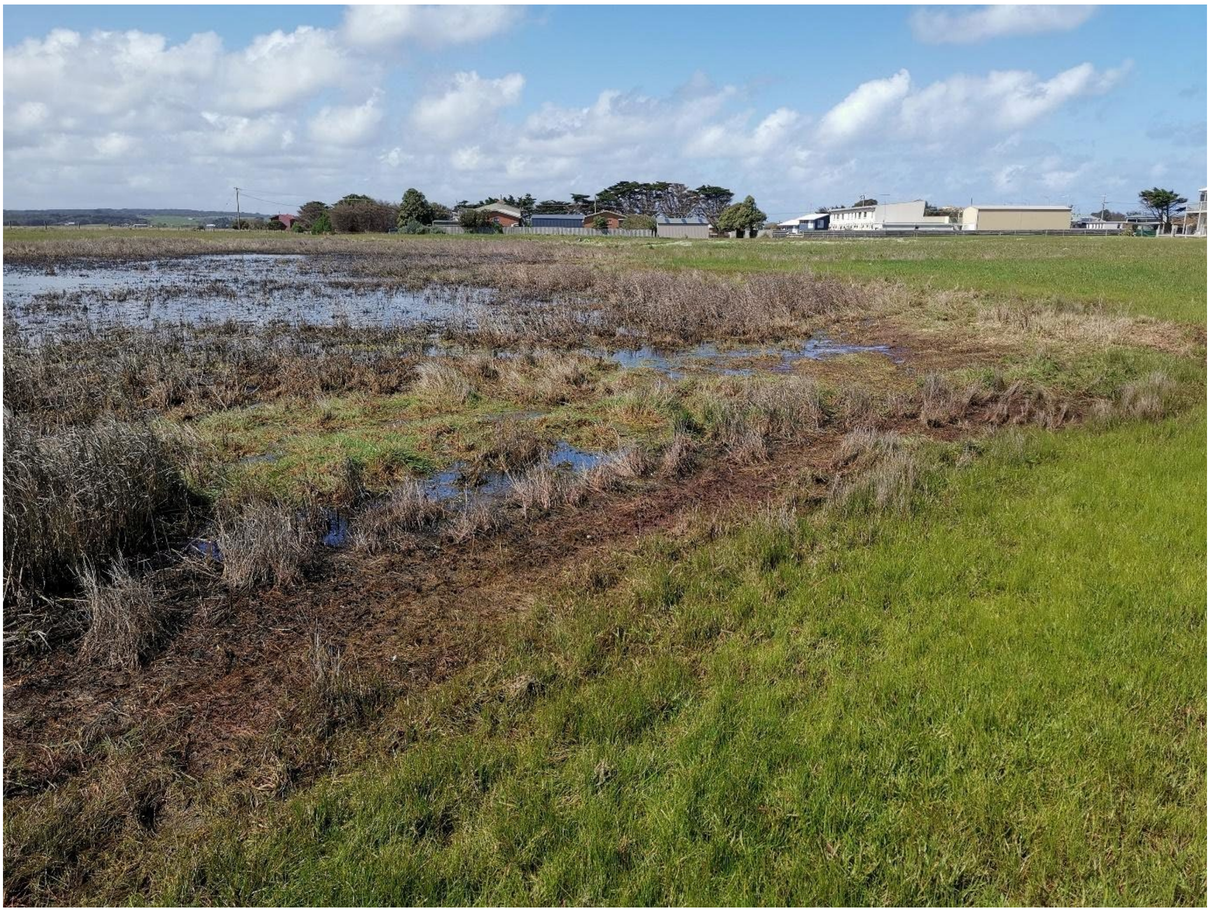

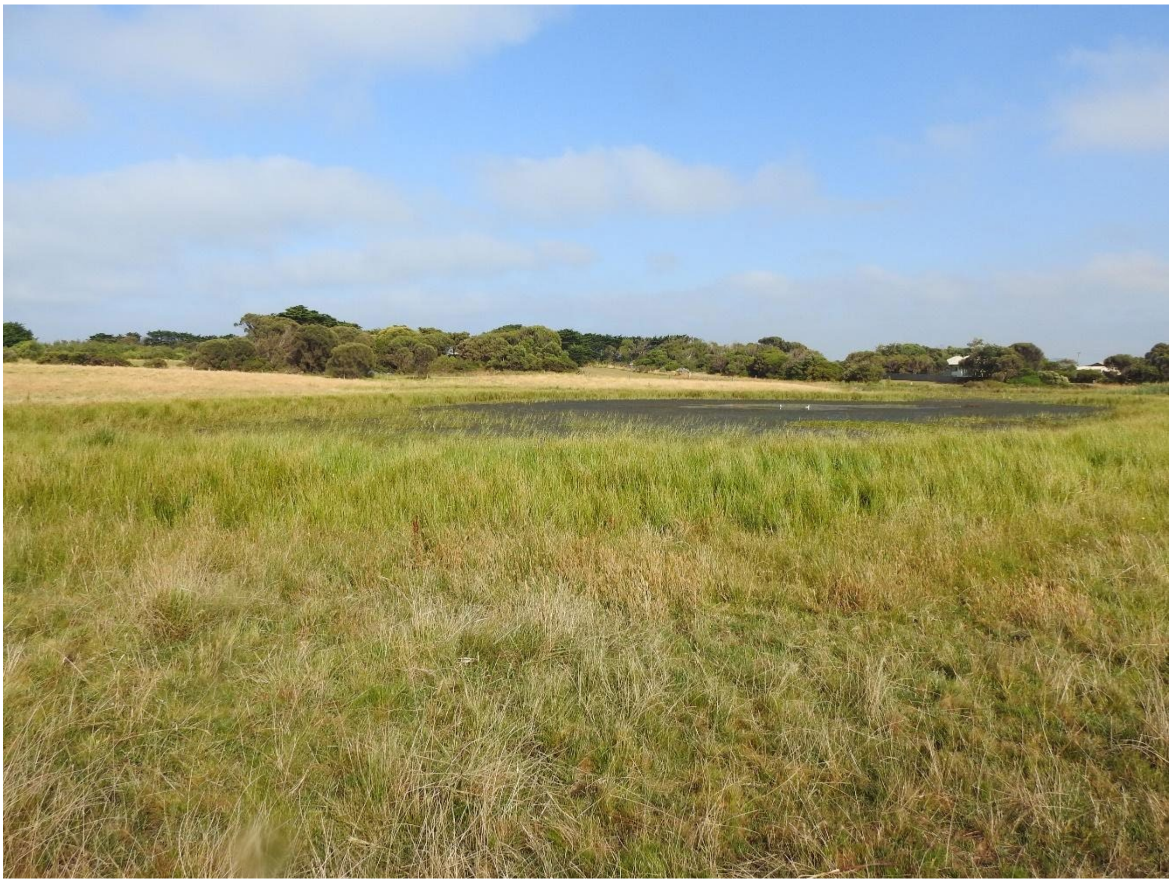

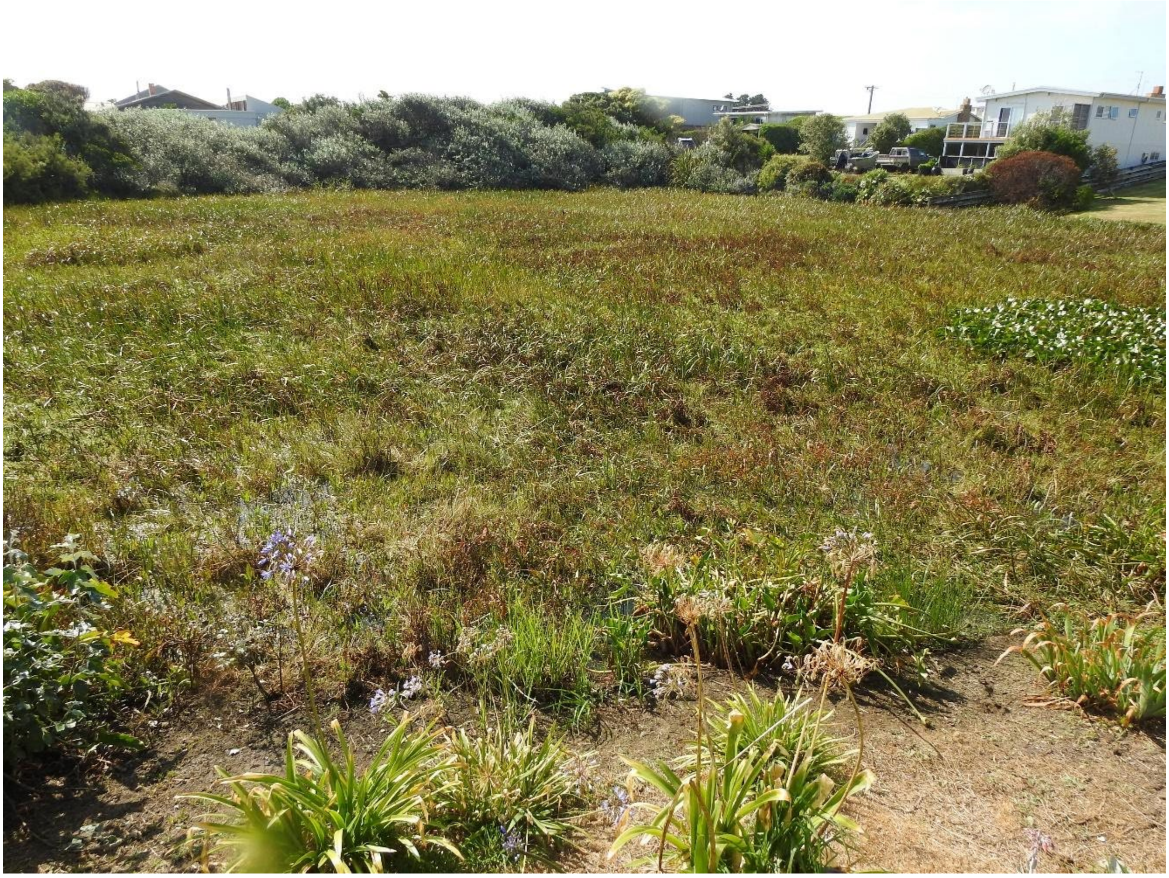

Powling Street wetlands, Victoria
(-38.3876688, 142.2245137; -38.3896895, 142.2289166)

Kikuyu and reed freshwater modified swamp 0.5-0.6 m, with fringing Leucopogon shrubs and weedy grassland. Separate weedy / stony grassland area (“Department of Education land”) seasonally soaked and now geographically separated from the main wetland by housing development.

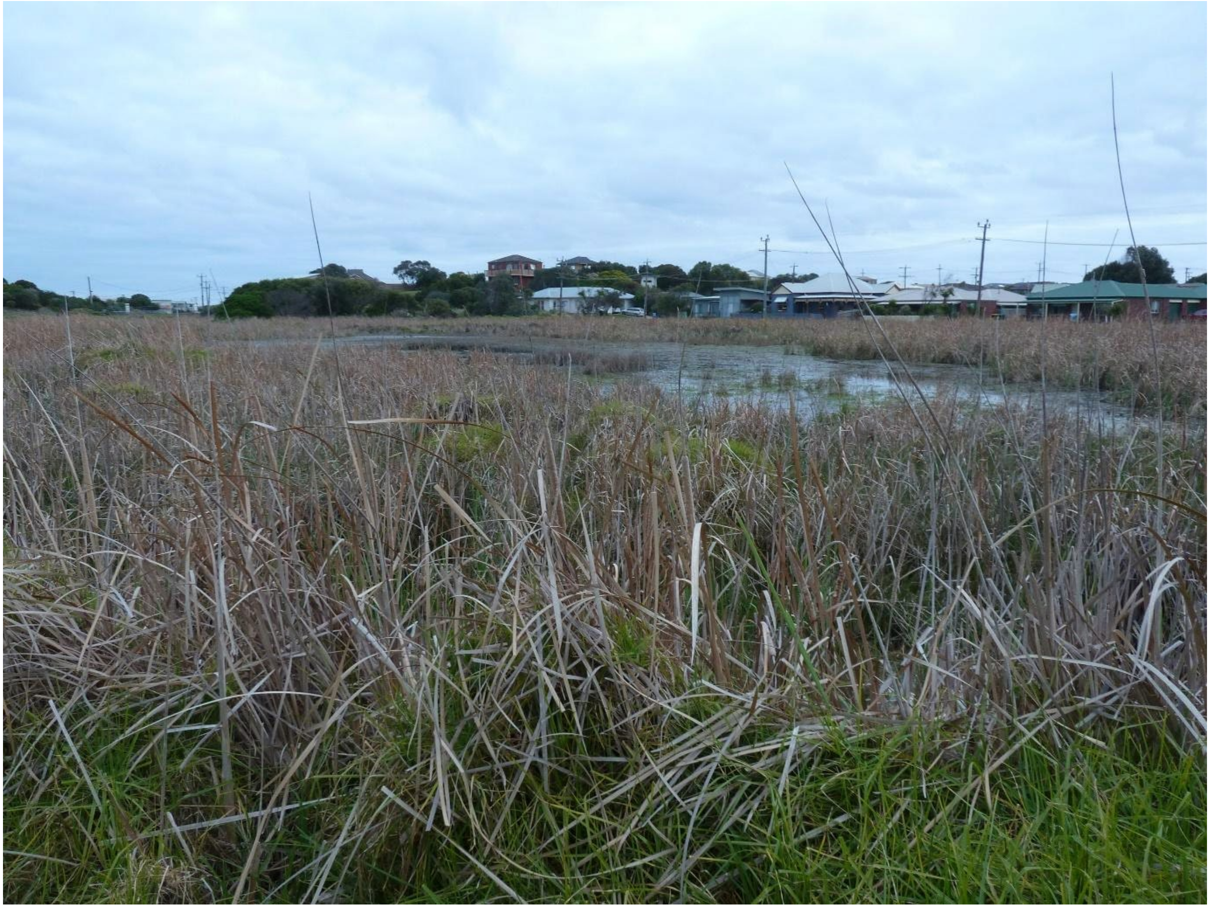

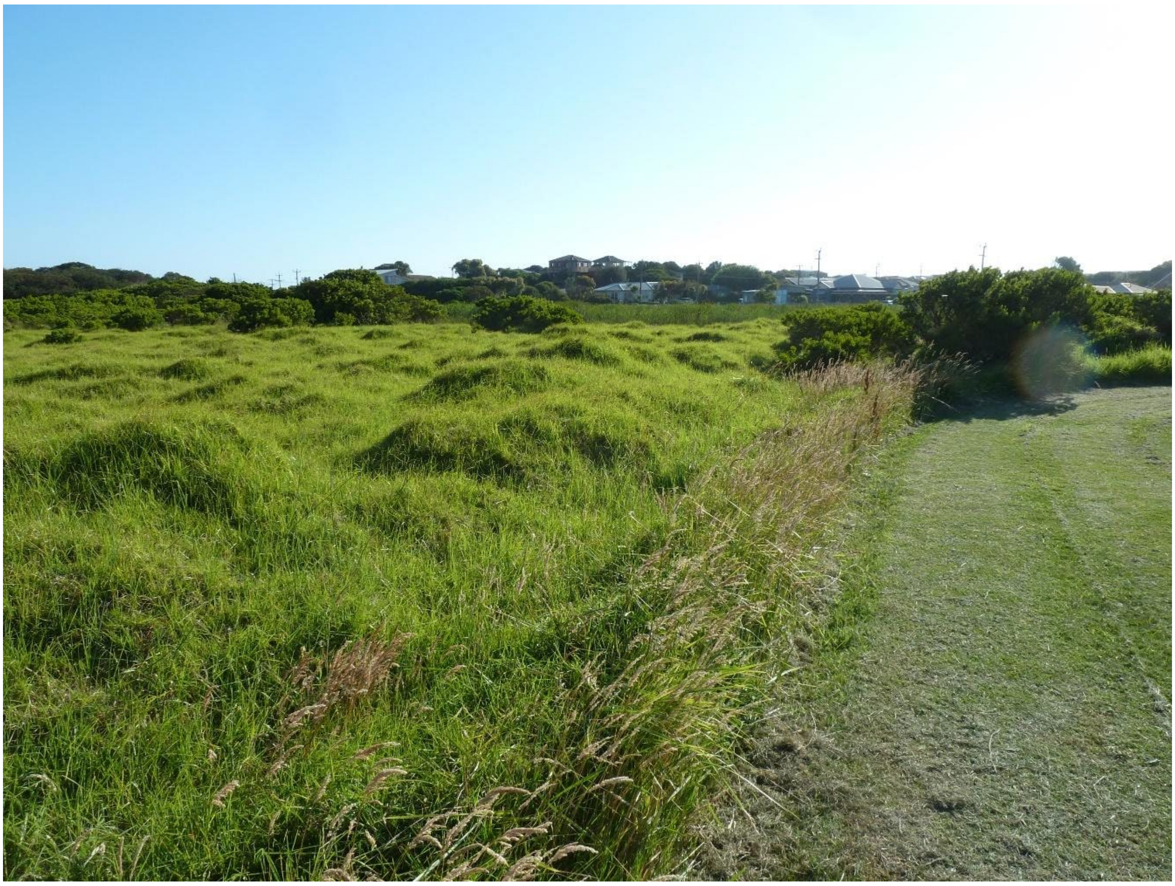

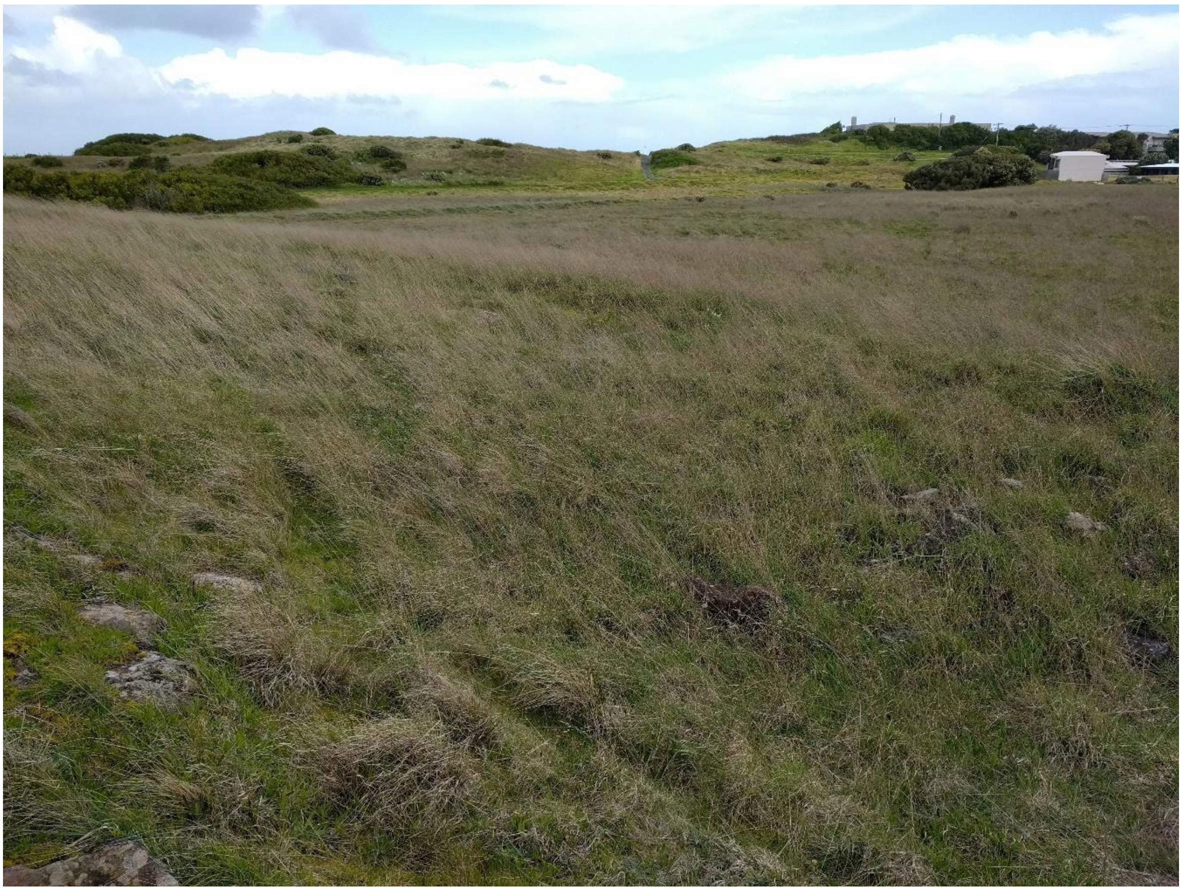

Railway Place wetland, Victoria (-38.3799766, 142.2372065)

Tussocky freshwater retention pond alongside Port Fairy rail trail and adjacent to Moyne estuary. Vegetation heights vary from tall, thick Juncus tussocks at drier south end up to 1.5 m to low weed / tussock swampy areas 0.3-0.6 m.

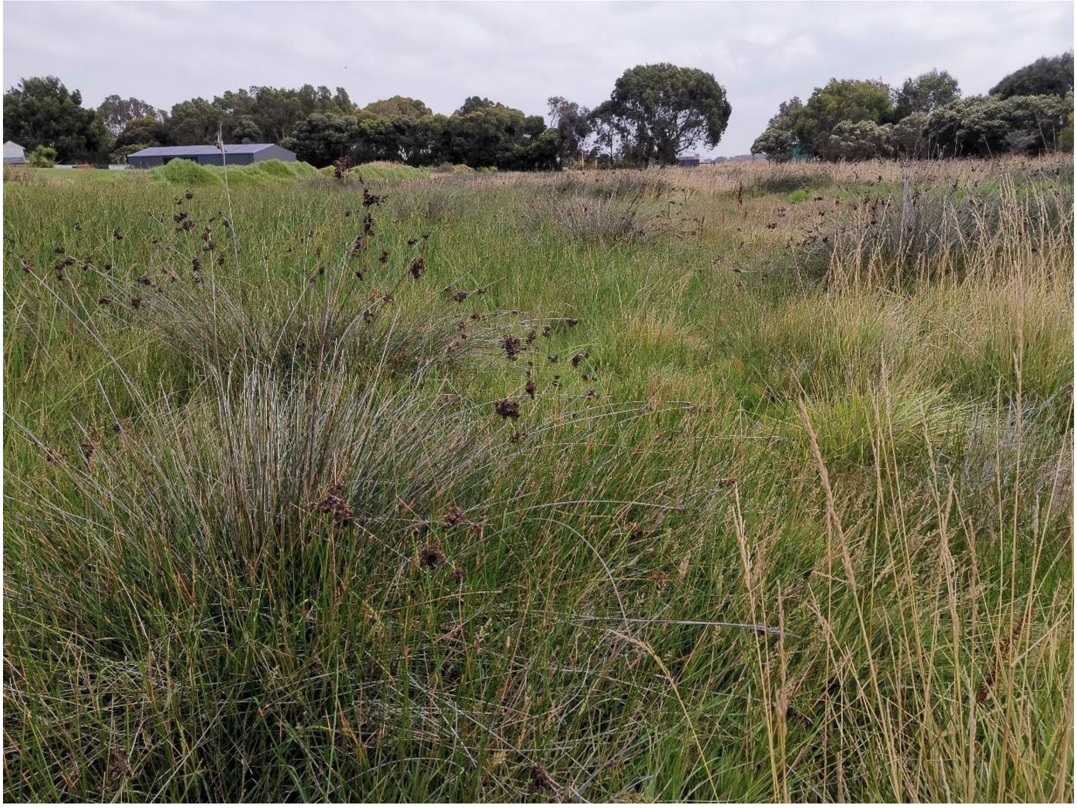

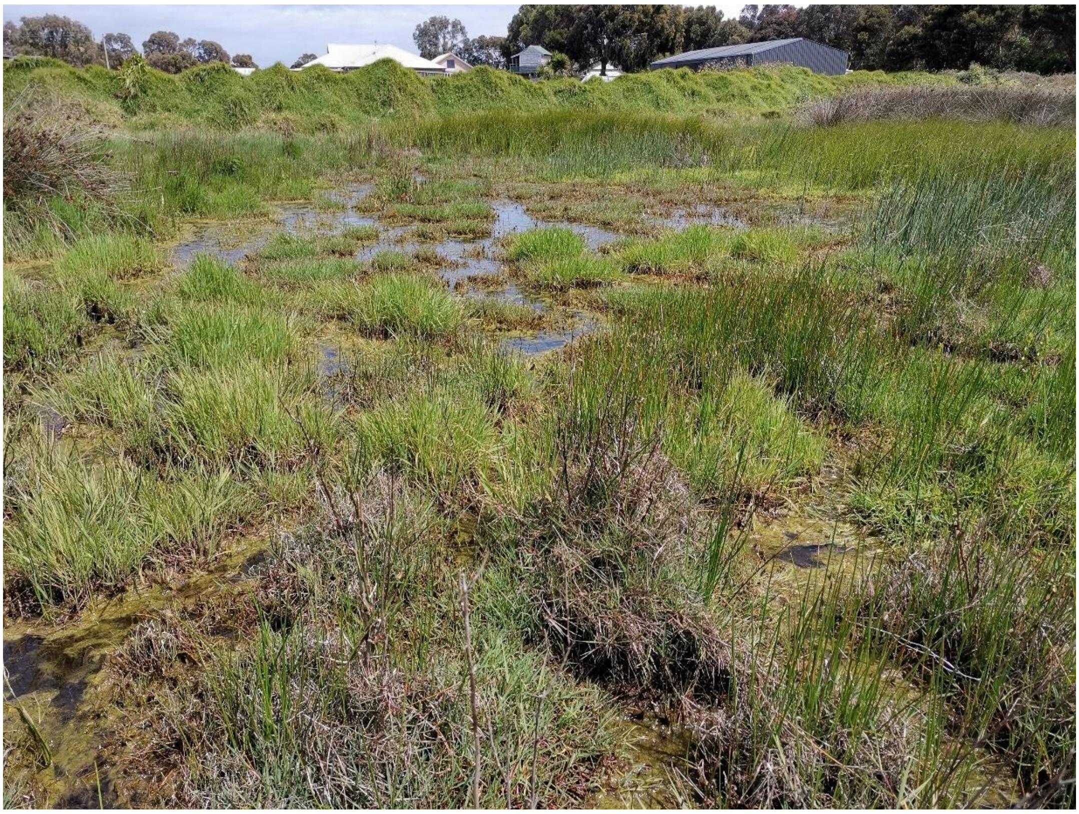

Retarding Basins Dandenong Valley Hwy, Victoria
(-38.0324703, 145.2245981; -38.0364222, 145.2128909; -38.0371228, 145.2097713)

Freshwater roadside retention basins with grass (often mowed) and Juncus / Poa tussocks, Baumea, Bulboschoenus, and Typha reeds with fringing swamp paperbark. Vegetation height varies between 0.1-0.5 m.

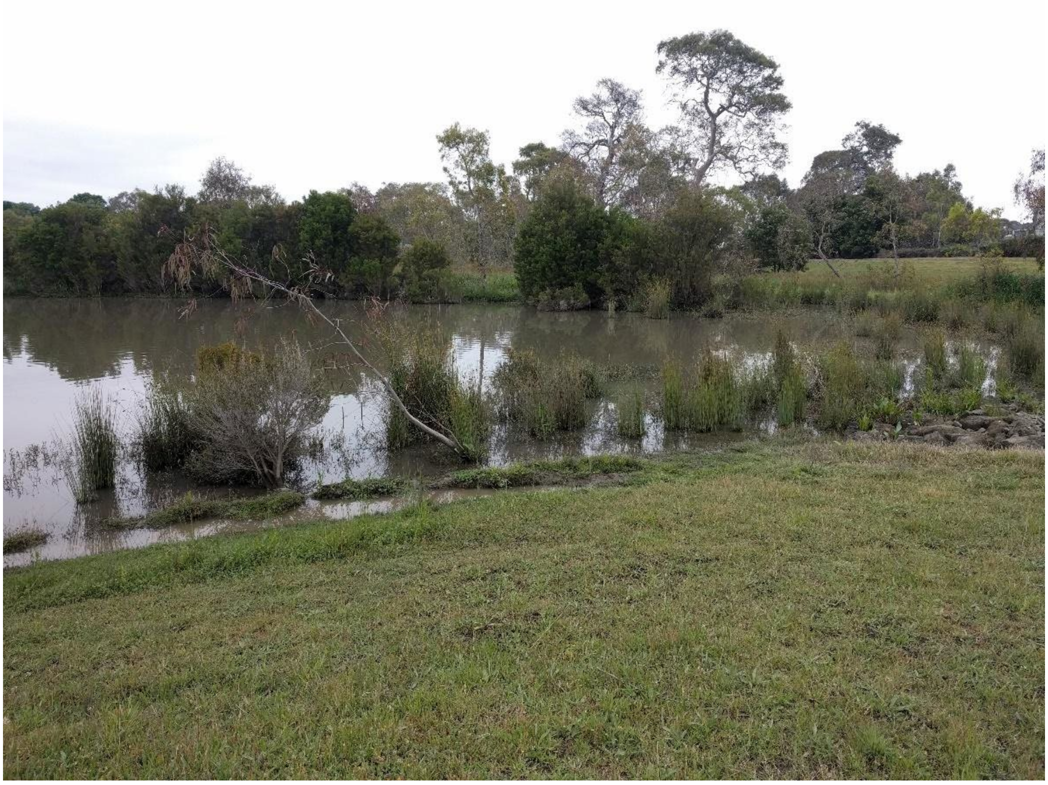

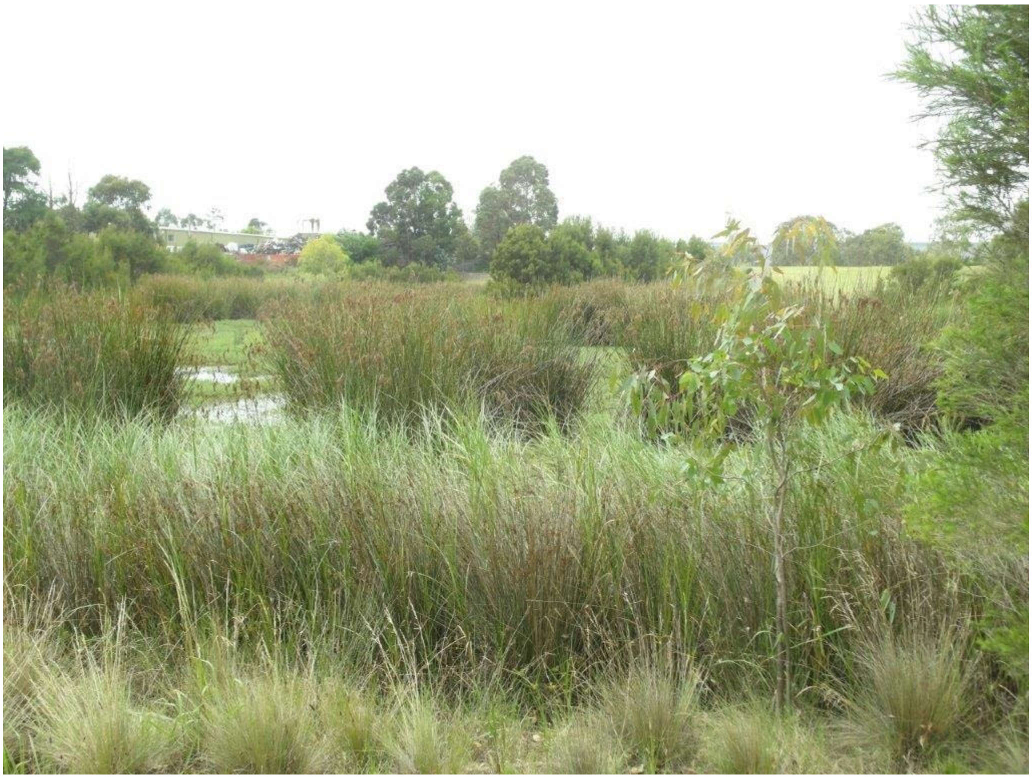

Saltwater Creek, Victoria (-38.457629, 145.1996535)

Riparian weedy / grassy and Juncus / sedge tussock floodplain wetlands with some saltmarsh, 0.1-0.8 m high; fringing vegetation scattered planted trees, Boobialla and Melaleuca. Adjacent to a golf course. Photos courtesy of Jon Fallaw, Roz Jessop and Sharon Woodend.

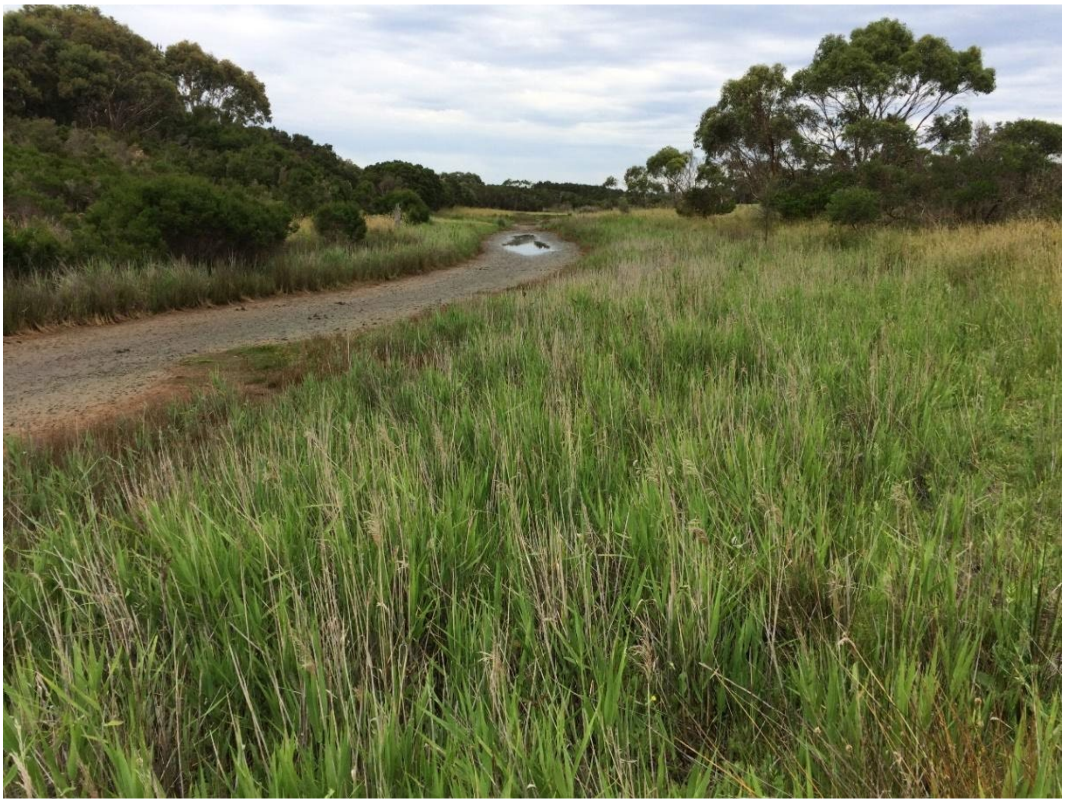

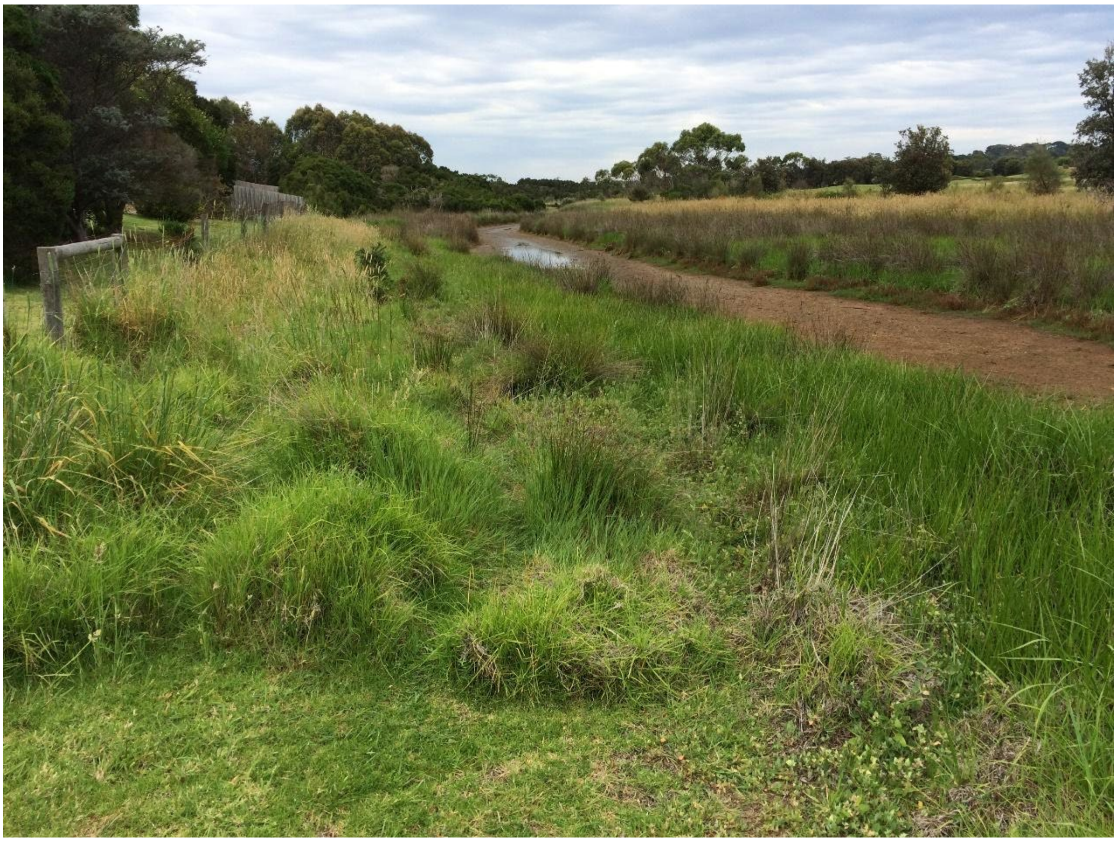

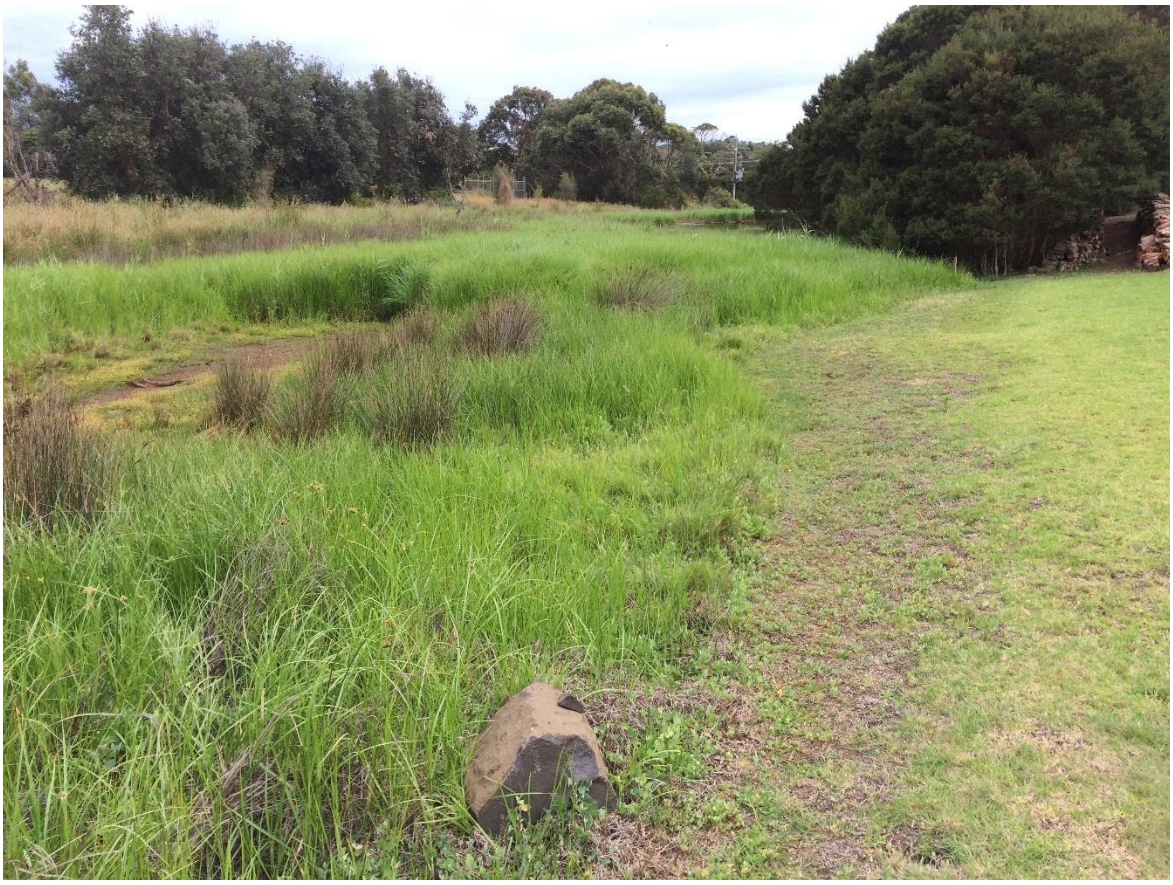

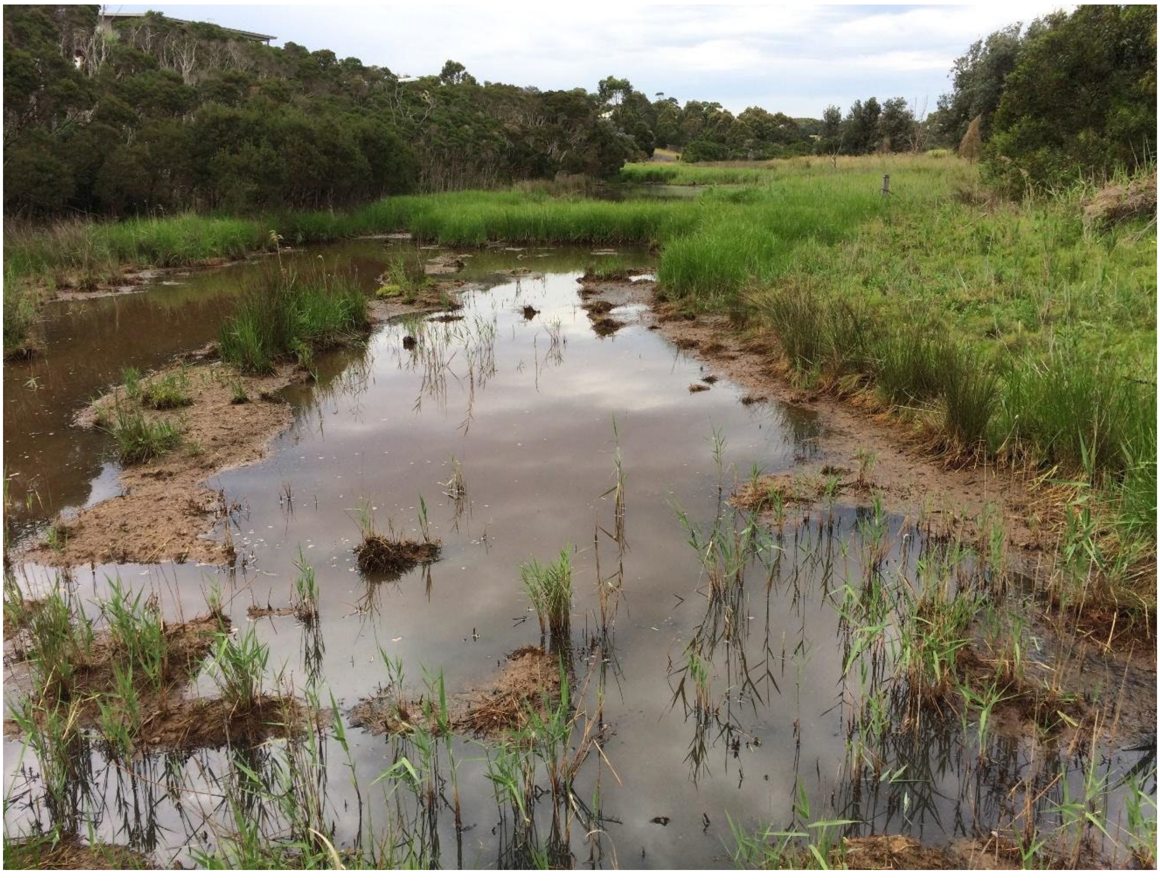

Sandy Cove, Victoria (-38.3912737, 142.2419961)

Brackish wetland disconnected from Moyne estuary (supra-tidal) with saltmarsh 0.3-0.5 m and fringing shrubs.

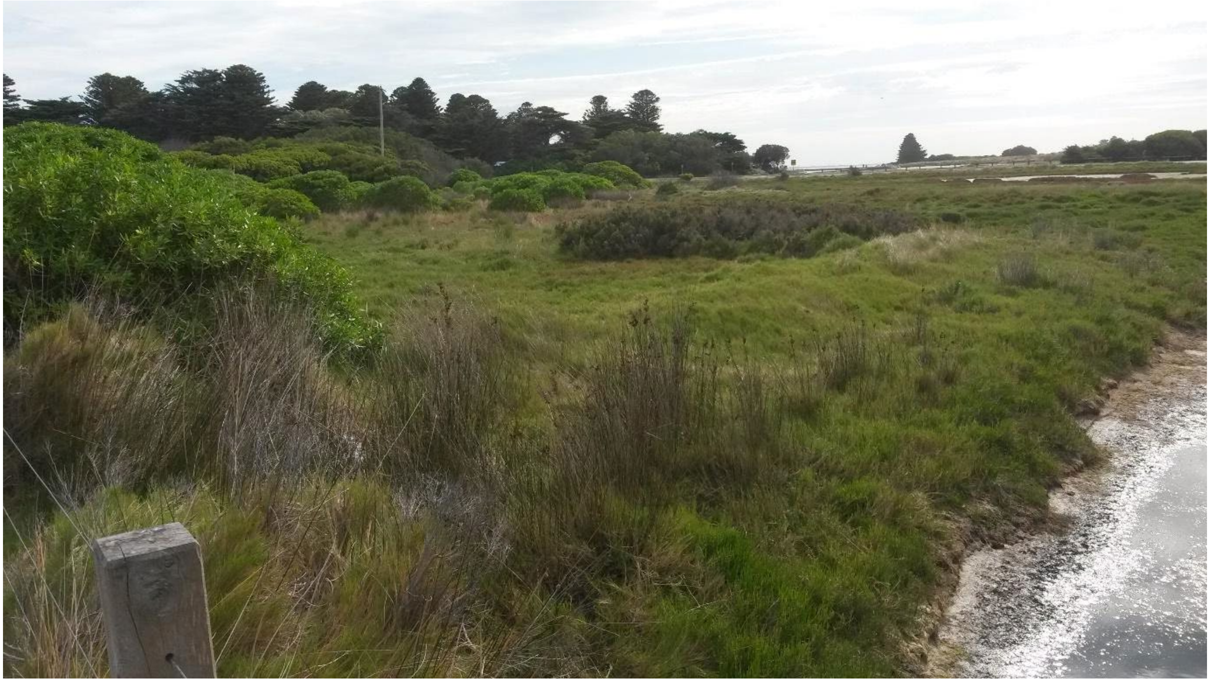

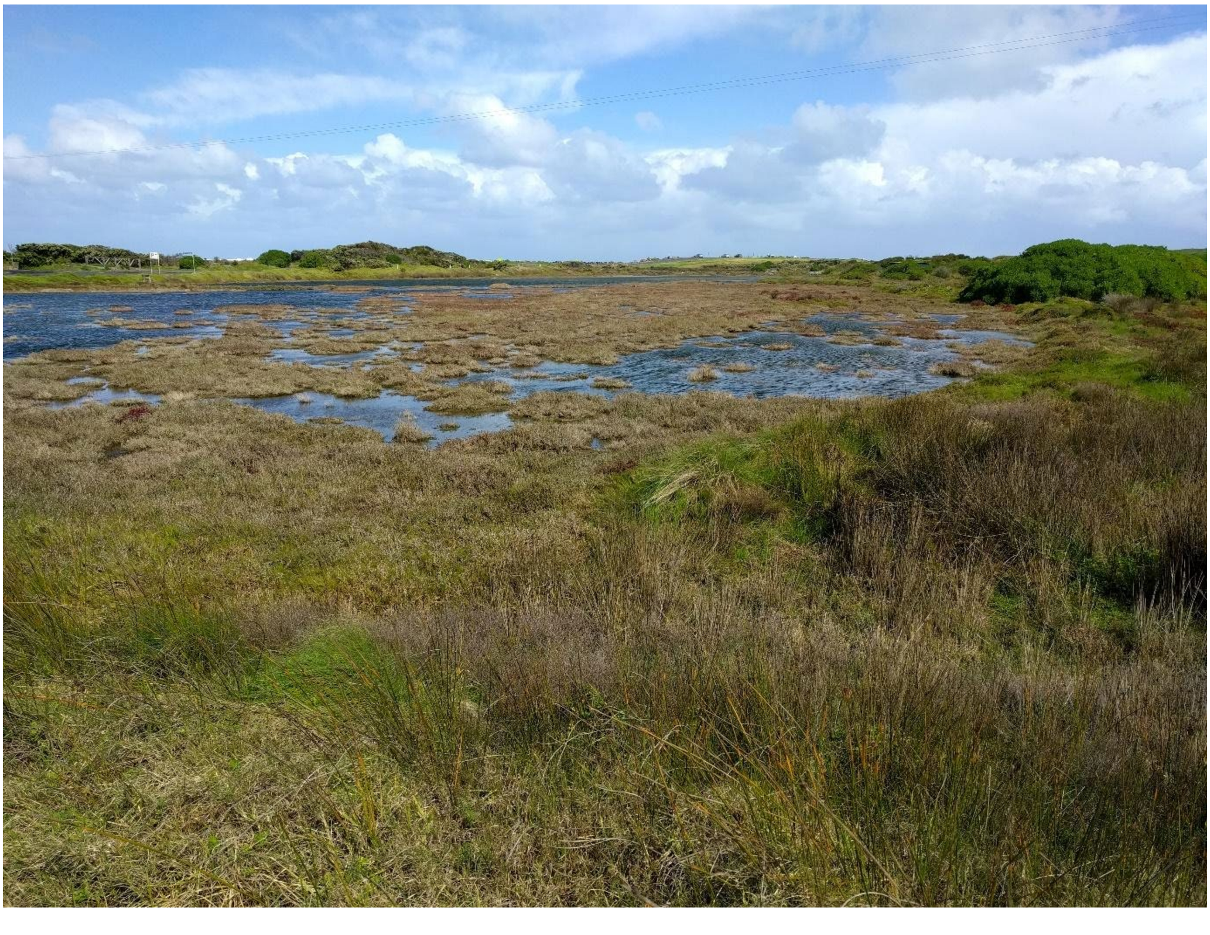

Silverleaves / Honeysuckle Grove, Victoria (-38.4536236, 145.2676456)

Freshwater graduating to saline reedy and grassy drainage reserve with kikuyu <50cm, saltmarsh 10-20 cm and reeds 2-3 m. Fringing vegetation is Banksia woodland, urban gardens; periodically mown. Photos courtesy of Jon Fallaw, Roz Jessop and Sharon Woodend.

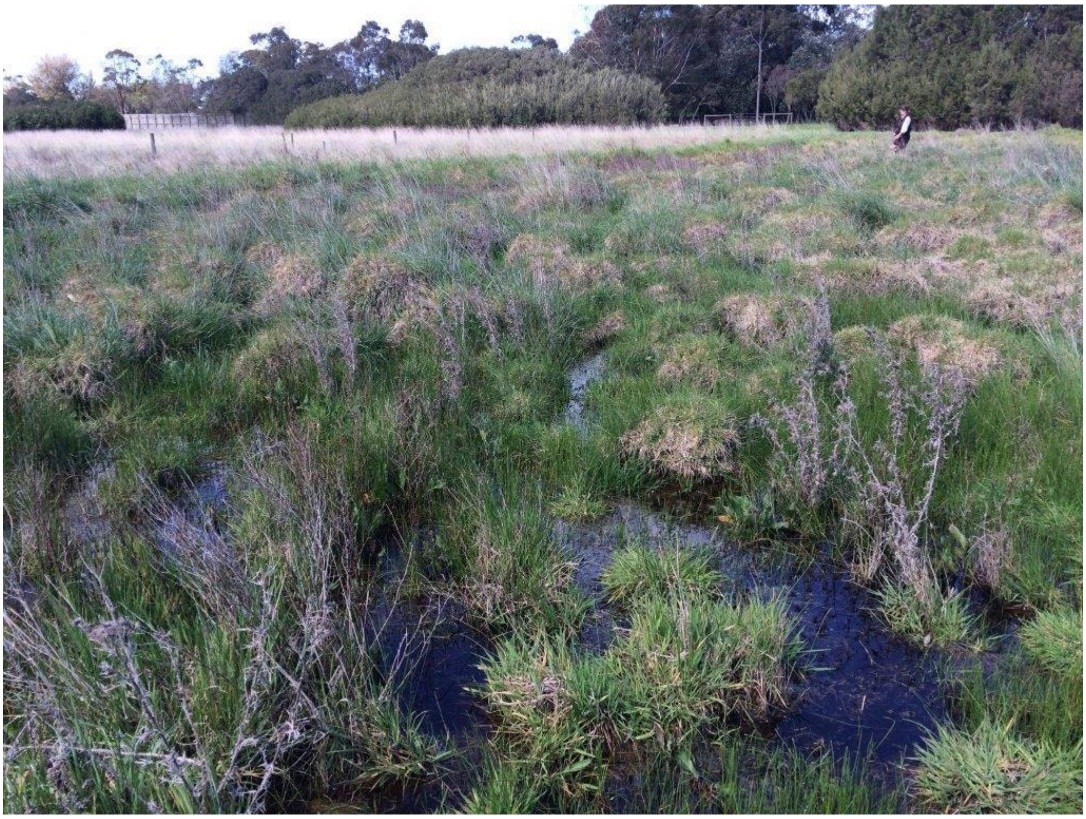

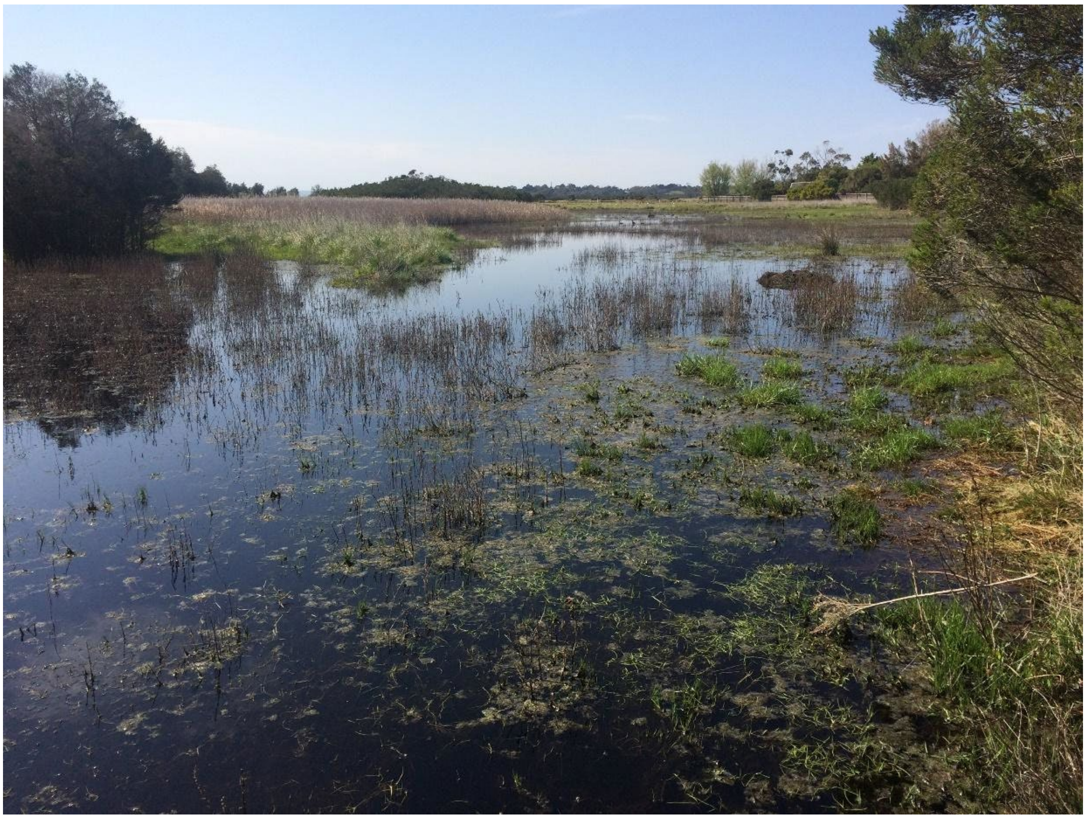

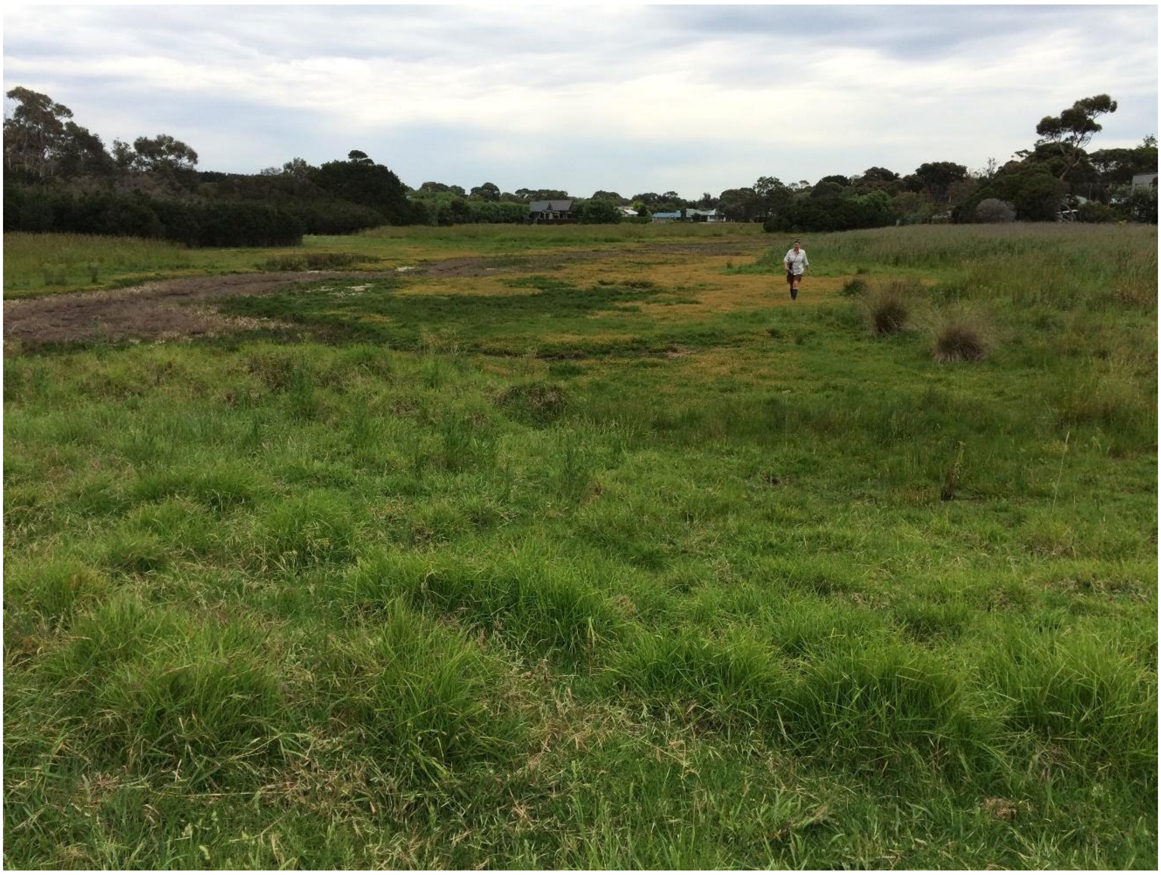

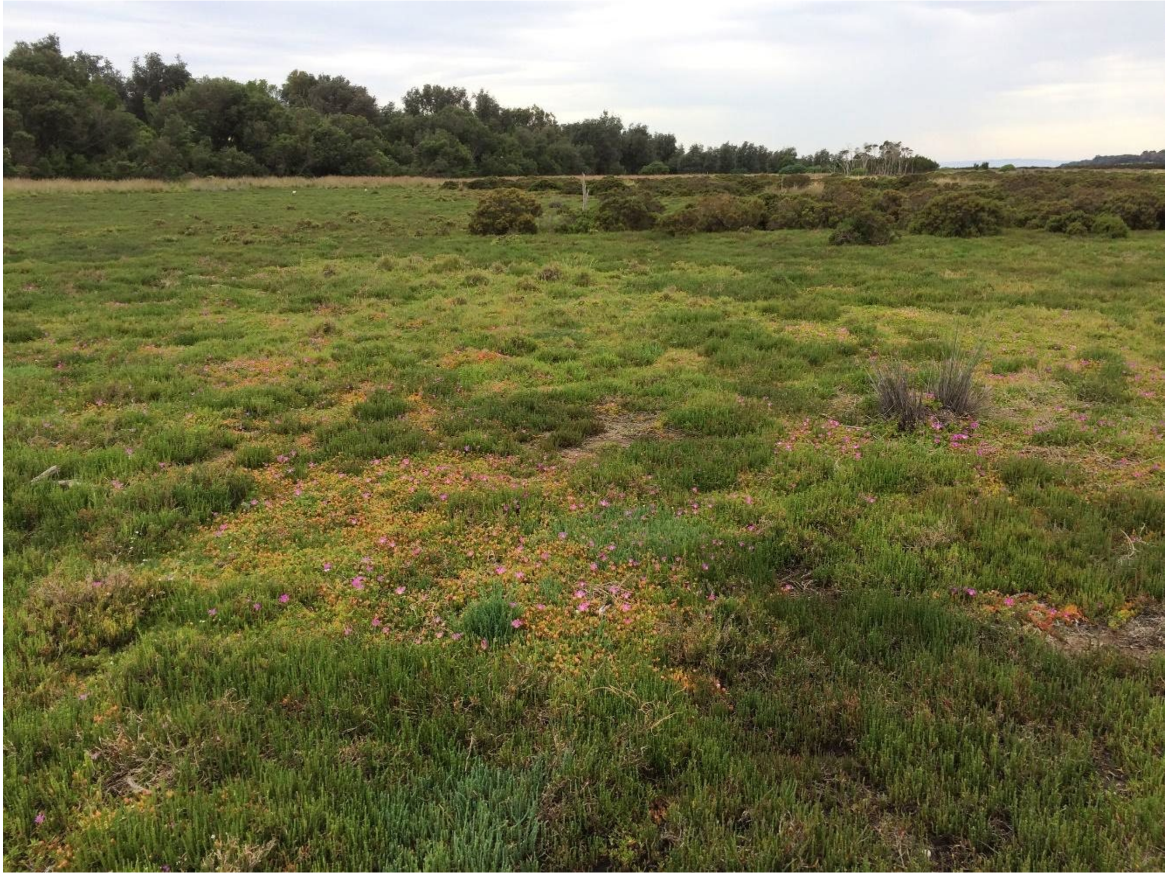

Tirhatuan Wetlands Conservation Reserve, Victoria (-37.9391871, 145.2224761)

Modified freshwater wetland containing patchy emergent Juncus and Baumea tussocks, myriophyllum and grasses 0.2-0.8m; fringing vegetation reeds and *Leptospermum continentale* to 2-3m high, with a ground cover of *Centella cordifolia* . Grassy areas often mown. Photos courtesy of Knud Hansen.

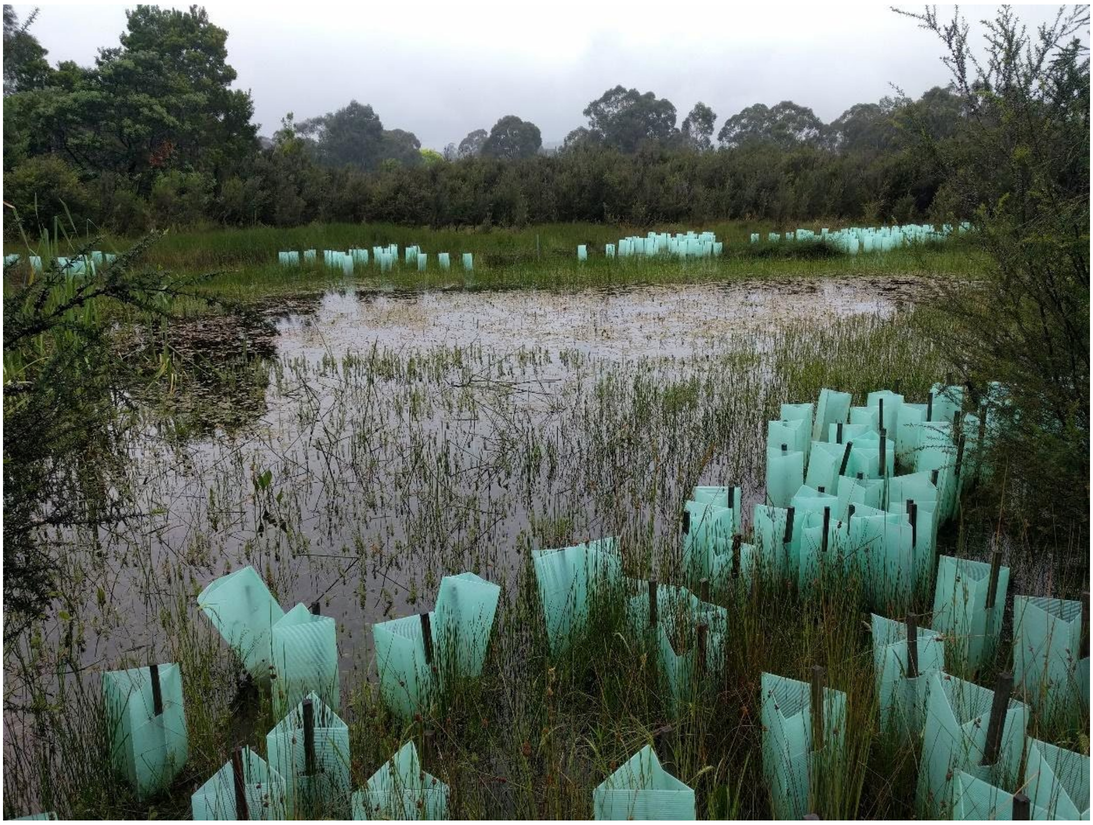

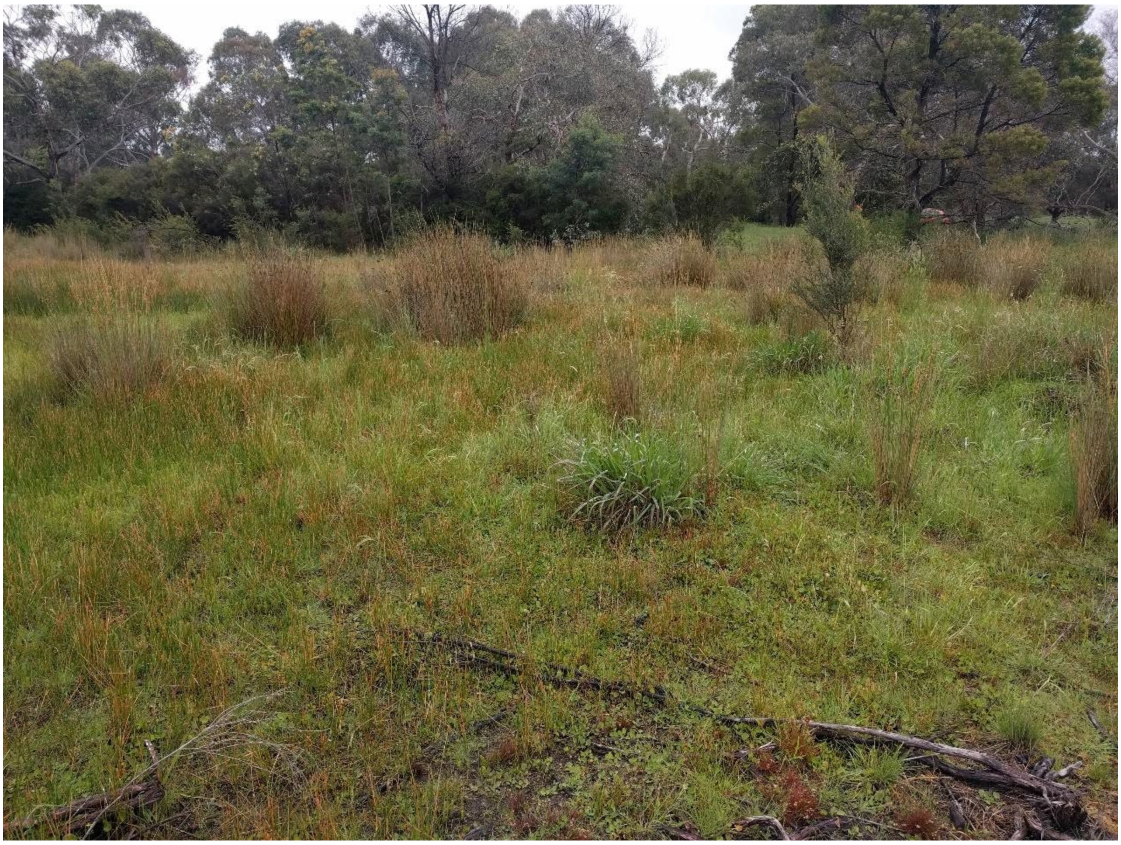

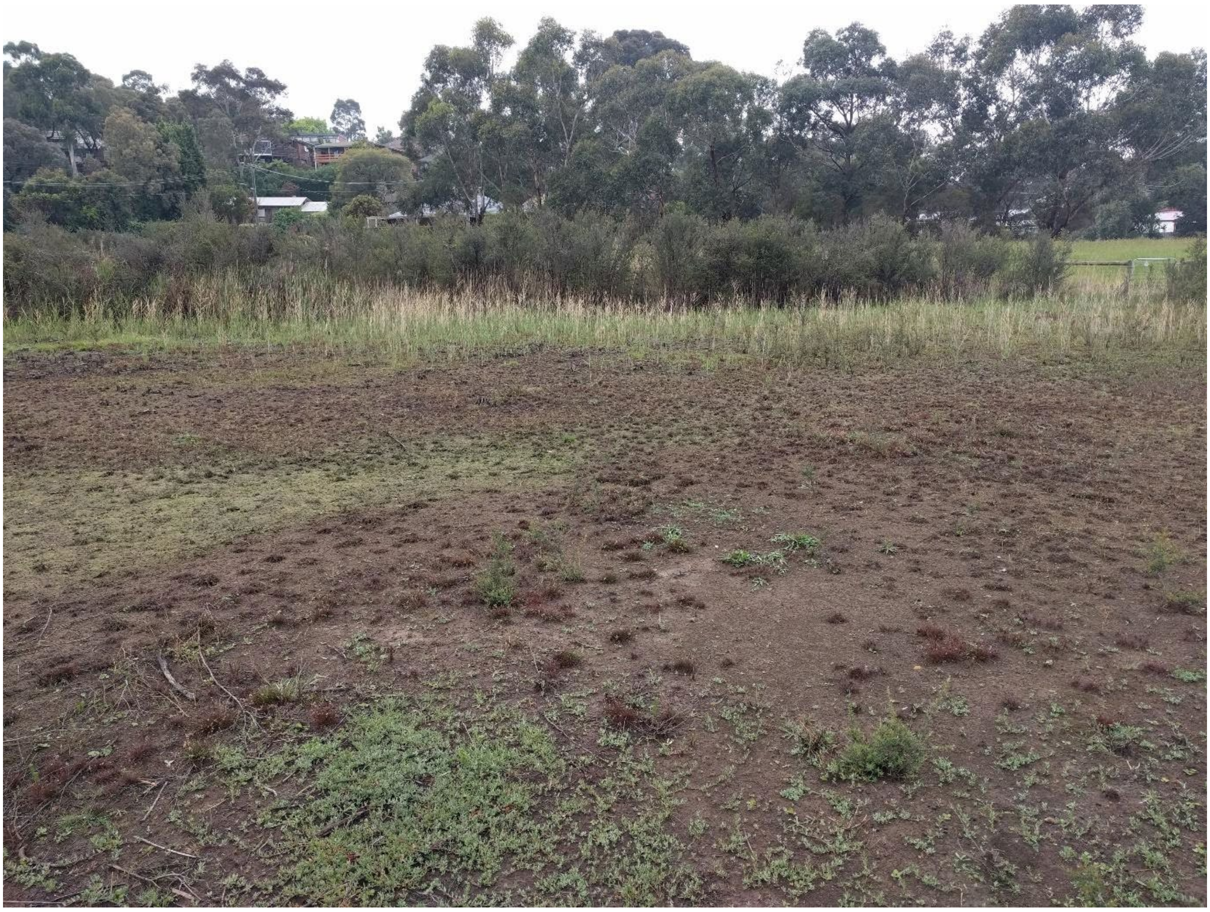

Waterford wetlands, Victoria (-37.909671, 145.2614668)

Freshwater retention basin wetlands with tussocks and introduced grasses 0.1-0.5 m high, but in some areas and near reeds 1.5 m high. Scattered trees and shrubs adjacent to a golf course.

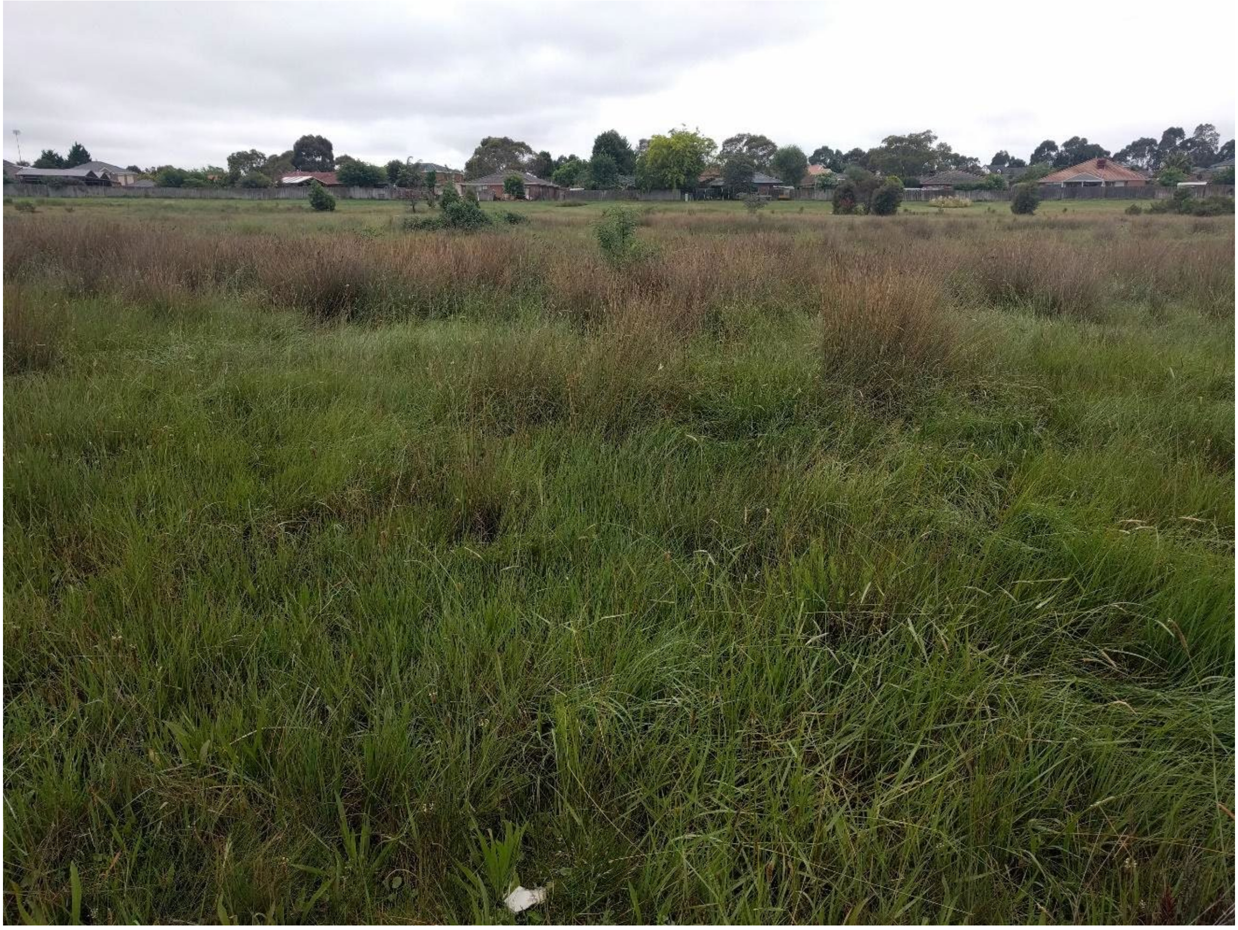

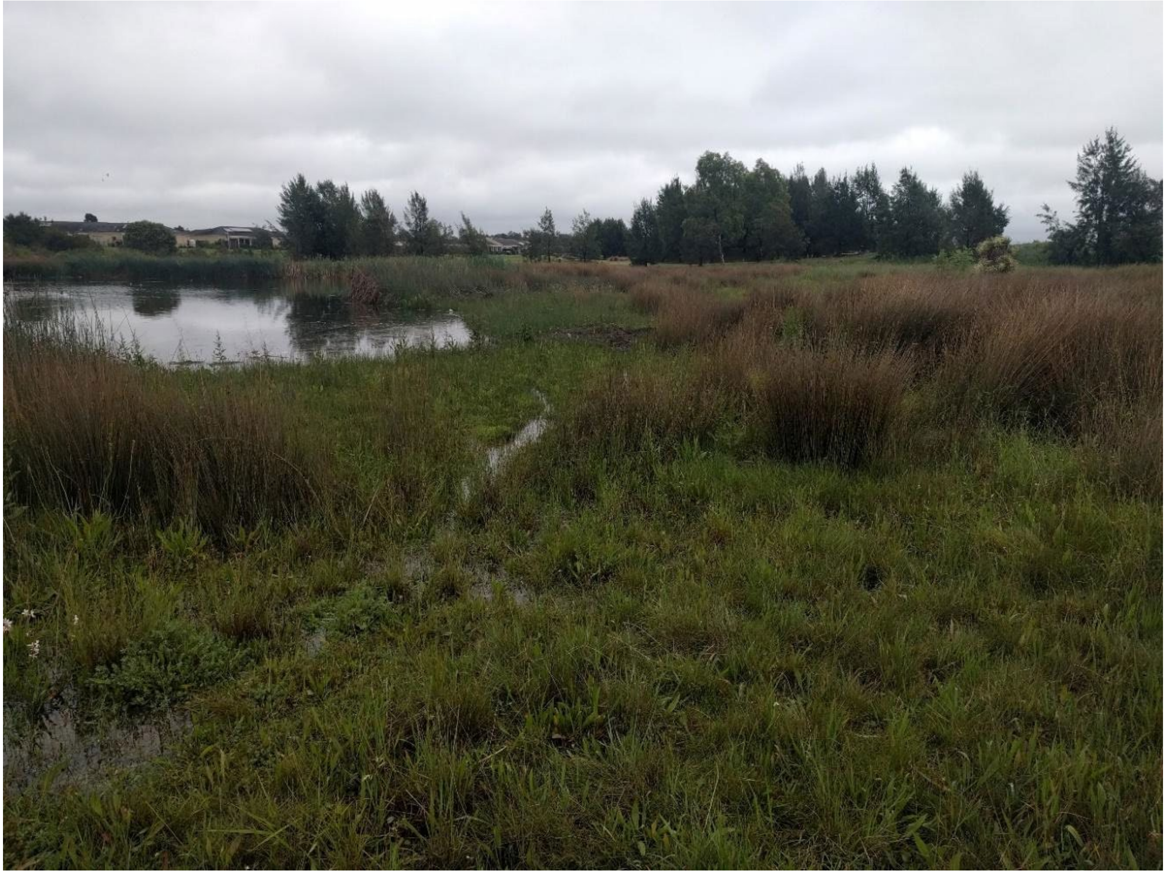

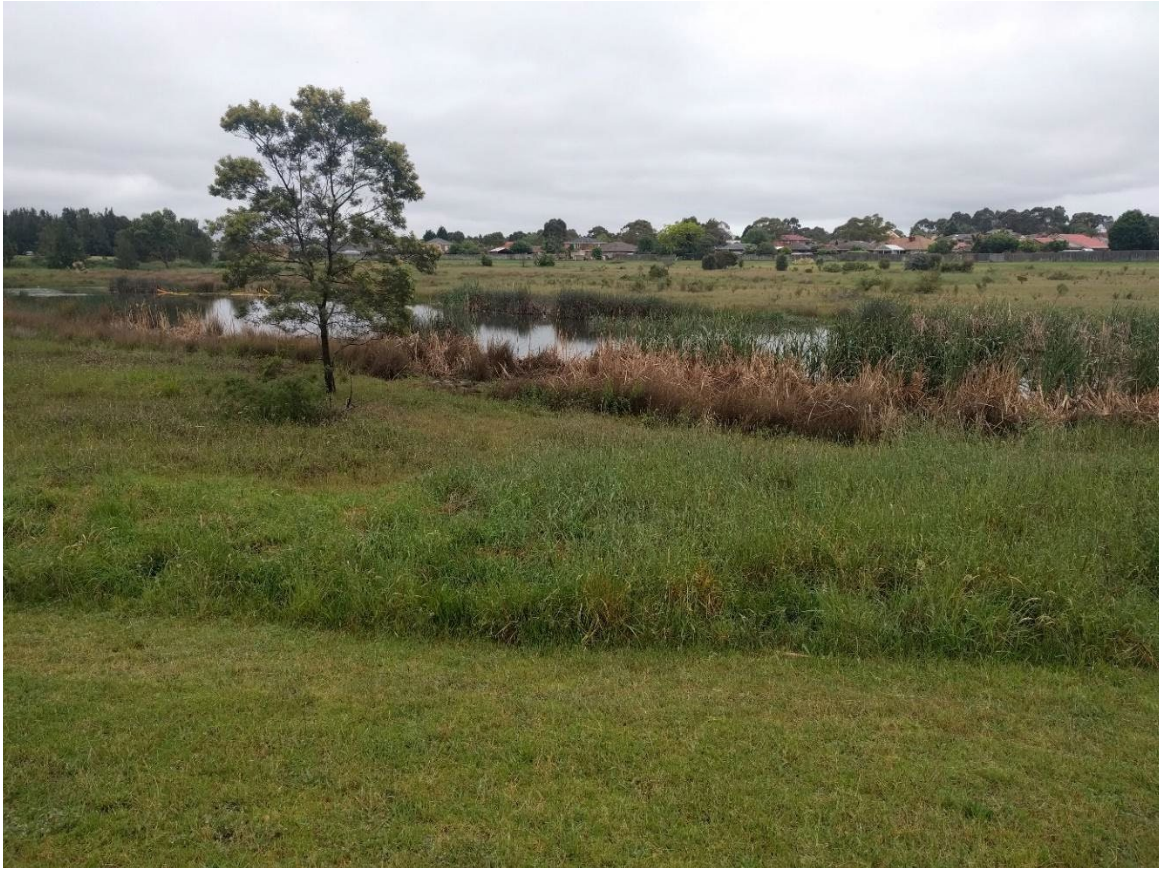

Table S2. Flight initiation distances (FID) for Latham's Snipe from a selection of wetlands sites in the national survey program. Estimates of alert distances (AD) were derived using raw FID values from this study with three different analytical approaches: (1) Latham's Snipe dataset regression of AD versus FID for eight snipe (unpubl. data), (2) cross-species AD-FID regression using the raw 95th percentile AD and FID, and (2b) cross-species AD-FID regression using the adjusted 95th percentile FID.

Site name	State	Land use	No. Observers	Mean FID (SD)	No FID records	Raw 95th FID	Adj. 95th FID	AD est 1	AD est 2	AD est 2b
Allansford	VIC	Urban	5	19.0 (13.5)	145	49.0	41.3	53.6	67.7	61.5
Burton's Reserve	TAS	Urban	1	7.0 (3.0)	23	10.5	12.0	11.4	13.1	20.5
Byron wetlands	NSW	Urban	2	21.1 (18.6)	80	50.0	41.4	54.7	69.1	61.6
Cheltenham Rd Retarding Basin	VIC	Urban	1	24.7 (17.3)	87	50.0	53.2	54.7	69.1	78.2
Fox & Pub Lakes	SA	Urban	2	31.5 (24.2)	39	77.5	71.3	84.8	108.1	103.4
Melton Botanic Gardens	VIC	Urban	3	14.6 (7.6)	20	30.0	27.1	32.8	40.8	41.7
Moyne estuary	VIC	Urban	7	22.6 (17.8)	41	50.0	51.9	54.7	69.1	76.3
Peterborough wetlands	VIC	Urban	5	21.5 (13.5)	356	50.0	43.7	54.7	69.1	64.9
Powling Street wetlands	VIC	Urban	4	28.2 (21.3)	202	70.0	63.2	76.6	97.5	92.1
Railway Place wetland	VIC	Urban	7	16.5 (11.6)	39	41.0	35.6	44.8	56.4	53.5
Retarding Basins Dandenong	VIC	Urban	1	17.2 (12.7)	37	42.0	38.1	45.9	57.8	57.1
Saltwater Creek	VIC	Urban	5	16.4 12.9)	38	40.0	37.7	43.7	55.0	56.4
Sandy Cove	VIC	Urban	5	24.2 (10.9)	77	46.0	42.1	50.3	63.5	62.6
Silverleaves	VIC	Urban	3	24.3 (18.6)	27	43.5	54.8	47.6	59.9	80.4
Tirhatuan Wetlands Conservation Reserve	VIC	Urban	1	22.9 (17.5)	36	52.5	51.6	57.4	72.7	75.9
Waterford Wetlands	VIC	Urban	1	20.2 (9.8)	63	40.0	36.3	43.7	55.0	54.5
Butcher Gap Conservation Park	SA	Non-urban	1	27.2 (18.6)	128	60.0	57.9	65.6	83.3	84.6
Cape Paterson Ecovillage	VIC	Non-urban	1	22.7 (11.5)	25	40.0	41.5	43.7	55.0	61.8
Clifton Creek swamp	VIC	Non-urban	1	25.2 (17.4)	44	50.0	53.9	54.7	69.1	79.1
Heart Morass	VIC	Non-urban	1	26.7 (9.9)	22	40.0	43.0	43.7	55.0	63.9
All sites combined				22.8 (16.1)	1529	50.0	49.3	69.1	72.7	54.7

Figure S3. Regression of alert distance (AD) versus flight initiation distance (FID) from Latham's Snipe observations (n = 8) in a previous study (D. Blumstein, unpubl. data) used to provide estimated buffer width.

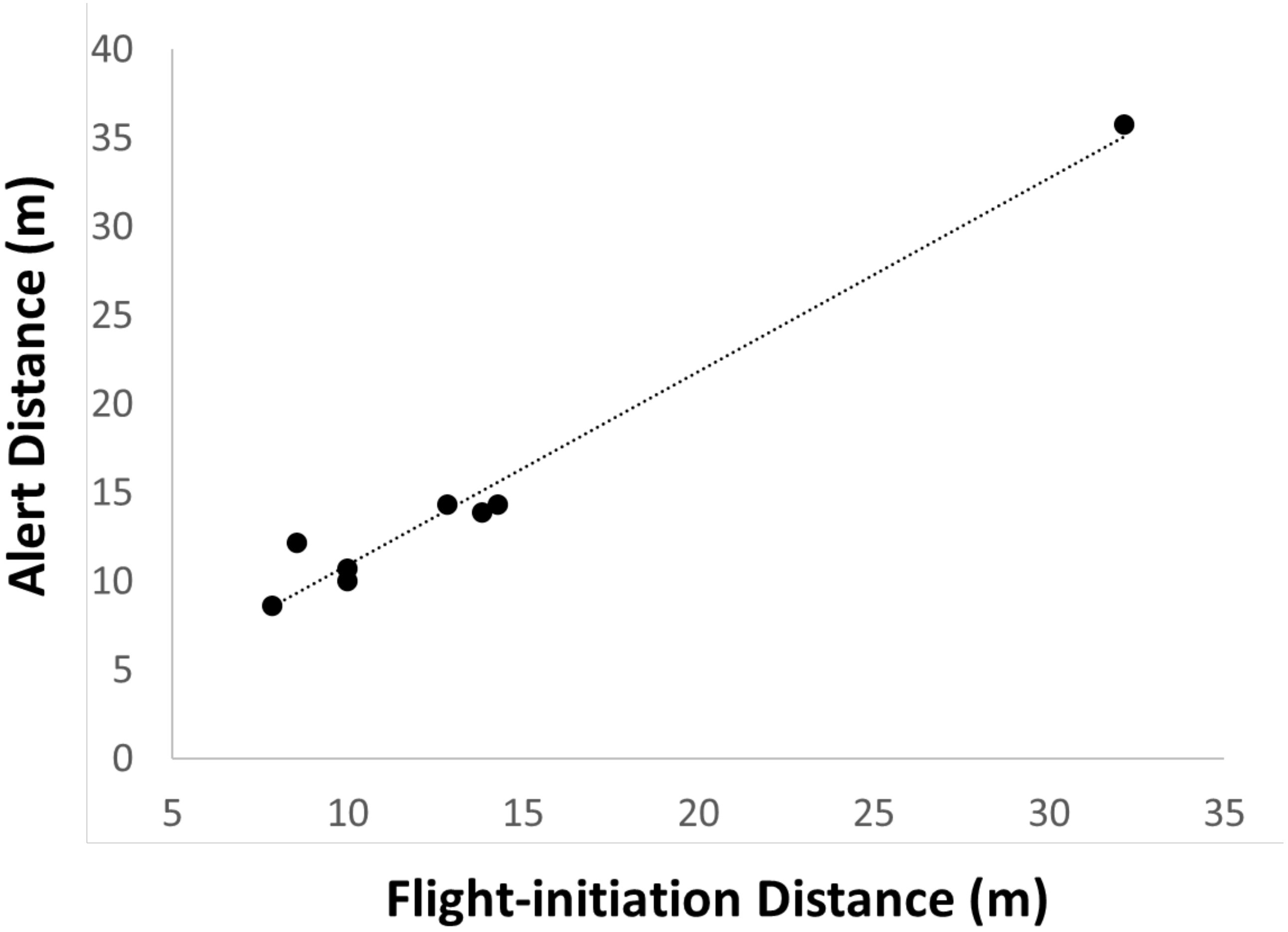

Figure S4. Regressions of alert distance (AD) versus flight initiation distance (FID) from eight Scolopacids used to provide estimated buffer width. Different symbols represent the two different FID metrics (blue triangle and dashed line = raw 95th percentile; black circle and solid line = adjusted 95th percentile).

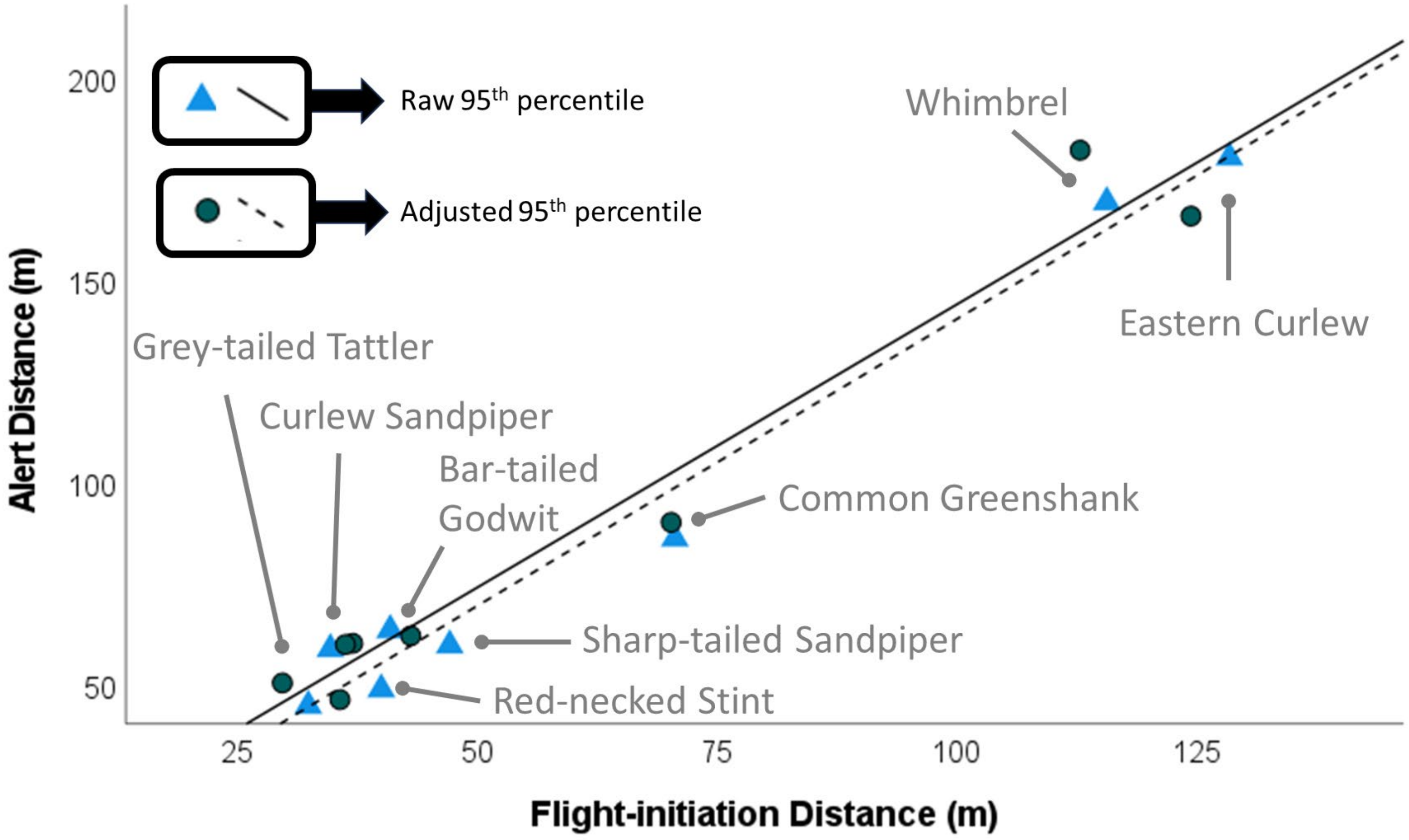

Table S5. Estimates of AD (median, 5%, 80%, 90% and 95% confidence intervals) for each of three different analytical approaches (See Table S1) derived using 10,000 bootstrapped replicates of raw FID.

**Site**	**Land use**	**est1_median**	**est1_5**	**est1_80**	**est1_90**	**est1_95**	**est2a_median**	**est2a_5**	**est2a_80**	**est2a_90**	**est2a_95**	**est2b_median**	**est2b_5**	**est2b_80**	**est2b_90**	**est2b_95**
Allansford	Urban	40.2	36.3	45.8	47.6	49.3	50.4	45.4	57.6	60.0	62.1	57.0	51.8	59.8	61.2	62.3
Burton's Reserve	Urban	11.4	10.9	15.8	15.9	16.4	13.1	12.5	18.8	18.9	19.5	20.3	17.7	21.7	22.4	23.0
Byron wetlands	Urban	53.6	42.9	53.8	53.9	53.9	67.7	53.9	68.1	68.1	68.1	61.0	54.3	64.4	66.2	67.7
Cheltenham Rd Retarding Basin	Urban	54.7	51.4	87.5	87.5	87.5	69.1	64.9	111.6	111.6	111.6	77.5	64.1	84.3	87.7	90.8
Fox & Pub Lakes	Urban	84.7	55.6	105.5	105.5	105.5	108.0	70.3	134.9	134.9	134.9	101.3	80.1	111.6	116.8	121.0
Melton Botanic Gardens	Urban	24.8	19.8	26.7	26.7	26.7	30.5	24.0	33.0	33.0	33.0	37.3	30.6	40.0	41.4	42.4
Moyne estuary	Urban	41.6	31.9	66.0	68.2	69.3	52.2	39.6	83.7	86.6	88.1	68.0	48.7	77.4	82.1	86.3
Peterborough wetlands	Urban	48.1	44.8	52.0	54.4	55.9	60.6	56.4	65.7	68.7	70.7	61.9	58.4	63.6	64.6	65.4
Powling Street wetlands	Urban	68.2	57.4	81.2	87.7	91.0	86.6	72.6	103.5	111.9	116.2	87.9	78.7	92.7	95.2	97.2
Railway Place wetland	Urban	29.2	23.2	32.0	33.4	34.3	36.2	28.3	39.8	41.6	42.8	45.7	37.7	49.5	51.5	52.9
Retarding Basins Dandenong Valley Hwy	Urban	45.9	30.6	56.9	56.9	65.6	57.8	38.0	72.0	72.0	83.3	56.6	42.4	63.1	66.5	69.0
Saltwater Creek	Urban	37.0	26.2	42.2	45.6	47.8	46.2	32.3	53.0	57.4	60.3	51.3	38.7	57.0	59.8	61.8
Sandy Cove	Urban	44.7	39.5	46.8	47.6	48.3	56.2	49.5	58.9	59.9	60.9	60.9	55.9	63.3	64.7	65.6
Silverleaves	Urban	49.6	33.2	56.4	58.1	58.7	62.6	41.3	71.4	73.6	74.4	72.1	50.7	81.0	84.8	88.1
Tirhatuan Wetlands Conservation Reserve	Urban	56.1	32.8	68.4	76.6	109.4	70.9	40.8	86.8	97.5	140.0	73.8	51.1	86.7	93.0	98.6
Waterford Wetlands	Urban	43.7	32.8	43.7	43.7	43.7	55.0	40.8	55.0	55.0	55.0	54.1	48.8	56.9	58.4	59.6
Butcher Gap Conservation Park	Non-urban	65.6	58.2	72.8	79.9	87.5	83.3	73.7	92.5	101.7	111.6	84.3	75.7	88.9	91.4	93.4
Cape Paterson Ecovillage	Non-urban	43.7	37.2	56.9	56.9	60.2	55.0	46.5	72.0	72.0	76.2	60.8	49.9	66.8	69.9	72.2
Clifton Creek swamp	Non-urban	54.7	43.7	54.7	54.7	101.2	69.1	55.0	69.1	69.1	129.4	77.7	61.4	87.4	92.0	96.7
Heart Morass	Non-urban	43.7	38.3	43.7	43.7	43.7	55.0	47.9	55.0	55.0	55.0	63.4	57.4	66.0	67.4	68.4
**Overall**		**44.6**	**16.4**	**59.8**	**72.8**	**84.7**	**56.1**	**19.5**	**75.7**	**92.5**	**108.0**	**61.6**	**24.7**	**80.6**	**88.6**	**94.9**
